# The house spider genome reveals an ancient whole-genome duplication during arachnid evolution

**DOI:** 10.1186/s12915-017-0399-x

**Published:** 2017-07-31

**Authors:** Evelyn E. Schwager, Prashant P. Sharma, Thomas Clarke, Daniel J. Leite, Torsten Wierschin, Matthias Pechmann, Yasuko Akiyama-Oda, Lauren Esposito, Jesper Bechsgaard, Trine Bilde, Alexandra D. Buffry, Hsu Chao, Huyen Dinh, HarshaVardhan Doddapaneni, Shannon Dugan, Cornelius Eibner, Cassandra G. Extavour, Peter Funch, Jessica Garb, Luis B. Gonzalez, Vanessa L. Gonzalez, Sam Griffiths-Jones, Yi Han, Cheryl Hayashi, Maarten Hilbrant, Daniel S. T. Hughes, Ralf Janssen, Sandra L. Lee, Ignacio Maeso, Shwetha C. Murali, Donna M. Muzny, Rodrigo Nunes da Fonseca, Christian L. B. Paese, Jiaxin Qu, Matthew Ronshaugen, Christoph Schomburg, Anna Schönauer, Angelika Stollewerk, Montserrat Torres-Oliva, Natascha Turetzek, Bram Vanthournout, John H. Werren, Carsten Wolff, Kim C. Worley, Gregor Bucher, Richard A. Gibbs, Jonathan Coddington, Hiroki Oda, Mario Stanke, Nadia A. Ayoub, Nikola-Michael Prpic, Jean-François Flot, Nico Posnien, Stephen Richards, Alistair P. McGregor

**Affiliations:** 1 Department of Biological and Medical Sciences, Oxford Brookes University, Gipsy Lane, Oxford, OX3 0BP UK; 2 Department of Biological Sciences, University of Massachusetts Lowell, 198 Riverside Street, Lowell, MA 01854 USA; 3 Department of Zoology, University of Wisconsin-Madison, 430 Lincoln Drive, Madison, WI 53706 USA; 4 Department of Biology, Washington and Lee University, 204 West Washington Street, Lexington, VA 24450 USA; 5 Department of Biology, University of California, Riverside, Riverside, CA 92521 USA; 6 J. Craig Venter Institute, 9714 Medical Center Drive, Rockville, MD 20850 USA; 7 Ernst Moritz Arndt University Greifswald, Institute for Mathematics and Computer Science, Walther-Rathenau-Str. 47, 17487 Greifswald, Germany; 8 Department for Developmental Biology, University Goettingen, Johann-Friedrich-Blumenbach-Institut for Zoology and Anthropology, GZMB Ernst-Caspari-Haus, Justus-von-Liebig-Weg 11, 37077 Goettingen, Germany; 9 Department of Developmental Biology, University of Cologne, Cologne Biocenter, Institute of Zoology, Zuelpicher Straße 47b, 50674 Cologne, Germany; 10 JT Biohistory Research Hall, 1-1 Murasaki-cho, Takatsuki, Osaka 569-1125 Japan; 11 Osaka Medical College, Takatsuki, Osaka Japan; 12 Institute for Biodiversity Science and Sustainability, California Academy of Sciences, 55 Music Concourse Drive, San Francisco, CA 94118 USA; 13 Department of Bioscience, Aarhus University, Ny Munkegade 116, building 1540, 8000 Aarhus C, Denmark; 14 Human Genome Sequencing Center, Department of Molecular and Human Genetics, Baylor College of Medicine, One Baylor Plaza, Houston, TX 77030 USA; 15 Department of Genetics, Friedrich-Schiller-University Jena, Philosophenweg 12, 07743 Jena, Germany; 16 Department of Organismic and Evolutionary Biology, Harvard University, 16 Divinity Avenue, Cambridge, MA 02138 USA; 17 Smithsonian National Museum of Natural History, MRC-163, P.O. Box 37012, Washington, DC, 20013-7012 USA; 18 Faculty of Biology Medicine and Health, University of Manchester, D.1416 Michael Smith Building, Oxford Road, Manchester, M13 9PT UK; 19 Division of Invertebrate Zoology, American Museum of Natural History, New York, NY 10024 USA; 20 Department of Earth Sciences, Palaeobiology, Uppsala University, Villavägen 16, 75236 Uppsala, Sweden; 21 Centro Andaluz de Biología del Desarrollo (CABD), Consejo Superior de Investigaciones Científicas/Universidad Pablo de Olavide, Sevilla, Spain; 22 Nucleo em Ecologia e Desenvolvimento SocioAmbiental de Macaé (NUPEM), Campus Macaé, Universidade Federal do Rio de Janeiro (UFRJ), Rio de Janeiro, 27941-222 Brazil; 23 School of Biological and Chemical Sciences, Queen Mary University of London, Mile End Road, E1 4NS London, UK; 24 Evolution and Optics of Nanostructure group (EON), Biology Department, Ghent University, Gent, Belgium; 25 Biology Department, University of Rochester, Rochester, NY 14627 USA; 26 Humboldt-Universität of Berlin, Institut für Biologie, Philippstr.13, 10115 Berlin, Germany; 27 Department of Evolutionary Developmental Genetics, Johann-Friedrich-Blumenbach-Institute, GZMB, Georg-August-University, Göttingen Campus, Justus von Liebig Weg 11, 37077 Göttingen, Germany; 28 Department of Biological Sciences, Graduate School of Science, Osaka University, Osaka, Japan; 29 Université libre de Bruxelles (ULB), Evolutionary Biology & Ecology, C.P. 160/12, Avenue F.D. Roosevelt 50, 1050 Brussels, Belgium

**Keywords:** *Parasteatoda tepidariorum*, Genome, *Centruroides sculpturatus*, Gene duplication, Evolution, Hox genes

## Abstract

**Background:**

The duplication of genes can occur through various mechanisms and is thought to make a major contribution to the evolutionary diversification of organisms. There is increasing evidence for a large-scale duplication of genes in some chelicerate lineages including two rounds of whole genome duplication (WGD) in horseshoe crabs. To investigate this further, we sequenced and analyzed the genome of the common house spider *Parasteatoda tepidariorum*.

**Results:**

We found pervasive duplication of both coding and non-coding genes in this spider, including two clusters of Hox genes. Analysis of synteny conservation across the *P. tepidariorum* genome suggests that there has been an ancient WGD in spiders. Comparison with the genomes of other chelicerates, including that of the newly sequenced bark scorpion *Centruroides sculpturatus*, suggests that this event occurred in the common ancestor of spiders and scorpions, and is probably independent of the WGDs in horseshoe crabs. Furthermore, characterization of the sequence and expression of the Hox paralogs in *P. tepidariorum* suggests that many have been subject to neo-functionalization and/or sub-functionalization since their duplication.

**Conclusions:**

Our results reveal that spiders and scorpions are likely the descendants of a polyploid ancestor that lived more than 450 MYA. Given the extensive morphological diversity and ecological adaptations found among these animals, rivaling those of vertebrates, our study of the ancient WGD event in Arachnopulmonata provides a new comparative platform to explore common and divergent evolutionary outcomes of polyploidization events across eukaryotes.

**Electronic supplementary material:**

The online version of this article (doi:10.1186/s12915-017-0399-x) contains supplementary material, which is available to authorized users.

## Background

Gene duplication plays an important role in the evolutionary diversification of organisms [1, 2]. Unequal crossing-over commonly results in one or a few tandemly duplicated genes, but larger scale events, including whole genome duplications (WGDs) can also occur. Tandem duplication has been shown to underlie the evolution of many genes in both plants and animals, for example, of up to 32% of genes in the centipede *Strigamia maritima* [3, 4]. WGD is arguably the most sudden and massive change that a genome can experience in a single evolutionary event. The occurrence of WGDs across a wide variety of eukaryotic groups, including plants [5, 6], fungi [7, 8], ciliates [9], oomycetes [10], and animals [11–17], attests to the major impact that polyploidization events have had in reshaping the genomes of many different organisms.

Although most of the duplicated genes resulting from tandem duplication or WGD are subsequently lost, it is thought that these events provide new genetic material for some paralogous genes to undergo sub-functionalization or neo-functionalization and thus contribute to the rewiring of gene regulatory networks, morphological innovations and, ultimately, organismal diversification [2, 7, 18–24]. Comparisons of independent paleopolyploidization events across different eukaryotes, such as plants, yeast, and vertebrates [5, 8, 11, 13, 14, 24], have led to the development of models to elucidate genome-wide evolutionary patterns of differential gene loss and retention compared to smaller-scale events [2, 25]. However, the enormous differences between these disparate eukaryotic lineages in terms of genome structure, morphological and developmental organization, and ecology have impeded a critical assessment of the potential selective advantages and actual evolutionary consequences of WGDs. Thus, the extent to which WGDs may have contributed to taxonomic “explosions” and evolutionary novelties remains controversial, especially in the case of vertebrates [26–28]. For example, the two WGDs shared by all vertebrates have given rise to four clusters of Hox genes, providing new genetic material that may underlie the evolutionary success and innovations among these animals [24, 29, 30]. However, only three WGD events have been demonstrated in animals other than vertebrates, namely one in bdelloid rotifers and possibly two in horseshoe crabs [11, 14, 31], and these events are not associated with any bursts of diversification [32, 33]. It is clear, therefore, that documenting additional examples of WGD in metazoans would significantly increase our understanding of the genomic and morphological consequences of these events.

**Fig. 1:**
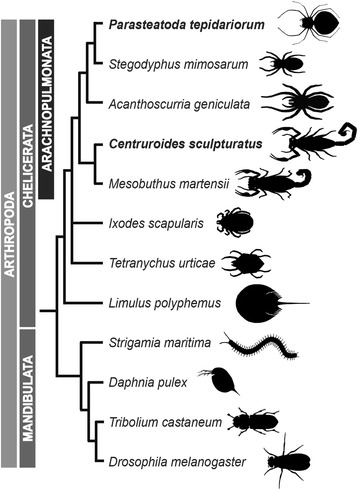
The relationships of *Parasteatoda tepidariorum* to select arthropods. Representatives of spiders (Araneae) with sequenced genomes (*P. tepidariorum*, *Stegodyphus mimosarum*, and *Acanthoscurria geniculata*) are shown with respect to other chelicerates with sequenced genomes including scorpions (*Centruroides sculpturatus* and *Mesobuthus martensii*), a tick (*Ixodes scapularis*), a mite (*Tetranychus urticae*), and a horseshoe crab (*Limulus polyphemus*) as well as representatives of Myriapoda (*Strigamia maritima*), Crustacea (*Daphnia pulex*), and Insecta (*Drosophila melanogaster*). Topology is based on Sharma et al. [53]

Intriguingly, there is increasing evidence for extensive gene duplication among chelicerates other than horseshoe crabs, particularly in spiders and scorpions [34–44], indicating that large-scale gene duplications occurred during the evolution of these arachnids. However, although the genomes of some arachnids have been sequenced, including the tick *Ixodes scapularis* [45, 46], the mite *Tetranychus urticae* [47], the Chinese scorpion *Mesobuthus martensii* [48], and three spiders (the velvet spider *Stegodyphus mimosarum* [49], the Brazilian whiteknee tarantula *Acanthoscurria geniculata* [49], and the golden orb-weaver *Nephila clavipes* [50]), a systematic analysis of genome evolution among these diverse animals has yet to be performed (Fig. 1) [51].

As a step towards this goal, we herein report the sequencing and analysis of the genomes of the common house spider *Parasteatoda tepidariorum* (C. L. Koch, 1841; formerly *Achaearanea tepidariorum*) [52] and the bark scorpion *Centruroides sculpturatus* (Wood, 1863) (Fig. 1), together with comparative genomic analyses of other available chelicerate genomes. We found that the genome of *P. tepidariorum* contains many paralogous genes, including two Hox gene clusters, which is also the case in other spiders and in scorpions (this work; [36]). These similar patterns of gene duplication between spiders and scorpions are consistent with recent molecular phylogenies, which support a much closer phylogenetic relationship of spiders and scorpions than previously thought, in a clade known collectively as Arachnopulmonata [53] (Fig. 1). We also document extensive divergence in the timing and location of expression of each pair of Hox gene paralogs, suggesting there may be far reaching functional consequences. Furthermore, an analysis of synteny among paralogs across the *P. tepidariorum* genome is consistent with a WGD. Comparison with other chelicerates suggests that this WGD took place in the common ancestor of the Arachnopulmonata and is probably independent of the WGDs in the horseshoe crab lineage.

## Results

### *P. tepidariorum* has many duplicated genes

The final *P. tepidariorum* genome assembly has a size of 1443.9 Mb. The number of predicted protein-coding genes in *P. tepidariorum* (27,990) is consistent with those of another spider, *S. mimosarum* (27,235) [49], as are the numbers of predicted genes of the two scorpions *M. martensii* (32,016) [48] and *C. sculpturatus* (30,456) (this study). Spiders and scorpions have significantly higher numbers of predicted genes than other arachnids such as the mite *Tetranychus urticae* (18,414) [47]. We evaluated the completeness of the *P. tepidariorum* gene set and assessed the extent of gene duplication using 1427 benchmarked universal single-copy ortholog (BUSCO) groups of arthropod genes [54], with input datasets ranging from 2806 (*Strigamia maritima*) to 3031 (*Tribolium castaneum*) putatively single-copy orthologs. For *P. tepidariorum*, the HMMER3 homology search revealed 91% complete single-copy orthologs (C), 41% complete duplicated orthologs (D), and 6.5% fragmented orthologs (F). Only 2% of conserved BUSCO groups from the universal ortholog arthropods database were missing (M) from the assembly. The number of duplicated orthologs was very high compared to *Drosophila melanogaster* (C: 99%, D: 3.7%, F: 0.2%, M: 0.0%, 13,918 genes in total) or *Caenorhabditis elegans* (C: 90%, D: 11%, F: 1.7%, M: 7.5%, 20,447 genes in total).

We then undertook a different approach to further investigate the extent of gene duplication, by estimating the ratios of orthologs in arachnopulmonate and non-arachnopulmonate genomes. Specifically, we compared the *P. tepidariorum* and *C. sculpturatus* genomes to the genomes of four other arthropods with a single Hox cluster and no evidence of large-scale gene duplication (“1X genomes”), including another chelicerate (the tick *Ixodes scapularis*) and three mandibulates (the red flour beetle *T. castaneum*, the crustacean *Daphnia pulex*, and the centipede *S. maritima*). The Orthologous Matrix (OMA) [55] algorithm was used to identify orthologs after pairwise mapping of genomes. The orthology mapping indicated that, depending upon the 1X genome used for comparison, between 7.5% and 20.5% of spider genes that could be mapped to a single mandibulate or tick ortholog had undergone duplication (Additional file 1: Table S1). Using the well-annotated *T. castaneum* genome as the reference, we found that 14.6% (523) of the *P. tepidariorum* genes with a single *T. castaneum* ortholog had undergone duplication (Additional file 1: Table S1). We obtained similar results when comparing the genome of the scorpion *C. sculpturatus* with that of *T. castaneum* (10.1%, 290 genes)*.* However, only 4.9% (175) of *I. scapularis* genes had been duplicated since its divergence from *T. castaneum* (Additional file 1: Table S1)*.* Moreover, higher numbers of 1:1 orthologs were found among 1X genomes than in comparisons that included either the spider or the scorpion genome, which is consistent with a greater degree of paralogy in the spider and scorpion genomes. The highest proportion of duplicated genes in a 1X genome, with reference to *T. castaneum*, was found in *D. pulex* (7.8%), which is known to have a large number of tandemly duplicated gene clusters [56] (Additional file 1: Table S1).

**Fig. 2:**
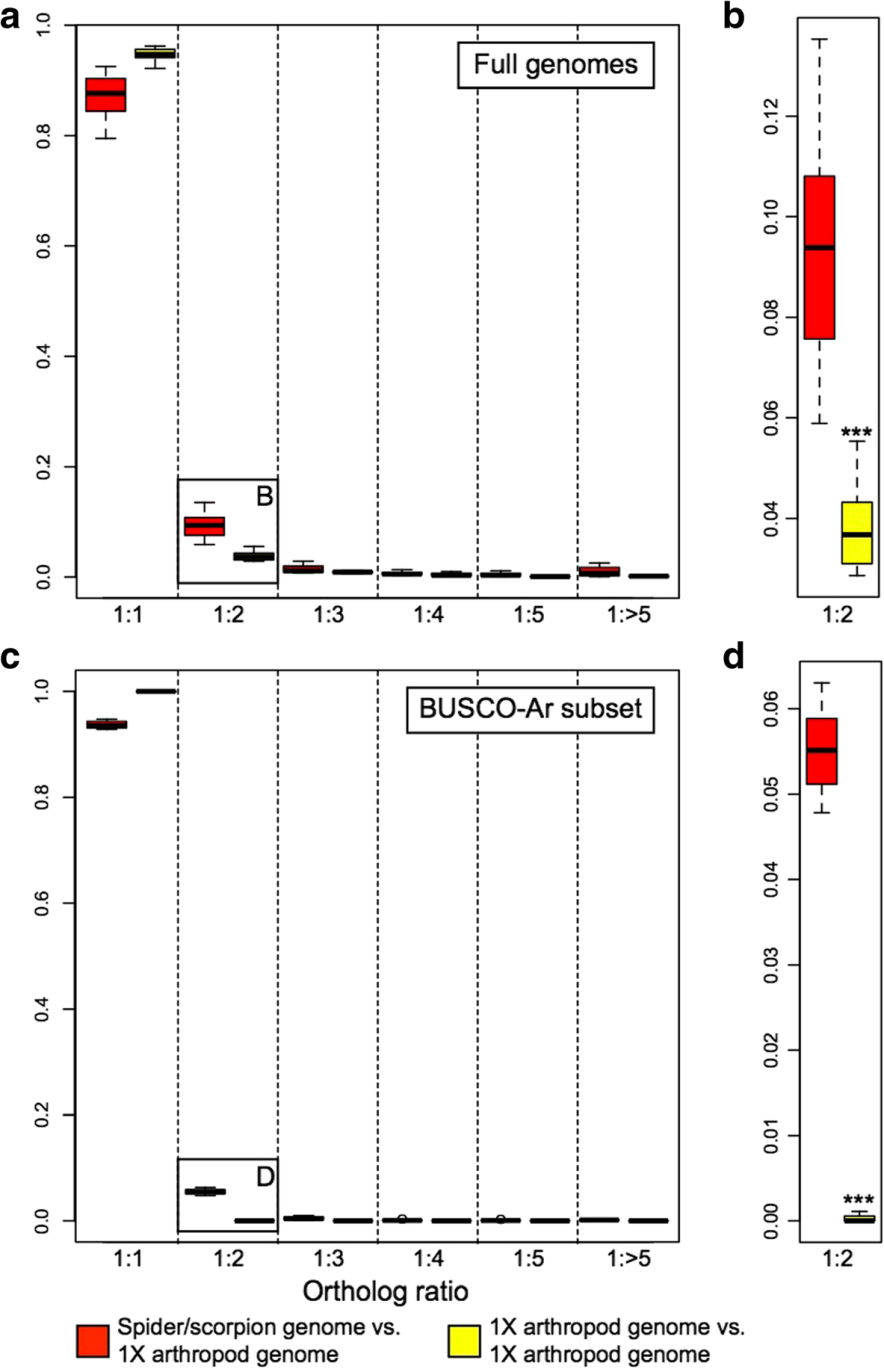
Orthology inference suggests substantial duplication in spiders and scorpions. **a** Distribution of orthology ratios from Orthologous Matrix analysis of full genomes. Comparisons of an arachnopulmonate genome to a 1X genome are shown in red and comparisons among 1X genomes are shown in yellow. A significantly higher number of 1:1 orthologs is recovered in pairwise comparisons within the non-arachnopulmonate genomes (*P* = 1.46 × 10^–3^). **b** Magnification of the 1:2 ortholog ratio category in (**a**) shows a significantly higher number of duplicated genes in comparisons of spider or scorpion genomes to a 1X genome (*P* = 6.67 × 10^–4^). **c** Distribution of orthology ratios for a subset of genes benchmarked as putatively single copy across Arthropoda (BUSCO-Ar). As before, a significantly higher number of 1:1 orthologs is recovered within the 1X genome group (*P* = 3.43 × 10^–8^). **d** Magnification of the 1:2 ortholog ratio category in (**c**) shows a significantly higher number of duplicated genes in spiders and scorpions (*P* = 7.28 × 10^–9^)

Most of the spider and scorpion duplicates occurred in 1:2 paralogy (i.e., two copies in spiders/scorpions for a given mandibulate or tick homolog) (Fig. 2, Additional file 1: Table S1), whereas duplicates in other arthropods showed no particular enrichment for this category. Two-copy duplicates accounted for 5.9–10.9% of the total spider duplicated genes, and 7.4–13.5% of the total scorpion duplicated genes (depending on the mandibulate or tick genome used for comparison). In both cases, these proportions were significantly higher than those of other arthropod genomes (*P* = 6.67 × 10^–4^) (Fig. 2a). Intriguingly, 11.8% of the two-copy duplicates were shared between spiders and scorpions. Inversely, comparing either *P. tepidariorum* or *C. sculpturatus* to mandibulate or tick genomes recovered a much lower proportion of single-copy orthologs (i.e., 1:1) relative to comparisons of any two species of mandibulate or tick. The number of duplicated genes was significantly higher in scorpions and spiders relative to comparing mandibulate or ticks among themselves, and particularly so for the 1:2 paralog bin (two-sample *t*-test; *P* = 3.75 × 10^–4^) (Fig. 2b, Additional file 1: Table S1). We found very similar profiles of paralog distributions using a more conservative approach comparing the spider and scorpion genes to a benchmarked set of 2806–3031 single-copy genes common to arthropods (the BUSCO-Ar database of the OrthoDB project) (Fig. 2c, d). Even within this database of genes with no reported cases of duplication in all other studied arthropods, a considerable fraction of genes was found in two copies in both the *P. tepidariorum* and *C. sculpturatus* genomes (63–78 genes) when compared to the mandibulate or tick datasets (Fig. 2c, d, Additional file 1: Table S1).

### Dispersed and tandem gene duplicates abound in spiders and scorpions

We carried out systematic analysis of the frequency and synteny of duplicated genes in *P. tepidariorum* compared to *C. sculpturatus* and the horseshoe crab *Limulus polyphemus*. The genome of *P. tepidariorum* is characterized by an elevated number of tandem (3726 vs. 1717 and 2066 in *C. sculpturatus* and *L. polyphemus*, respectively) and proximal duplicates (2233 vs. 1114 and 97), i.e., consecutive duplicates and duplicates found at most 10 genes away from their paralog (Additional file 2: Figure S1, Additional file 3: Figure S2, Additional file 4: Figure S3). However, the most salient aspect in all three genomes was the very high number of dispersed duplicates, i.e., genes for which paralogous gene models were detected more than 10 genes apart or on different scaffolds, which amounted to approximately 14,700 genes in each species (Additional file 2: Figure S1, Additional file 3: Figure S2, Additional file 4: Figure S3).

**Fig. 3:**
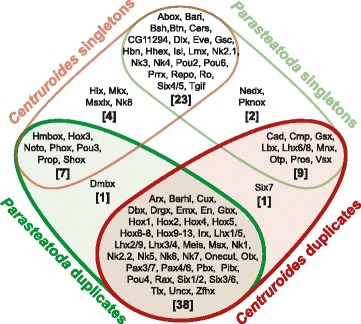
Homeobox-containing genes are frequently duplicated in *P. tepidariorum* and *C. sculpturatus*. Many duplicated homeobox gene families (overlap of red and green shading) are shared between *P. tepidariorum* (indicated in green) and *C. sculpturatus* (indicated in red). Single copy families are the next largest group shared, then families that are single copy in one species but duplicated in the other. There are also a few families that were only found in one species

To better understand the patterns of gene duplication in *P. tepidariorum*, we next investigated the duplication level and colinearity of specific coding and non-coding genes. We identified 80 homeobox gene families in *P. tepidariorum* (Additional file 5: Table S2) of which 58% were duplicated, giving a total of 145 genes (Fig. 3). Note that a very similar repertoire was also observed in *C. sculpturatus*, where 59% of homeobox gene families were duplicated (156 genes representing 82 gene families (Additional file 6: Table S3)). Of the 46 and 48 homeobox gene families with multiple gene copies in *P. tepidariorum* and *C. sculpturatus*, respectively, 38 were common to both species. In addition, 23 families were represented by a single gene in both the spider and scorpion genomes (Fig. 3). The few remaining families contained duplicates in only one of these two species or were only found in one species (Fig. 3). In addition, one family, *Dmbx*, had two copies in *P. tepidariorum* but was missing in *C. sculpturatus*.

The duplication of Hox gene clusters in vertebrates was among the first clues that led to the discovery of ancient WGDs in this group [13]. Therefore, we assessed the repertoire and organization of Hox genes in *P. tepidariorum* in comparison to three other spider genomes (*L. hesperus*, *S. mimosarum*, and *A. geniculata* [49]), two scorpion genomes (*C. sculpturatus* and *M. martensii* [48], this study), and the tick genome (*I. scapularis* [45, 46]).

**Fig. 4:**
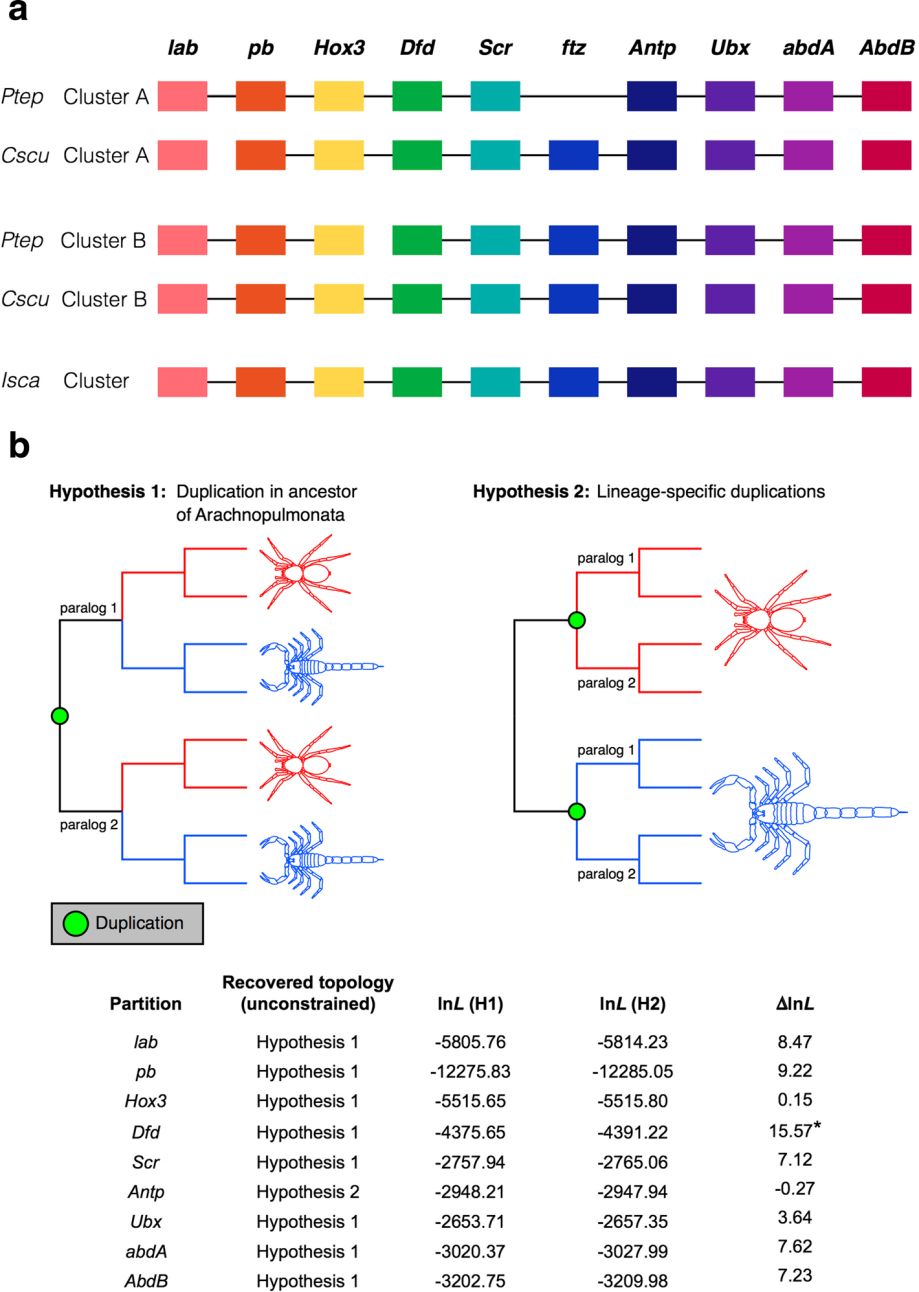
Hox gene complement and hypothetical Hox clusters in chelicerate genomes. Hox gene clusters in the spider *Parasteatoda tepidariorum*, the scorpion *Centruroides sculpturatus*, and in the tick (**a**). For details, see Additional file 9: Table S4. Transcription for all genes is in the reverse direction. Genes (or fragments thereof, see Additional file 9: Table S4) that are found on the same scaffold are joined by black horizontal lines. Abbreviations: *Ptep Parasteatoda tepidariorum*, *Cscu Centruroides sculpturatus*, *Isca Ixodes scapularis*. **b** Gene tree analysis of individual Hox genes support a shared duplication event in the common ancestor of spiders and scorpions in all cases except *Antennapedia*

We identified and manually annotated orthologs of all ten arthropod Hox gene classes (*labial* (*lab*), *proboscipedia* (*pb*), *Hox3*, *Deformed* (*Dfd*), *Sex combs reduced* (*Scr*), *fushi tarazu* (*ftz*), *Antennapedia* (*Antp*), *Ultrabithorax* (*Ubx*), *abdominal-A* (*abdA*), and *Abdominal-B* (*AbdB*)) in all genomes surveyed (Fig. 4, Additional file 7: Figure S4, Additional file 8: Figure S5, Additional file 9: Table S4). Whereas the tick genome contains only one copy of each Hox gene, nearly all Hox genes are found in two copies in the spider and scorpion genomes (Fig. 4, Additional file 8: Figure S5, Additional file 9: Table S4). The only Hox gene not found in duplicate is *ftz* in *P. tepidariorum* (Fig. 4, Additional file 8: Figure S5, Additional file 9: Table S4).

Interestingly, none of the Hox paralogs present in spiders and scorpions were found as tandem duplicates. Instead, in *P. tepidariorum*, the species with the most complete assembly in this genomic region, it was clear that the entire Hox cluster had been duplicated. We found one *P. tepidariorum* Hox cluster copy in a single scaffold, lacking only a *ftz* copy, as is probably the case for this particular cluster (cluster A) in all spiders (Fig. 4, Additional file 8: Figure S5, Additional file 9: Table S4). The second Hox cluster (cluster B) was split between two scaffolds, which could be due to the incomplete assembly of this region due to there not being enough sequence downstream of *Dfd* (~70 kb) and upstream of *Hox3* (~320 kb) to cover the paralogous ~840 kb between *Dfd* and *Hox3* on Cluster A in *P. tepidariorum* or even the ~490 kb between *Dfd* and *Hox3* in *I. scapularis* (Fig. 4, Additional file 8: Figure S5, Additional file 9: Table S4). Note that for clarity and to be consistent with the vertebrate nomenclature, we have named the *P. tepidariorum* Hox paralogs after the cluster that they are found in, for example, *pb-A*, *pb-B*, etc. (Additional file 8: Figure S5, Additional file 9: Table S4).

In addition to the Hox genes, the clusters also contained microRNAs, including a single copy of mir-10 in cluster B. Two copies of microRNAs iab4/8 were identified in both clusters, between *abdA* and *AbdB* (Additional file 8: Figure S5, Additional file 10: Table S5). Furthermore, mir-993b-1 was found in cluster B, but the other two *P. tepidariorum* mir-993 paralogs [44] were located in non-Hox containing scaffolds. In addition to these microRNAs, 98 other putative/predicted coding and non-coding genes were also found in the *P. tepidariorum* Hox clusters (Additional file 8: Figure S5, Additional file 10: Table S5). However, none of these other genes were present as duplicates in both clusters in the same syntenic arrangement.

It was also recently reported that approximately 36% of annotated microRNAs in *P. tepidariorum* are present as two or more copies [44]. Analysis of the synteny of the paralogous *P. tepidariorum* microRNAs shows that only 8 out of 30 are found on the same scaffold. Furthermore, nearly all of the tandemly duplicated microRNAs in *P. tepidariorum* are microRNAs largely specific to this spider (e.g., mir-3971 paralogs) or clustered in arthropods (e.g., mir-2 from the mir-71/mir-2 cluster) (Additional file 11: Table S6) [44]. These findings suggest that the majority of duplicated microRNAs were not generated by tandem duplication.

Comparative analyses suggest that other key developmental genes are also commonly duplicated in *P. tepidariorum.* A synteny analysis of these previously reported duplications showed that only the two *Pax6* paralogs were located on the same scaffold (Additional file 12: Table S7), suggesting that they arose through tandem duplication. The paralogs of other duplicated developmental genes examined were found on different scaffolds (Additional file 12: Table S7), including retinal differentiation (*dachshund* and *sine oculis*), head patterning (*six3*, *orthodenticle*, *collier*) [57, 58], Wnt pathway genes (*Wnt7*, *Wnt11*, *frizzled 4*) [37, 59], and appendage formation genes (*homothorax*, *extradenticle*, *Lim1*, *spineless*, *trachealess*, and *clawless*) (Prpic et al., unpublished data).

Classification of duplicated genes in spiders and scorpions shows that tandem and especially dispersed duplications abound in these genomes. The observation that most of the duplicated genes are found on different scaffolds is suggestive of large-scale duplication, with the caveat that the scaffolds do not represent chromosomes, and therefore the frequency of tandem duplications could be underestimated. Taken together, these results, and the finding that the Hox cluster has also been duplicated, could be indicative of a WGD.

### Conservation of synteny among *P. tepidariorum* scaffolds supports the hypothesis of a WGD event

**Fig. 5:**
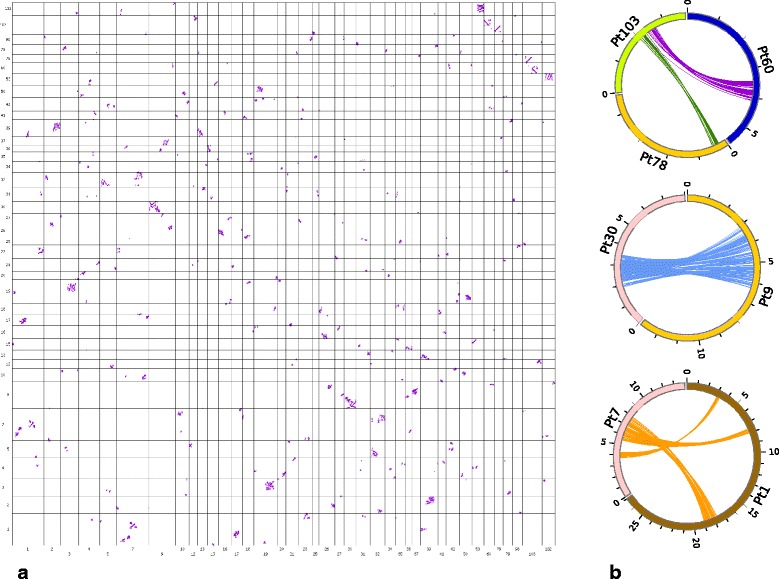
Genome-scale conservation of synteny among *P. tepidariorum* scaffolds reveals signatures of an ancient WGD. **a** Oxford grid displaying the colinearity detected by SatsumaSynteny among the 39 scaffolds presenting the greatest numbers of hits on one another. On this grid (not drawn to scale), each point represents a pair of identical or nearly identical 4096-bp regions. Alignments of points reveal large segmental duplications suggestive of a whole-genome duplication event along with other rearrangements such as inversions, translocations and tandem duplications. **b** Circos close-ups of some of the colinearity relationships revealed by the Oxford grid

To further test the hypothesis that a WGD event had occurred in an ancestor of *P. tepidariorum*, we next searched for conserved synteny among the genomic scaffolds of this spider using Satsuma [60] (note that this approach was not possible in *C. sculpturatus* because of the assembly quality of the genome of this scorpion). This analysis revealed signatures of large segmental duplications suggestive of a WGD followed by numerous rearrangements (inversions, translocations, tandem duplications) (Fig. 5a). These signatures were observed among many of the larger scaffolds (Fig. 5, Additional file 13: Figure S6), but were particularly strong and clear between scaffolds 1 and 7, between scaffolds 9 and 30, and among scaffolds 60, 78, and 103 (Fig. 5b). These results are comparable to findings from a similar analysis of the genome of the fish *Tetraodon nigroviridis* [17] and are consistent with an ancient WGD event in an ancestor of this spider.

### When did WGD occur in chelicerates?

To determine the timing of duplication relative to species divergence within a broader taxonomic sampling of arachnids than analyzed thus far, we grouped the protein-coding genes of 30 arachnid species into gene families with either *P. tepidariorum* or *C. sculpturatus* translated genes used as a seed plus *L. polyphemus* and *S. maritima* as outgroups (Additional file 14: Table S8) [61]. This method resulted in 2734 unique *P. tepidariorum*-seeded gene families (Additional file 15: Figure S7). Note that seeding gene families with *C. sculpturatus* resulted in fewer families (1777) but similar patterns of gene duplication (not shown); we thus focused on the results of *P. tepidariorum*-seeded families.

**Fig. 6:**
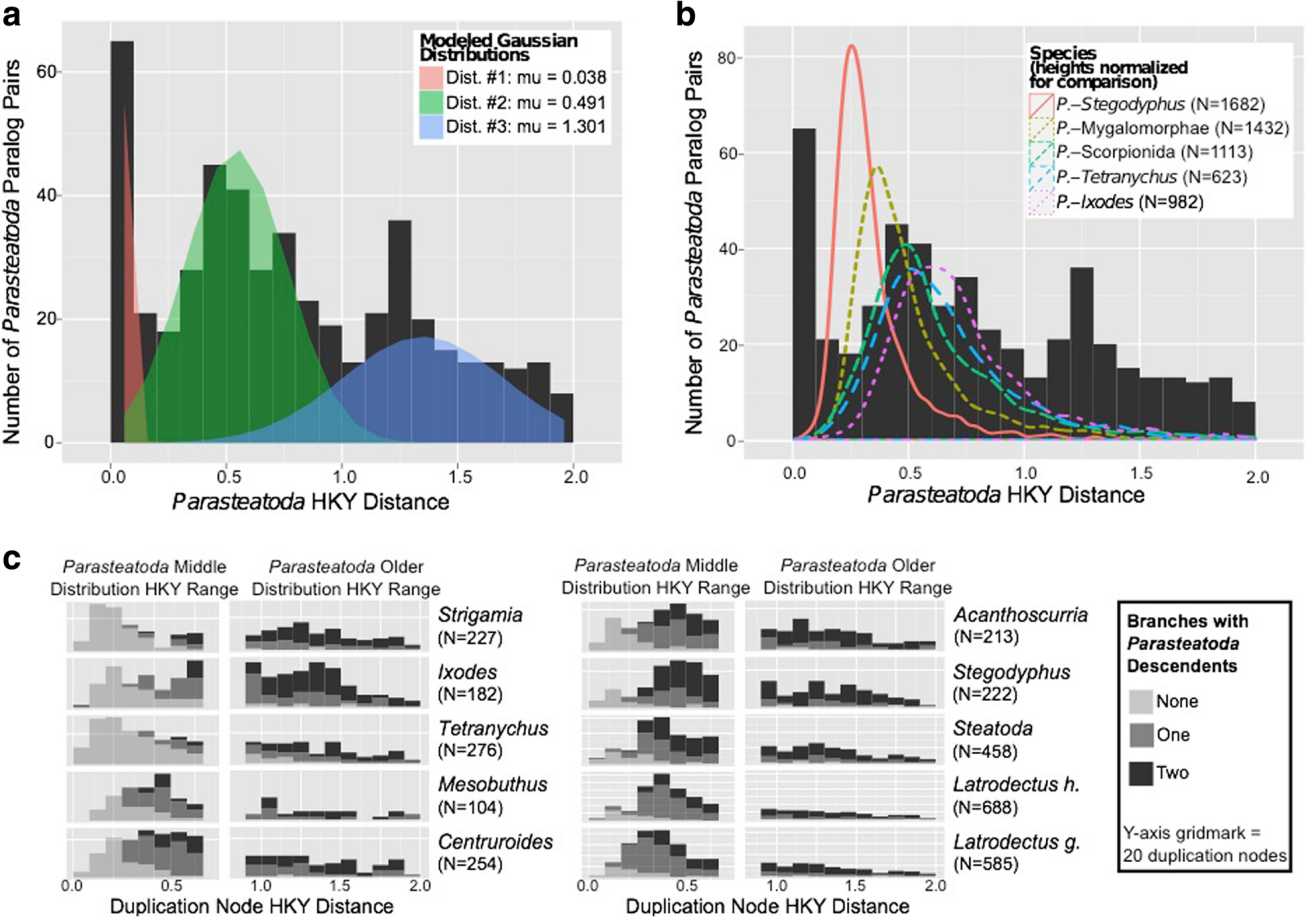
Molecular distance distributions of *P. tepidariorum* paralogs and speciation nodes. The distribution of mean HKY distances from *P. tepidariorum* duplication nodes to *P. tepidariorum* descendants reveals three distributions shown in different colors in (**a**). Comparing the distribution of HKY distances from speciation nodes to *P. tepidariorum* (lines in **b**) reveals that distribution #1 (red in **a**) is restricted to the *P. tepidariorum* branch, distribution #2 (green in **a**) is similar to pre-spider and post-tick speciation nodes, and distribution #3 (blue in **a**) is older than the *P. tepidariorum*-tick speciation event. N = number of speciation nodes in (**b**). Comparing the number of duplication nodes in non-*P. tepidariorum* species (**c**) that are either partially or fully retained in *P. tepidariorum* reveals that the duplication nodes with HYK distances in the range of the oldest *P. tepidariorum* distribution (blue in **a**) are retained at a similar rate across all species (right sub-columns in **c**), but that those duplication nodes with HKY distances in the range of the middle *P. tepidariorum* distribution (green in **a**) are only retained in scorpions or more closely related species (left sub-columns in **c**)

To analyze the timing of the putative WGD event, we calculated molecular distances between paralog pairs by averaging the maximum likelihood branch lengths estimated under the HKY model of evolution [62] within gene trees from the duplication node to all descendant within-species paralogs. We fit the molecular distances of duplication nodes with HKY > 0.01 (avoid inferring alleles as paralogs) and HKY < 2.0 (minimize mutational saturation) to five distribution models. The results show that *P. tepidariorum* duplication nodes best fit three Gaussian distributions (four other distributions were rejected by the Kolmogorov–Smirnoff goodness-of-fit test, see Additional file 16: Table S9). The first Gaussian distribution, with an average genetic distance of μ = 0.038 likely represents recent individual gene duplications. The second (μ = 0.491) and third (μ = 1.301) distributions of genetic distance among paralogs are consistent with two ancient large-scale duplication events (Fig. 6a) [11, 63]. We observed a similar distribution of paralog molecular distances in five deeply sequenced spider species and *C. sculpturatus* (Additional file 17: Figure S8, Additional file 18: Table S10), but not *T. urticae* and *I. scapularis*. The shift in distribution patterns between the scorpion and the mite is consistent with a shared WGD in spiders and scorpions that was not experienced by the more distantly related arachnid species. It is also possible that spiders and scorpions experienced independent duplication events shortly after their divergence, but this is unlikely given the shared retention of paralogs from this analysis (see below) and from the BUSCO-Ar and OMA gene sets (see above).

The possibility that a WGD occurred prior to the divergence of spiders and scorpions and after the divergence of spiders from mites is additionally supported by comparison of the distributions of HKY distances of the duplication nodes to speciation nodes, with an almost identical pattern found for the paralog distances and the spider–scorpion distances (Fig. 6b, Additional file 19: Figure S9, Additional file 20: Table S11). Shared paralog retention is also high for spiders and scorpions, but not between spiders and ticks or mites, further supporting a shared WGD in the spider and scorpion common ancestor (Fig. 6c, Additional file 21: Table S12). Furthermore, the tandem duplication nodes identified above formed the majority of the duplication nodes in the younger Gaussian distribution (71%), and minorities of the second (24%) and third distributions (9%) (Additional file 22: Figure S10). This is the opposite of what is seen with the duplication nodes containing dispersed duplications (younger: 29%, second: 62%, and third: 50%). Additionally, a slight majority of the older tandem duplication nodes showed evidence of being shared with other arachnids (57%), but mostly with other species in the same family as *P. tepidariorum* (44%). This suggests that an ancient WGD was followed by pervasive lineage-specific tandem duplications, especially in spiders.

Analysis of the gene families containing a duplication pair from the middle and oldest Gaussian distributions (Fig. 6a), excluding tandem duplicates, showed that they are enriched in several GO terms compared to gene families without duplication pairs, including several terms associated with transcription and metabolism (Additional file 23: Table S13). The same GO terms are also enriched in these gene families compared to the families with tandem duplications, but the difference is not significant. However, the gene families with tandem duplication pairs are depleted in GO terms relating to translation.

### Gene trees support the common duplication of genes in Arachnopulmonata

**Fig. 7:**
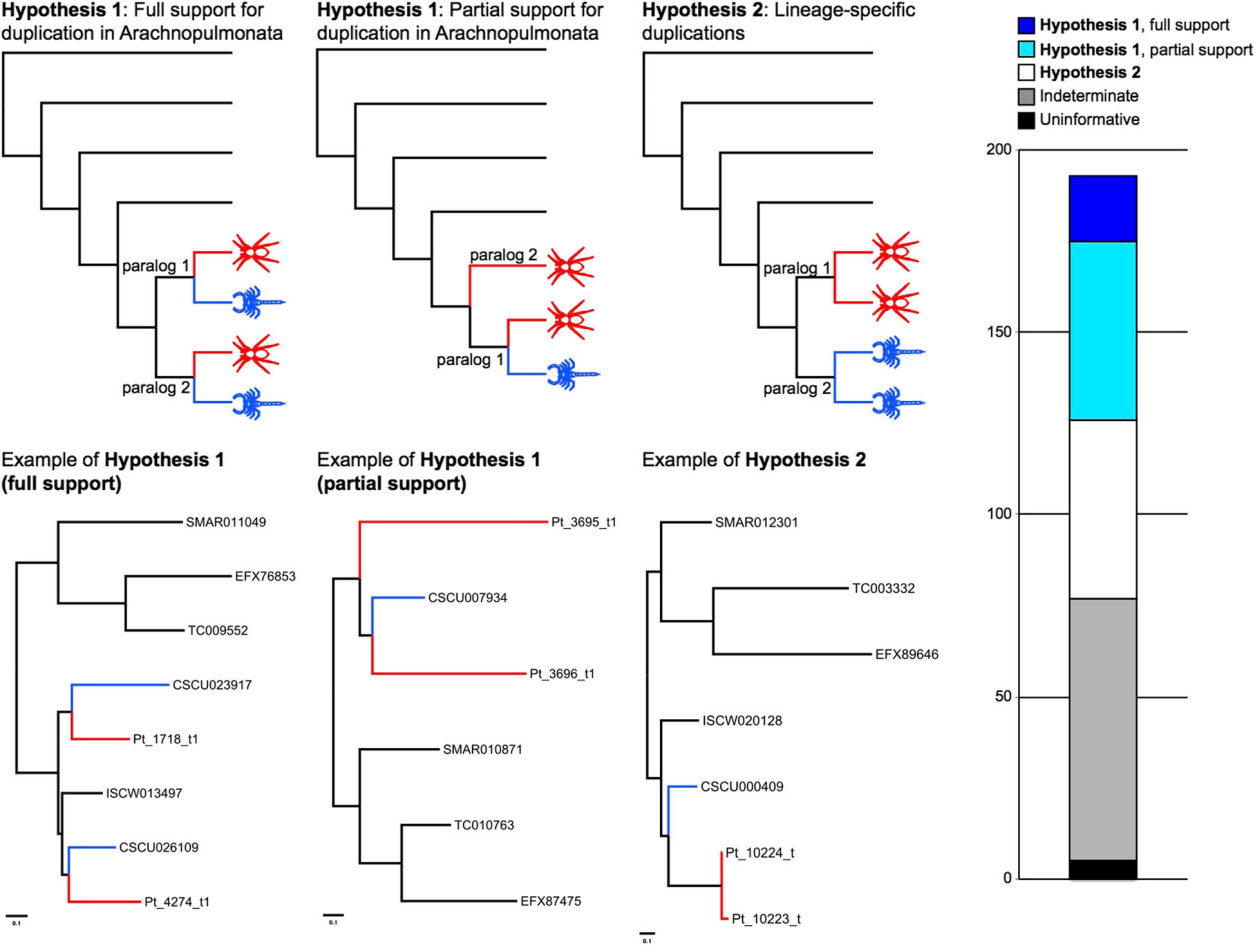
Gene trees support the common duplication of genes in Arachnopulmonata. Analysis of gene trees inferred from six arthropod genomes was conducted, with the gene trees binned by topology. Trees corresponding to a shared duplication event were binned as Hypothesis 1, and trees corresponding to lineage-specific duplication events as Hypothesis 2. Gene trees with spider paralogs forming a clade with respect to a single scorpion paralog were treated as partially consistent with Hypothesis 1. Top row of panels shows hypothetical tree topologies; bottom row of panels shows empirical examples. Right panel shows distribution of gene trees as a function of bin frequency

The results of our analysis of duplicated genes in *P. tepidariorum* and other arachnids from the OMA and BUSCO gene sets, as well as our dating of the divergence in gene families, strongly suggest that there was a WGD in the ancestor of spiders and scorpions. To further explore whether the duplicated genes in spiders and scorpions were the result of duplication in the most recent common ancestor of these arachnopulmonates (Hypothesis 1) or lineage-specific duplications (Hypothesis 2), we applied a phylogenetic approach to examine *P. tepidariorum* and *C. sculpturatus* genes (Fig. 7, Additional file 24: Table S14, Additional file 25: Table S15). Of the 116 informative gene trees (see Methods) of orthogroups, wherein exactly two *P. tepidariorum* paralogs were present for a single *T. castaneum* ortholog, 67 (58%; henceforth Tree Set 1) were consistent with a common duplication (Hypothesis 1) and 49 (42%) were consistent with lineage specific duplications (Hypothesis 2) (Fig. 7, Additional file 24: Table S14, Additional file 25: Table S15). Of the 67 tree topologies supporting a common duplication, 18 were fully congruent with the idealized Hypothesis 1 tree topology and 49 were partially congruent with Hypothesis 1 (i.e., the two spider paralogs formed a clade with respect to a single scorpion ortholog) (Fig. 7, Additional file 24: Table S14, Additional file 25: Table S15).

If the gene trees in Tree Set 1 were the result of large-scale duplication events or WGD as opposed to tandem duplication, we would expect each resulting copy to occupy two different scaffolds. Of the 18 *P. tepidariorum* paralog pairs from gene trees fully consistent with Hypothesis 1, 15 were found to occupy different *P. tepidariorum* scaffolds; of the 49 paralog pairs from gene trees partially congruent with Hypothesis 1, all but ten pairs were found to occupy different *P. tepidariorum* scaffolds (Additional file 26: Table S16). In addition, of the 18 *C. sculpturatus* paralog pairs that were fully consistent with Hypothesis 1, all 18 were found on different scaffolds. To test whether *P. tepidariorum* paralog pairs located on different scaffolds compared to the three paralog pairs found on the same scaffolds was simply a consequence of differences in assembly quality, we examined the length of the scaffolds for these two groups. We found the lengths of the scaffolds were statistically indistinguishable between the two groups (Additional file 26: Table S16; Wilcoxon rank sum test: W = 358, *P* = 0.9179). This analysis was not required for the 18 scorpion paralog pairs because, in all cases, each member of the scorpion paralog pair was distributed on a different scaffold.

The occurrence of two clusters of Hox genes in both the spider and scorpion genomes could also be consistent with either of these alternative hypotheses (Fig. 4b). However, only in the case of *Antp* was a tree topology consistent with Hypothesis 2 recovered and the difference in log likelihood between the two hypotheses was negligible (ln*L* = –0.27) (Fig. 4b). Higher statistical support for the Hypothesis 1 topology was generally obtained for data partitions with a large number of available sequences (e.g., *Dfd*, *pb*) (Fig. 4b). The sum of the Hox gene tree data is therefore consistent with the synteny analysis, and supports a shared duplication in the common ancestor of Arachnopulmonata.

### WGD in Xiphosura is probably unrelated to the duplication of genes in Arachnopulmonata

**Fig. 8:**
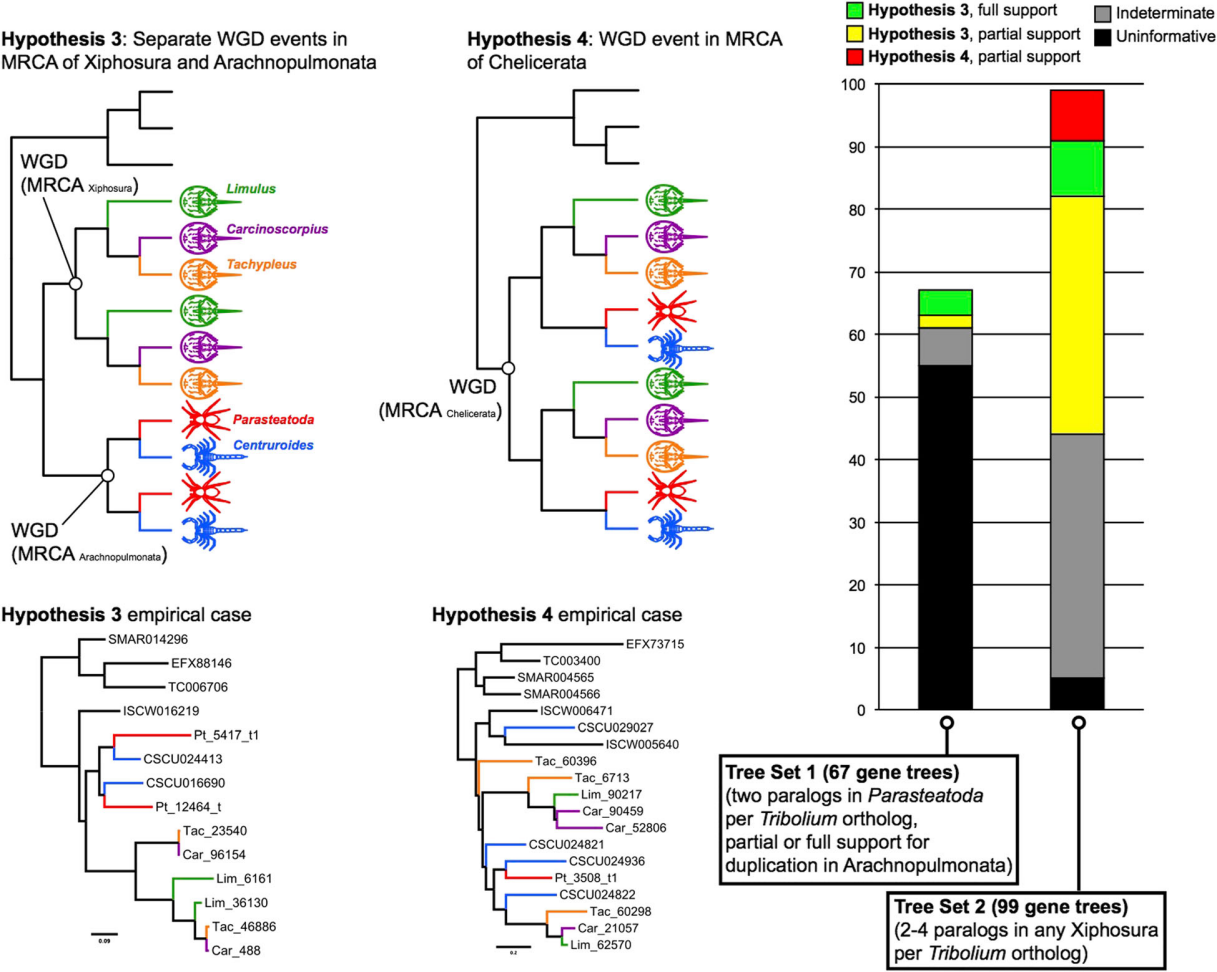
WGD in Xiphosura is probably unrelated to the duplication of genes in Arachnopulmonata. Analysis of gene trees inferred from nine arthropod genomes was conducted, with the gene trees binned by topology. Trees corresponding to two separate duplication events in the most recent common ancestor (MRCA) of Xiphosura and Arachnopulmonata were binned as Hypothesis 3, and trees corresponding to a single duplication event in the MRCA of Chelicerata as Hypothesis 4. Top row of panels shows hypothetical tree topologies; bottom row of panels shows empirical examples. Right panel shows distribution of gene trees as a function of bin frequency, for two different tree sets (i.e., gene trees retrieved under two alternate filtering criteria). Note the limited support for Hypothesis 4, with empirical gene trees poorly matching the expected tree topology (contra empirical cases supporting Hypothesis 3)

The recent report of WGD and multiple Hox clusters in an analysis of horseshoe crabs (Order Xiphosura [31]) raises the possibility of two alternative interpretations, namely (1) a single WGD at the base of Chelicerata, with losses of duplicated genes in lineages like mites and ticks, or (2) separate WGD events in the horseshoe crab ancestor and in the arachnopulmonate ancestor. To discern whether the WGD event(s) recently reported in Xiphosura constitute separate (Hypothesis 3) or common (Hypothesis 4) evolutionary events from the duplication of genes in Arachnopulmonata, we added the three published horseshoe crab genomes to our dataset and reran OMA (Fig. 8). If the duplications reported here in spiders and scorpions were caused by the same event that drove the genome duplications in horseshoe crabs, we would expect to find paralog clusters that included members of all Euchelicerata (Xiphosura + Arachnida). This expected pattern is comparable to the case of whole genome duplications in the vertebrate ancestor [30], which resulted in the same sets of paralogs for all major vertebrate lineages, to the exclusion of non-vertebrate deuterostomes and the protostomes (e.g., the *Sp* gene family [64]). By contrast, if the duplications in spiders and scorpions were distinct from the duplications in horseshoe crabs, we would expect to observe a pattern where (1) horseshoe crab paralogs clustered together, (2) arachnopulmonate paralogs clustered together, and (3) all other arachnid orthologs would not be duplicated at all and fell somewhere in between horseshoe crabs and arachnopulmonates (Fig. 1) [53]. We thus examined gene trees recovered by OMA to discern which of these two scenarios was supported by the comparison of the nine full genomes.

We first examined the orthogroups corresponding to Tree Set 1, after addition of horseshoe crab orthologs (Fig. 8). However, we found that 55 of the 67 gene trees constituting Tree Set 1 could not distinguish between Hypothesis 3 and Hypothesis 4 (i.e., no horseshoe crab paralogs were recovered in those orthogroups with duplicated spider genes).

We assembled a second tree set (henceforth, Tree Set 2) using the filtering criterion of orthogroups where 2–4 xiphosuran paralogs were recovered for a single *T. castaneum* ortholog. We thus recovered 99 gene trees in Tree Set 2 (Fig. 8). Of these, 44 were indeterminate (non-monophyletic outgroup) or uninformative (either missing all arachnopulmonates or missing all xiphosuran paralogs). A further 47 were consistent with Hypothesis 3, with nine gene trees completely congruent with Hypothesis 3 (i.e., multiple paralog clusters within both arachnopulmonates and horseshoe crabs, monophyly of Arachnopulmonata and Xiphosura, and monophyly of the mandibulate outgroup) (Fig. 8). The last eight gene trees in Tree Set 2 were scored as partially consistent with Hypothesis 4, but as shown in one empirical case (Fig. 8), these gene trees did not correspond well to the scenario of a common WGD at the base of Chelicerata, and may stem from algorithmic error in phylogenetic reconstruction (e.g., model misspecification). To be conservative, we treated these eight trees as consistent with our alternative hypothesis.

The sum of our gene tree analyses thus indicates support for Hypothesis 3 – the independent origins of arachnopulmonate and xiphosuran duplications. We found very little support for a shared duplication event at the base of Chelicerata (Hypothesis 4); no gene tree could be found where multiple paralogous groups each included exemplars of Xiphosura and Arachnopulmonata. Taken together, these results suggest that the duplication of genes in spiders and scorpions was probably independent of the proposed WGD events in horseshoe crabs.

### Hox gene paralogs in *P. tepidariorum* show considerable divergence in temporal and spatial expression during embryogenesis

**Fig. 9:**
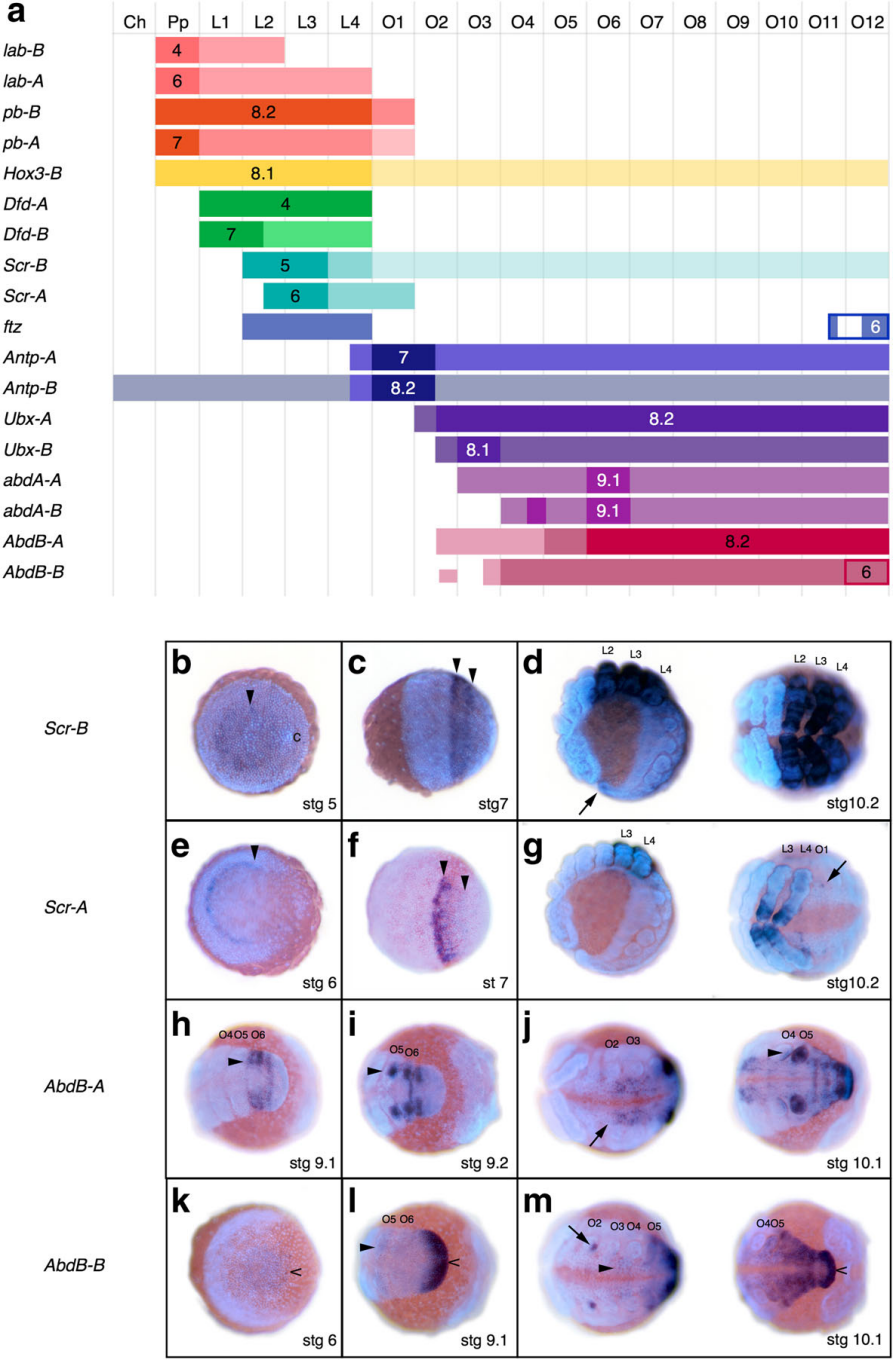
Expression of Hox paralogs in *P. tepidariorum*. **a** Summary of Hox gene expression domains and expression timing in *P. tepidariorum* embryos. Columns represent segments from anterior to posterior. Bars represent the extent of a gene’s expression domain with respect to the segments. The darkest color for each gene is used for the initial expression domain of each gene when it first appears, which usually coincides with a genes’ strongest expression. The next lighter color is used for the expanded domain, and the lightest color is used for further late expansions of the expression domains, which usually tends to be only in the nervous system. The stage at which a gene’s expression first appears is depicted by the stage number in the domain of first expression. *ftz*, in addition to its Hox domain, is expressed dynamically (i.e., budding off stripes) in the SAZ, and *AbdB-B* is continuously expressed in the SAZ after its formation at stage 6. These SAZ expression patterns are indicated by rectangular outlines in what is otherwise the O12 segment. Note that, since we did not detect clear expression boundaries for *Hox3-A*, the expression of this gene is not represented. **b**–**m** Two examples of Hox gene expression differences between paralogs of *Scr* (**b**–**g**) and *AbdB* (**h**–**m**). For detailed descriptions of expression patterns, see Additional file 44: Supplementary File 1 and the legends of Additional file 33: Figure S17, Additional file 34: Figure S18, Additional file 42: Figure S26, Additional file 43: Figure S27. All images are overlays of a bright-field images depicting the expression pattern and a fluorescent DAPI nuclear staining. Abbreviations: *Ch* cheliceral segment, *Pp* Pedipalpal segment, *L–L4* walking leg segments 1–4, *O1–12* opisthosomal segments 1–12

Alteration of the temporal and/or spatial expression can underlie the neo- or sub-functionalization of duplicated genes. To test whether the Hox gene paralogs in chelicerates have divergent expression patterns, we assayed the expression of all Hox genes throughout *P. tepidariorum* embryogenesis (for *lab-A* and *lab-B* expression see [65, 66]). For each pair of Hox paralogs, we found remarkable differences in spatial and temporal expression patterns (Fig. 9, Additional file 27: Figure S11, Additional file 28: Figure S12, Additional file 29: Figure S13, Additional file 30: Figure S14, Additional file 31: Figure S15, Additional file 32: Figure S16, Additional file 33: Figure S17, Additional file 34: Figure S18, Additional file 35: Figure S19, Additional file 36: Figure S20, Additional file 37: Figure S21, Additional file 38: Figure S22, Additional file 39: Figure S23, Additional file 40: Figure S24, Additional file 41: Figure S25, Additional file 42: Figure S26, Additional file 43: Figure S27, Additional file 44: Supplementary File1).

The expression of the paralogs of each Hox gene never appears at the same time during development; the expression of one paralog often precedes the other by at least 10 hours (e.g., *lab*, *Scr*, *Ubx*, and *abdA*) [65, 66] (Fig. 9b–g), if not 15 to 20 hours (*pb*, *Dfd*, *Antp*), or even 30 hours as in the case of *AbdB* (Fig. 9a, h–m). The expression domains of paralogs also differ significantly in their anterior and/or posterior borders. *Scr*, *Ubx*, *abdA*, and *AbdB* paralogs exhibit anterior borders that are shifted by half a segment or more, and several Hox gene paralogs expressed in the prosoma show shifts in their posterior expression borders by one or more segments (Fig. 9a). While the borders of the strongest expression domain are identical in the case of the paralogs of *lab*, *Antp*, and *abdA*, they differ substantially in all other paralogs (Fig. 9, Additional file 27: Figure S11, Additional file 28: Figure S12, Additional file 29: Figure S13, Additional file 30: Figure S14, Additional file 31: Figure S15, Additional file 32: Figure S16, Additional file 33: Figure S17, Additional file 34: Figure S18, Additional file 35: Figure S19, Additional file 36: Figure S20, Additional file 37: Figure S21, Additional file 38: Figure S22, Additional file 39: Figure S23, Additional file 40: Figure S24, Additional file 41: Figure S25, Additional file 42: Figure S26, Additional file 43: Figure S27), but note that the expression boundaries detected for *Hox3-A* were somewhat unclear (Additional file 29: Figure S13).

Most Hox gene paralogs also exhibit differences in the tissues and cell types they are expressed in (e.g., mesodermal vs. ectodermal expression, or groups of neuroectodermal cells that a paralog is expressed in), which hints at the possible neo-functionalization of one of the paralogs. For example, in the case of the *AbdB* paralogs (Fig. 9h–m), only *AbdB-B*, is expressed in the segment addition zone where it has a dynamic anterior expression border until a more Hox-like expression domain appears at stage 9.

While most Hox gene paralogs in *P. tepidariorum* follow spatial colinearity rules, i.e., genes at the beginning of the Hox cluster are expressed more anteriorly than genes at the end of the Hox cluster, a few Hox genes in *P. tepidariorum* do not adhere to these rules (Fig. 9a). Except for *AbdB-B*, all of the earliest expression domains are strictly spatially colinear; however, later during development, expression domains of a few genes extend beyond the expected spatial domains (*ftz*, *Antp-A*, *AbdB-A*, and *-B*).

Temporal colinearity rules, however, are not always followed by *P. tepidariorum* Hox genes. While genes at the beginning of the clusters are generally expressed earlier than the ones at the end of the clusters, there are many genes that do not adhere to temporal colinearity rules. Additionally, there is no temporal colinearity of expression initiation within either cluster A or B.

Taken together, we have observed considerable differences in the spatial and temporal expression between each of the *P. tepidariorum* Hox gene paralogs (Fig. 9). These differences likely reflect changes in function between the paralogs that have evolved in the time since the cluster was duplicated.

## Discussion

### Signatures of an ancient WGD in the last common ancestor of spiders and scorpions

Our study of the assembly and annotation of the *P. tepidariorum* genome revealed a high number of duplicated genes in accordance with previous observations [34–44]. This finding is further supported by our detection of a colinearity signal across many of the largest *P. tepidariorum* scaffolds. The fact that we find many smaller synteny blocks across scaffolds suggests that the WGD event occurred early during spider evolution and was followed by extensive disruption of previously larger blocks, for instance, by recombination or the activity of transposable elements. Intriguingly, the comparison of the gene content of the *P. tepidariorum* genome with other chelicerates and other arthropods suggests that a WGD likely occurred in the lineage leading to spiders and scorpions. Our dating efforts indeed confirmed that this WGD most likely occurred after the divergence of the common ancestor of spiders and scorpions from other arachnid lineages (mites, ticks, and harvestmen) prior to 430 MYA [67, 68] (Fig. 1). Furthermore, our results suggest that this event was independent of the apparent WGDs shared by all extant horseshoe crabs [31].

### Divergence in gene function after duplication

It is thought that typically large-scale duplication events such as WGD are followed by a period of gene loss (for example, only 12% of paralogs have been retained after 100 MY in *Saccharomyces cerevisiae* [7, 23]), in concert with major genomic rearrangements, and that those duplicated genes that are subsequently retained are enriched in developmental genes such as those encoding transcription factors and other proteins that often act in multiprotein complexes [2, 18, 24, 25, 69]. Our GO term enrichment analysis partially confirms a similar trend for *P. tepidariorum*, since we find, for instance, proteins related to transcriptional regulation enriched in the group of duplicates. Indeed, it is striking that vertebrates, horseshoe crabs, and arachnopulmonates have retained duplicated Hox clusters and appear to be enriched in other paralogs that encode other transcription factors, suggesting that this retention pattern after WGDs is a general trend in animals.

Our study provides evidence for possible subsequent sub-functionalization and neo-functionalization among ohnologs [19–22, 69], most likely as a result of evolutionary changes in their regulatory sequences as has been observed in the case of other WGD events [70]. This is exemplified by the diversity in the temporal and spatial expression of the *P. tepidariorum* Hox gene paralogs during embryogenesis (e.g., Fig. 9). Divergence in the expression patterns of duplicated Hox genes has been previously reported for the genes *Dfd*, *Scr*, and *Ubx* in spiders [38, 71, 72] and for the posterior Hox genes *Antp*, *Ubx*, *abdA*, and *AbdB* in the scorpion *C. sculpturatus* [40]. However, these previous studies only investigated a few Hox gene families and analysis of the spatial expression of these genes was limited to later developmental stages after the appearance of limb buds. Divergence in gene expression has also been previously observed for duplicated Wnt ligand genes in *P. tepidariorum* [37]. In addition, a recent study of the two *dachshund* paralogs provided possible evidence for the neo-functionalization of a duplicated gene during the evolution of a morphological novelty in spiders [41].

### Gene duplication and arachnid evolution

Our findings have profound implications for the evolution of chelicerates as a whole, a group whose internal phylogeny has proven extremely difficult to resolve [53]. Focal to understanding the evolution of terrestrialization in this group are the relationships of five arachnid orders possessing book lungs. The close relationship of four of these groups, namely spiders, amblypygids, thelyphonids, and schizomids, is generally not contested and both morphological and molecular trees place them together in a monophyletic clade, the Tetrapulmonata. The position of scorpions in the chelicerate tree, however, is much more controversial. It has been argued that their terrestrial adaptations, including the book lungs, evolved convergently to those of tetrapulmonates, whereas recent phylogenomic analyses have placed scorpions (possibly a sister group to Pseudoscorpiones) as the sister group to Tetrapulmonata [53, 73]. The shared paleopolyploidization event between spiders and scorpions provides further evidence that these two groups are more closely related to each other than they are to other apulmonate and non-duplicated arachnids (e.g., mites and ticks), which is in agreement with recent molecular phylogenies. This would imply a single origin of the arachnid book lungs as has been suggested previously based on detailed ultrastructural morphological analyses [74], raising the possibility that the ancient WGD identified here can be tested using new comparative genomic data and sampling such lineages as amblypygids, thelyphonids, and schizomids.

The age of the duplication event identified here must predate the most recent common ancestor of spiders and scorpions. Molecular clock approaches vary widely on the age of arachnids, and have suggested that arachnids diversified in the Ordovician [75, 76] or in the Silurian [77], with large confidence intervals on node age estimates that often span entire geological periods. However, the earliest stem-group spiders (the extinct order Uraraneida) date to the mid-Devonian (386 MYA [78]), whereas discoveries of Paleozoic scorpions have extended the stratigraphic range of scorpions into the Silurian (430 MYA [67]). The arachnid fossil record thus suggests the mid-Silurian is a conservative floor age of the duplication event. A Paleozoic age of the duplication event at the base of Arachnopulmonata would make this event approximately contemporaneous with the two-fold WGD in the ancestral vertebrate [30].

This reconstruction is consistent with the observation that few genes retain the ancient signal of shared duplication in both arachnopulmonates and vertebrates, and those that do often tend to be developmental patterning genes. For example, when compared to the *Drosophila melanogaster* genome, less than 5% of homologous vertebrate genes retain the 1:4 ortholog ratio expected from the vertebrate two-fold WGD event [30]. However, included among this minority are vertebrate orthologs of Hox genes, whose duplicates have been retained and deployed for various aspects of embryonic patterning. Thus, the patterns observed in arachnopulmonate arachnids are broadly consistent with counterparts in vertebrates.

Currently, it is not possible to address the question of whether the arachnopulmonate WGD facilitated the evolution of a terrestrial life-style and the development of book lungs. Taking advantage of the annotated spider genome sequences and the practical merits of *P. tepidariorum*, however, future functional studies in spiders could analyze paralog sub- and neo-functionalization and gene regulatory network rewiring after duplication to clarify these questions.

## Conclusions

Much has been speculated about the long-term evolutionary consequences of genome duplications, including long-standing discussions on the evolution and origin of our own lineage, the vertebrates, and the complex body plan and diverse ecological adaptations that are hallmarks of this animal group [1, 2, 79–81]. However, it has been argued that there does not appear to be an association between genome duplication and teleost diversification [82]. Furthermore, other groups that have experienced WGD, such as horseshoe crabs and bdelloid rotifers, did not exhibit any apparent diversification or obvious increase in complexity following WGD, with the caveat that there might be changes in the complexity of their physiology, behavior and life history. This suggests that a putative link between WGD and increased diversification, as suggested in vertebrates, may not be generalizable to other taxa [11, 14, 32, 33].

To help address the contribution of WGD to animal diversification, analyzing the outcomes of those independent “experiments” that have naturally occurred during evolutionary time is of paramount importance. Recurrent and independent cases of paleopolyploidization should be studied systematically to reveal commonalities of evolutionary forces experienced across disparate lineages. Our discovery of an ancient genome duplication event preceding the origin of spiders and scorpions helps to fill a crucial gap in the comparative studies of WGDs. Previously reported cases of paleopolyploid lineages in different eukaryotes, including both unicellular and multicellular taxa, only allowed an extremely reduced set of core orthologous genes to be compared across lineages. However, the biology of vertebrates and arachnopulmonates is in many respects very similar, sharing the gene toolkit common to most animal species, highly conserved developmental pathways and even the general layout of the basic bilaterian body plan.

Thus, our results will open new research avenues, allowing the formulation of specific hypotheses about the impact of WGDs on developmental gene regulatory networks and morphological diversity by making direct comparisons and extrapolations with the vertebrate case. Moreover, since *P. tepidariorum* is arguably the primary chelicerate model system in the field of evolutionary development biology [51, 83–85], its genome sequence will provide an excellent resource to functionally test hypotheses based on genomic inferences.

## Methods

### Extraction of genomic DNA

Genomic DNA was extracted from four adult females and eight adult males of a genetically homogenous *P. tepidariorum* strain that was inbred for 15 generations and originally collected in Göttingen. All 12 animals were separated from the general stock before their final molt (to ensure that all specimens were virgin and did not contain genetic material from mating partners or developing embryos), and were starved for 2 weeks prior to DNA extraction (to minimize contamination from gut contents). Directly before DNA extraction, all animals were microscopically inspected to ensure they were free of external parasites (e.g., mites) and were macerated and digested in 80 mM EDTA (pH = 8.0), 100 mM Tris-HCl (pH = 8.0), 0.5% SDS, and 100 μg/mL proteinase K at 60 °C for 2 hours. Genomic DNA was isolated from this solution by salt-chloroform extraction, precipitated with ammonium acetate and ethanol, and dissolved in water. RNA contamination was removed with RNaseA. Purified genomic DNA was precipitated with sodium acetate, washed with ethanol, and dissolved in TE buffer (10 mM Tris-HCl (pH = 7.4), 1 mM EDTA) (pH = 8.0)).

For the bark scorpion *C. sculpturatus*, genomic DNA was extracted from four legs, a pedipalp patella and femur, and the fourth metasomal segment of an adult wild-caught female specimen (Tucson, Arizona, USA). Extraction was performed using the Animal Blood and Tissue protocol for a Qiagen DNeasy kit, with the addition of 16 μL of RNase A (25 mg/mL). Whole body RNA was extracted from the same adult female, an adult male, and a juvenile using one leg, the telson, the fifth metasomal segment, 1/3 of the abdomen (to avoid gut contamination), 1/2 of the cephalothorax, and a pedipalp patella. Total RNA was extracted using Trizol with the addition of glycogen.

### Genome sequencing and assembly

The house spider and bark scorpion are two of 30 arthropod species sequenced as part of the pilot project for the i5K 5000 arthropod genomes project at the Baylor College of Medicine Human Genome Sequencing Center. For all of these species, an enhanced Illumina-ALLPATHS-LG sequencing and assembly strategy enabled multiple species to be approached in parallel at reduced costs. For the house spider, we sequenced five libraries of nominal insert sizes 180 bp, 500 bp, 2 kb, 3 kb, and 8 kb at genome coverages of 39.2x, 35.1x, 19.7x, 49.3x, and 19.3x, respectively (assuming a 1.5 Gb genome size [86]). These raw sequences have been deposited in the NCBI SRA: BioSample ID SAMN01932302. For the bark scorpion, we sequenced four libraries of nominal insert sizes 180 bp, 500 bp, 3 kb, and 8 kb at genome coverages of 102.1x, 25.6x, 35.2x, and 39.0x, respectively (assuming a 900 Mb genome size). These raw sequences have been deposited in the NCBI SRA: BioSample SAMN02617800.

To prepare the 180 bp and 500 bp libraries, we used a gel-cut paired-end library protocol. Briefly, 1 μg of the DNA was sheared using a Covaris S-2 system (Covaris, Inc. Woburn, MA) using the 180 bp or 500 bp program. Sheared DNA fragments were purified with Agencourt AMPure XP beads, end-repaired, dA-tailed, and ligated to Illumina universal adapters. After adapter ligation, DNA fragments were further size-selected on an agarose gel and PCR-amplified for 6 to 8 cycles using the Illumina P1 and Index primer pair and Phusion® High-Fidelity PCR Master Mix (New England Biolabs). The final library was purified using Agencourt AMPure XP beads and quality-assessed by Agilent Bioanalyzer 2100 (DNA 7500 kit) to determine library quantity and fragment size distribution before sequencing.

Long mate pair libraries with 2 kb, 3 kb, and 8 kb insert sizes were constructed according to the manufacturer’s protocol (Mate Pair Library v2 Sample Preparation Guide art # 15001464 Rev. A PILOT RELEASE). Briefly, 5 μg (for 2 and 3 kb gap size libraries) or 10 μg (8–10 kb gap size library) of genomic DNA was sheared to the desired size fragments by Hydroshear (Digilab, Marlborough, MA), then end-repaired and biotinylated. Fragment sizes between 1.8 and 2.5 kb (2 kb), 3 and 3.7 kb (3 kb), or 8 and 10 kb (8 kb) were purified from 1% low-melting agarose gel and then circularized by blunt-end ligation. These size-selected circular DNA fragments were then sheared to 400 bp (Covaris S-2), purified using Dynabeads M-280 Streptavidin Magnetic Beads, end-repaired, dA-tailed, and ligated to Illumina PE-sequencing adapters. DNA fragments with adapter molecules on both ends were amplified for 12 to 15 cycles with Illumina P1 and Index primers. Amplified DNA fragments were purified with Agencourt AMPure XP beads. Quantification and size distribution of the final library was determined before sequencing as described above.

Sequencing was performed using Illumina HiSeq2000 generating 100 bp paired-end reads. Reads were assembled using ALLPATHS-LG (v35218) [87] and further scaffolded and gap-filled using Atlas-Link (v.1.0) and Atlas gap-fill (v.2.2) [88]. For *P. tepidariorum*, this yielded an assembly size of 1443.9 Mb with 263,833 contigs with an N50 of 10.1 kb and, after scaffolding and gap closing, 31,445 scaffolds with an N50 of 465.5 kb. Approximately 2416 million reads (96.9x sequence coverage) are represented in this assembly of the *P. tepidariorum* genome. The assembly has been deposited in the NCBI: BioProject PRJNA167405 (Accession: AOMJ00000000).

For the *C. sculpturatus* this yielded an assembly size of 926.4 Mb with 214,941 contigs with an N50 of 5.1 kb and, after scaffolding and gap closing, 10,457 scaffolds with an N50 of 342.5 kb. The final assembly has been deposited in the NCBI: BioProject PRJNA168116.

### Dovetail assembly

#### Chicago library preparation

To further improve the *P. tepidariorum* assembly we used in vitro contact genomics [89] based on the Chicago method (Dovetail Genomics, Santa Cruz, CA) [90]. A Chicago library was prepared as described previously [90]. Briefly, ≥ 0.5 μg of high molecular weight genomic DNA of ≥ 50 kb mean fragment size was extracted from a female *P. tepidariorum*, reconstituted into chromatin in vitro, and fixed with formaldehyde. Fixed chromatin was then digested with *Mbo*I or *Dpn*II, the 5′ overhangs were filled in with biotinylated nucleotides, and the free blunt ends were then ligated. After ligation, crosslinks were reversed and the DNA was purified from protein. Purified DNA was treated to remove all biotin that was not internal to ligated fragments. The DNA was sheared to a mean fragment size of ~350 bp, and sequencing libraries were generated using NEBNext Ultra enzymes and Illumina-compatible adapters. Biotin-containing fragments were then isolated using streptavidin beads before PCR enrichment of the library.

#### Scaffolding the draft genome with HiRise

The *P. tepidariorum* draft genome in FASTA format (1443.9 Mb with a scaffold N50 of 465.5 kb), the shotgun sequences (from approximately 2416 million Illumina reads (see above)), and the Chicago library sequence (187 million read pairs from Illumina HiSeq 2500 2X100bp rapid run) in FASTQ format were used as input data for HiRise, a software pipeline designed specifically for using Chicago library sequence data to assemble genomes [90]. Shotgun and Chicago library sequences were aligned to the draft input assembly using a modified SNAP read mapper [91]. The separations of Chicago read pairs mapped within draft scaffolds were analyzed by HiRise to produce a likelihood model, and the resulting likelihood model was used to identify putative misjoins and score prospective joins. After scaffolding, shotgun sequences were used to close gaps between contigs. This resulted in 16,542 super-scaffolds with an N50 of 4050 kb.

### Genome annotation

#### P. tepidariorum

The *P. tepidariorum* genome assembly (pre-Dovetail) was annotated using version 2.7 of AUGUSTUS [92]. AUGUSTUS constructs genes from evidence such as the RNA-Seq alignments – here called hints – but also uses statistical models for ab initio prediction. The parameters for the statistical models of *P. tepidariorum* genes were estimated on a training set of gene structures. Several steps of parameter estimation, prediction, visual quality control on a genome browser, and parameter tuning were performed.

*P. tepidariorum* transcript alignments were generated using available RNA-Seq libraries [86], namely 1,040,005 reads from 454-sequencing of *P. tepidariorum* embryonic stages, two RNA-Seq libraries from Illumina-sequencing of embryonic stages (333,435,949 and 602,430 reads), and two RNA-Seq libraries from Illumina-sequencing of post-embryonic stages (294,120,194 read and 317,853 reads). In addition, we downloaded all *P. tepidariorum* ESTs [93] and protein sequences available in GenBank. The assembly was repeat-masked using RepeatMasker (version 1.295) [94] and TandemRepeatFinder (version 4.07b) [95] based on a de novo repeat library compiled with RepeatScout (version 1.0.5) [96]; 46% of the bases were masked as repeats.

*P. tepidariorum*-specific parameters of AUGUSTUS were estimated iteratively. An initial training set of genes was generated with PASA (release 2012-06-25) [97] using the ESTs only. This yielded 851 genes that were used to estimate the first set of parameters of AUGUSTUS for the coding regions of genes. Additionally, eukaryotic core proteins were predicted in the masked assembly with CEGMA (version 2.4.010312) [98] and yielded 103 hints for CDS to AUGUSTUS, which were then used in the training stage predictions. With these initial parameters and integrating the evidence from transcriptome data, AUGUSTUS was used to annotate the masked assembly genome-wide. We then extracted another training gene set from the genome-wide prediction by mapping RNA-Seq reads from 454- and Illumina sequencing against predicted transcripts using GSNAP (version 2013-06-27) [99]; however, (1) only genes with 100% RNA-Seq alignment coverage were taken and (2) we mapped the proteins from the database UniRef50 (version UniProt Release 2013 06) [100] against predicted proteins using BLASTP (version 2.2.25) [101], keeping only fully covered transcripts. The genes in the intersection of both sets – that is, genes fulfilling constraints (1) and (2) simultaneously – were used for a second iteration of parameter training. The UTR parameters of AUGUSTUS were only trained once when other parameters had already become stable.

RNA-Seq reads from 454 and Illumina sequencing were mapped against the masked assembly using GSNAP (version 2013-06-27) [99]. The evidence from transcriptome data, protein homology and repeats was input to AUGUSTUS as a ‘hints’ file. The spliced alignments of the RNA-Seq reads using GSNAP resulted in 272,816 unique intron hints and further hints on exonic parts from transcribed regions. Furthermore, we obtained 97,785 hints from ESTs (not only for CDS) using BLAT (version v. 35x1) [102]. The roughly 2.1 million repeat-masked regions were used as ‘nonexonpart’ hints in the annotation, moderately penalizing the prediction of exons overlapping repeats. Consecutive gene sets were computed utilizing AUGUSTUS to stepwise improve prediction accuracy and reliability of the final gene set release referred to as aug3. All extrinsic hint data were incorporated into this last prediction. Allowing the occurrence of alternative transcripts in the results, the final gene set aug3 was then generated using the call:

augustus –species = parasteatoda –alternatives-from-evidence = true … --UTR = on --hintsfile = all.hints --extrinsicCfgFile = extrinsic.P.E.RM.cfg genome_masked.fa

The RNA-Seq data coverage was quantified using the transcript quantification tool eXpress [103], which estimates fragments per kb of transcript per million mapped reads at transcript level (FPKM) values, thereby quantifying the pooled abundances of the predicted transcripts in the RNA-Seq data.

The aug3 gene models were transferred to the Dovetail genome assembly using Exonerate v2.2 [104] with the command --model protein2genome --bestn 1 --showtargetgff YES. The resulting GFF files were converted into protein sets from the corresponding Dovetail genome fasta file.

The Trinotate annotation pipeline (Release 2.0.2) [105] was used for the functional annotation of the aug3 protein predictions following the standard procedure. Briefly, the predicted peptide sequences of the aug3 annotation were blasted against UniRef90 and SwissProt databases with E ≤ 0.05 and keeping only the best hit. HMMER (version 3.1b1) [106] was used to search the Pfam database to predict protein domains. All Blast searches were run in parallel on a high performance computer cluster utilizing the perl script HPC GridRunner (v1.0.2) [107]. The Blast and protein domain predictions were stored in a predefined sqlite (version 3.8.8.3) [108] database. Trinotate was used to export a final report that contains the best Blast hits, protein domain predictions, and GO categories extracted from the Blast result and the Pfam domain prediction for each of the aug3 predictions (Additional file 45: Table S17).

The final annotated gene set contained 27,990 genes and 31,186 transcripts; 85% of the predicted *P. tepidariorum* proteins had homology support derived from a BLASTP search against the UniRef50 data (E value ≤ 10^–5^). Transcript quantification from the RNA-Seq data (using estimates of FPKM values [103]) showed that 29,966 (93%) of predicted transcripts had transcriptome support at FPKM ≥ 0.034 and 26,381 (82%) of predicted transcripts had transcriptome support at FPKM ≥ 0.34. In the final gene set, only 1.1% of the predicted transcripts had neither homology nor transcriptome support at an FPKM threshold of less than 0.034. The annotated *P. tepidariorum* genome is available in JBrowse/Web Apollo Parasteatoda tepidariorum [109].

#### C. sculpturatus

The *C. sculpturatus* genome was annotated using MAKER [110] with RNA-Seq reads generated from a juvenile [111], an adult female [112], and adult males [113]. The annotated *C. sculpturatus* genome is available in the Centruroides Genome Browser [114].

### Analysis of duplicated genes

#### Classification of duplicates using MCScanX

The data used to perform these analyses were, for *P. tepidariorum*, the aug3 version, and for *C. sculpturatus*, the 0.5.53 version of the MAKER annotation available at *Centruroides sculpturatus* MAKER annotation [115]. The same analysis was also performed on the *Limulus polyphemus* genome [116] as a comparison.

Out of the 32,949 gene models in the aug3 annotation of the *P. tepidariorum* genome (resulting from the transfer of the aug3 annotation on the Dovetail scaffolds), only the main transcript of each gene was retained, yielding a set of 28,746 gene models. This list was further shortened by removing all instances of 755 gene models that had become artifactually duplicated during the annotation transfer process from aug2 to aug3, resulting in a final set of 27,203 gene models. All of the 30,465 gene models in the *C. sculpturatus* annotation were retained for the synteny analyses. Finally, out of the 23,287 annotated proteins of *L. polyphemus*, 21,170 were retained for the synteny analyses after filtering out annotated isoforms of the same genes (based on their identical start and end positions).

Hits within and between gene sets were catalogued using BLASTP using an E value threshold of 10^–10^ and keeping only the five best hits as recommended in the instruction manual of MCScanX [117]. Then, MCScanX was used with default parameters to classify genes into five categories, namely singletons (i.e., genes without any duplicate), dispersed (duplicates occurring more than 10 genes apart or on different scaffolds), proximal (duplicates occurring on the same scaffold at most 10 genes apart), tandem (consecutive duplicates), and segmental (block of at least five collinear genes separated by less than 25 genes missing on one of the duplicated regions).

#### Orthology assessment of arthropod genomes

To investigate the extent of gene duplication in *P. tepidariorum* and *C. sculpturatus*, we compared these two genomes to those of four other arthropods with no demonstrable evidence of a WGD. These non-arachnopulmonate taxa were another chelicerate (the tick *I. scapularis*) and three mandibulates (the flour beetle *Tribolium*, the crustacean *Daphnia pulex*, and the centipede *Strigamia maritima*). Predicted peptide sets (aug3) were used as inputs, and redundancy reduction was performed with CD-HIT [118] to remove the variation in the coding regions of genomes attributed to allelic diversity R (>99% sequence similarity). Peptide sequences with all final candidate ORFs were retained as fasta files. We assigned predicted ORFs into orthologous groups across all samples using OMA stand-alone v.0.99u [119, 120] discarding sequences of less than 50 sites in length. All-by-all local alignments were parallelized across 400 CPUs. Orthology mapping of spider and scorpion genes that could be mapped to a mandibulate or tick counterpart was conducted using custom Python scripts on the OMA output.

To assess the possibility of incorrect orthology assessment stemming from algorithmic error, we identified the intersection of the OMA output (based on whole genomes) and a set of orthologs found to occur in single copy across Arthropoda, as benchmarked in the BUSCO-Ar database of OrthoDB [121]. The BUSCO-Ar set of the flour beetle *T. castaneum* was selected as the reference genome for the BUSCO set.

In a separate and subsequent analysis, three additional taxa (genomes of the horseshoe crabs *L. polyphemus*, *Tachypleus gigas*, and *Carcinoscorpius rotundicauda*) were added to the taxa in the principal OMA run, with all other procedures as specified above.

#### Analysis of gene tree topologies from six-genome dataset

From the output of the OMA analysis of six arthropod genomes, we extracted a subset of orthogroups wherein exactly two spider paralogs were present for one *T. castaneum* ortholog (i.e., 1:2 orthology). *T. castaneum* was chosen as the reference genome in comparative analyses both for the quality of its assembly and for its archetypal gene content among Arthropoda. Gene trees for this subset of orthogroups were inferred to examine the topological relationship between homologous sequences of arachnopulmonate and non-arachnopulmonate taxa. These orthogroups were aligned using MUSCLE v.3.8 [122] and ambiguously aligned regions were culled using GBlocks v.0.91b [123] using the commands –b3 = 8 (maximum of eight contiguous non-conserved positions), –b4 = 10 (minimum of ten positions in a block), and –b5 = h (gap positions allowed for a maximum of half the sequences). Maximum likelihood analyses were conducted using the LG + Γ model with four rate categories [124, 125] and 500 independent starts in RAxML v. 7.3.0 [126].

We characterized whether the resulting tree topologies corresponded to Hypothesis 1 (common duplication in the most recent common ancestor (MRCA) of spiders and scorpions), Hypothesis 2 (lineage-specific duplication events in each of spiders and scorpions), an indeterminate tree topology (corresponding to neither scenario, typically due to the non-monophyly of the outgroup taxa), or an uninformative tree topology (due to the lack of any scorpion paralogs). Cases where the two spider paralogs formed a grade with respect to a single scorpion paralog were additionally classified as partially congruent with Hypothesis 1. The set of gene trees either partially or fully congruent with Hypothesis 1 is henceforth termed “Tree Set 1”. Alignments and gene tree files are available on request.

#### Analysis of gene tree from nine-genome dataset

To infer the relationship between arachnopulmonate and xiphosuran paralogs, from the OMA analysis of nine genomes (the six genomes above, *L. polyphemus*, *T. gigas*, and *C. rotundicauda*) we separately extracted another subset of orthogroups, wherein two, three, or four horseshoe crab paralogs from any of the three horseshoe crab genomes were detected for one *T. castaneum* ortholog (i.e., 1:2, 1:3, or 1:4 orthology). We inferred gene trees with the approach specified above. We again distinguished two scenarios, namely (1) separate WGD events in the MRCA of Arachnopulmonata and Xiphosura (Hypothesis 3), and (2) a common WGD event in the MRCA of all Chelicerata (Hypothesis 4). Cases where ancient paralogy was detected in Xiphosura alone (and not Arachnopulmonata) were classified as partially congruent with Hypothesis 3. The set of gene trees either partially or fully consistent with Hypothesis 3 was termed “Tree Set 2”. Alignments and gene tree files are available on request.

#### Identification of paralog pairs in *P. tepidariorum* and other chelicerates

Putative families of homologous protein-coding genes were identified for 31 chelicerate species and a myriapod (Additional file 14: Table S8). Protein sequences from the publically available translated coding sequences were also used. Otherwise, transcripts were translated with Transdecoder [97]. For translated sequences with > 95% identity, only the single longest protein was retained for further analyses. For transcripts assembled by Trinity [127], the longest transcript per “contig” was retained (Trinity often generates multiple transcripts associated with a single “contig”, thought to represent isoforms).

We grouped genes into families using a modified version of the method applied in the Phytozome project described by Goodstein et al. [61], with either *P. tepidariorum* or *C. sculpturatus* translated genes used as a seed. In short, homologous protein pairs were identified using all-versus-all BLASTP comparisons of the 32 arthropod species with an E cutoff value of < 1 × 10^–3^ [101]. A global alignment score was calculated for each homologous pair using the Needleman–Wunsch algorithm with the Blosum62 matrix. We then used the Needleman–Wunsch score between *P. tepidariorum* (or *C. sculpturatus*) protein sequences and the rest of the sequences to seed the gene families in a three-step process. First, for each non-*P. tepidariorum* protein, the *P. tepidariorum* protein with the highest Needleman–Wunch score was identified. Second, all the non-*Parasteatoda* proteins with the same best-scoring *P. tepidariorum* protein were grouped with the *P. tepidariorum* protein. Third, all the groups were combined that contained *P. tepidariorum* proteins determined to be homologous to each other based on a BLASTP alignment with an E value of < 1 × 10^–3^. The same three-step process was repeated to identify *C. sculpturatus*-seeded gene families.

For each gene family, the protein sequences were multiply aligned using MUSCLE [122]. The multiple alignments were trimmed by removing all the bounding alignment positions that added more gaps than sequence by a custom Perl script. Entire protein sequences were removed from the alignment if the sequence had gaps in more than 25% of the aligned positions. For the *P. tepidariorum*-seeded gene families, only those containing at least one *P. tepidariorum* protein and four additional sequences were retained for further analyses. Within the retained families, poorly aligned columns were removed using TrimAL under a “strict-plus” setting, which optimizes the signal to noise ratio in the multiple alignment [128]. The protein alignments were then used to guide nucleotide alignments by replacing the amino acids with their encoding transcript sequences.

Protein alignments were used to infer gene trees with TreeBeST [129]. TreeBeST searches for an optimal gene tree given a species tree (we used the phylogeny in Additional file 15: Figure S7) and identifies duplication and speciation events within the optimal tree. Branch lengths were calculated for the optimal TreeBeST tree using maximum likelihood (PhyML type search) with the HKY model of evolution [62]. Alignments and gene tree inferences were repeated for the *C. sculpturatus*-seeded gene families.

#### Molecular distance of duplication and speciation events

We estimated the molecular distance of a *P. tepidariorum* (or *C. sculpturatus*) duplication or speciation node in *P. tepidariorum* (or *C. sculpturatus*)-seeded families by averaging the branch lengths in TreeBeST trees from the node to all its *P. tepidariorum* (or *C. sculpturatus*) descendants. We similarly estimated the molecular distance of other species’ duplication nodes by averaging the branch length from the node to all of the descendants of the species of interest. Distributions of molecular distances were estimated and statistical tests for goodness-of-fit calculated in R.

#### Ascertaining GO Term enrichment in *P. tepidariorum* paralog pairs

GO Terms were imputed to the *P. tepidariorum* AUGUSTUS gene models (aug3) through comparisons to the UniRef50 protein set by BLASTP comparisons using a cut-off of 1 × 10^–5^. The GO Terms of its closest UniRef by E value with documented GO Terms were assigned to a gene model via a custom perl script, with GO Slim values derived using GOSlimViewer [130]. Enrichment of GO Terms within gene families was ascertained using Fisher’s exact test.

#### Synteny analyses

A genome-scale synteny analysis of the *P. tepidariorum* scaffolds was conducted using the program SatsumaSynteny [60]. This approach does not rely on the annotation and can detect weak, degraded signals of synteny such as signatures of ancient WGDs that were followed by numerous rearrangements. For visualization, we selected only the 100 scaffolds for which the number of hits detected by Satsuma was maximal; in a second round, this list was further reduced to the set of 39 scaffolds that exhibited the greatest number of hits with each other. An Oxford grid [131] was drawn using the tool orthodotter [132], and a circular plot was drawn using Circos [133].

For the synteny analysis of selected developmental genes, their nucleotide sequences were first downloaded from NCBI (Accession numbers are given in Additional file 12: Table S7). BLASTN searches against the Augustus 3 gene set were used to identify the best aug3 prediction and BLASTN searches against the Dovetail assembly (Assembly 2.0) were used to identify their respective scaffold.

All 148 precursor microRNA sequences for *P. tepidariorum* [44], with the inclusion of flanking sequences 20 bp up- and down-stream, were BLASTN-searched in the Dovetail assembly to identify scaffold ID and position from the best matches. The scaffolds and positions of *C. sculpturatus* microRNAs from Leite et al. [44] were used.

#### Homeobox and Hox gene annotation

To identify possible homeobox genes in *P. tepidariorum* and *C. sculpturatus*, the complete set of homeodomain sequences from HomeoDB [134, 135], those identified previously in the scorpion *Mesobuthus martensii* [36], and the *P. tepidariorum* gene *prospero* (Accession: BAE87100.1) were BLASTP-searched (version 2.4.0+) [136] against the *P. tepidariorum* AUGUSTUS (aug3) and *C. sculpturatus* MAKER protein predictions. All blast hits were scanned for the presence of homeodomains and other functional domains with the CDD search tool [137]. Hits that contained at least one homeodomain were manually checked for the completeness of this sequence. The homeobox genes were annotated and classified based on the work by Holland et al. [138].

To identify the location of Hox genes on genomic scaffolds of *P. tepidariorum*, *Latrodectus hesperus*, *S. mimosarum*, *A. geniculata*, *C. sculpturatus*, *I. scapularis*, and genomic contigs of *M. martensii* were searched for Hox genes with tblastx BLAST (version 2.2.28+) [136] using published chelicerate Hox gene sequences. Scaffolds or contigs containing blast hits to Hox genes were extracted and intron-exon boundaries were hand-annotated in Geneious (version 7) [139] with the help of sequenced transcriptomes, sequences obtained by RACE PCR experiments (in the case of *P. tepidariorum*), cloned Hox gene sequences (in the case of *C. sculpturatus*), or by comparison between the chelicerate sequences. In case of additional splice variants containing additional small exons, the shortest version consisting of only two exons was used for the analysis. Naming of Hox genes followed orthologies to already published Hox genes in *C. salei* and *P. tepidariorum* for the spider sequences or, in the case of the scorpions, orthologies to published *C. sculpturatus* sequences.

#### Hox gene alignments and topological tests of gene trees

Nine Hox class genes were used as test cases for distinguishing two scenarios, namely (1) common duplication in the MRCA of spiders and scorpions (Hypothesis 1) and (2) lineage-specific duplication events in each of spiders and scorpions (Hypothesis 2). The single remaining Hox class gene (*fushi tarazu*) did not possess the minimum requirement – inclusion of two paralogs each of a spider and a scorpion species – and thus was not dispositive in topological tests. Peptide sequence alignments were constructed using MUSCLE v. 3.8 [122] and alignment ends manually trimmed, such that either terminus of the alignment sampled at least half of each alignment’s terminals. Preliminary efforts using outgroup taxa have demonstrated little statistical power resulting from rooting trees due to large phylogenetic distances between arachnopulmonates and arachnid outgroups (e.g., harvestmen, pycnogonids [40]), as well as accelerated evolution in other potential outgroup taxa (e.g., mites, ticks [53]). Therefore, outgroup-free tests were conducted using spider and scorpion sequences only.

Maximum likelihood analyses were conducted using the LG + Γ model with four rate categories [124, 125] and 500 independent starts in RAxML v. 7.3.0 [126]. To compare tree likelihoods of unconstrained runs to Hypothesis 2, a constraint tree was imposed for each Hox class enforcing mutual monophyly of spider and scorpion sequences, and the best tree topology was selected from 500 independent starts under the scenario of lineage-specific duplications.

### Embryos, in situ hybridization, and imaging

*P. tepidariorum* embryos were obtained from laboratory cultures in Oxford, UK, Cambridge, MA, USA, and Cologne, Germany. RNA was extracted from embryos of stages 1–14 using either Trizol (Life Technologies) or Qiazol (Qiagen) and cDNA was synthesized with SuperscriptIII (Life Technologies). Probe templates were either synthesized by PCR using TOPO pCR4 vectors containing cloned RACE fragments of Hox genes (RACE was performed with the Marathon RACE kit or SMART RACE cDNA kit (Clontech)), or they were generated by adding T7 binding sites to RT-PCR fragments as described previously [140]. Primer sequences used for the RT-PCR fragments were based on the *P. tepidariorum* transcriptome [86] and genome sequences. The origin of gene fragments and primers is available on request. Embryos were fixed and probe synthesis and in situ hybridizations were carried out as described previously [141, 142]. The anti-DIG antibody (Roche, 11093274910) was pre-absorbed overnight at 4 °C with mixed-stage embryos. Stained embryos were staged according to Mittmann and Wolff [143] and imaged using a Leica stereoscope fitted with a Zeiss AxioCam MRc. Images were processed in Photoshop CS4 or CS6.

## Additional files

Additional file 1: Table S1. Duplicated genes in *Parasteatoda* and *Centruroides* in comparison to each of the single-copy arthropods. (XLSX 55 kb)

Additional file 2: Figure S1. Duplicated genes: three gene blocks. (PDF 36 kb)

Additional file 3: Figure S2. Duplicated genes: four gene blocks. (PDF 36 kb)

Additional file 4: Figure S3. Duplicated genes: five gene blocks. (PDF 36 kb)

Additional file 5: Table S2. *P. tepidariorum* homeobox genes. (XLSX 56 kb)

Additional file 6: Table S3. *C. sculpturatus* homeobox genes. (XLSX 54 kb)

Additional file 7: Figure S4. Gene tree analysis of individual Hox genes. Blue branches indicate scorpion sequences; red branches indicate spider sequences. Gene trees are shown for *labial* (A), *proboscipedia* (B), *Deformed* (C), *Sex combs reduced* (D), *Antennapedia* (E), *Ultrabithorax* (F), *abdominal-A*, (G), and *Abdominal-B* (H). Log likelihood values are provided in Fig. 4. Due to insufficient sequences for hypothesis testing, gene trees were not inferred for *Hox3-B* and *fushi tarazu*. Abbreviations: *Pt Parasteatoda tepidariorum*, *Lh Latrodectus hesperus*, *Sm Stegodyphus mimosarum*, *Ag Acanthoscurria geniculata*, *Cs Centruroides sculpturatus*, *Mm Mesobuthus martensii*. (PDF 156 kb)

Additional file 8: Figure S5. *P. tepidariorum* Hox clusters to scale. The distances of Hox genes in clusters A and B are very similar, except for the break in cluster B. Hox genes are colored so that paralogs match, non-Hox genes are shown in grey and microRNAs are represented as lines. Genes on the positive strand and negative strand are indicated above and below the black lines, respectively. (JPG 503 kb)

Additional file 9: Table S4. Spider, scorpion and mite Hox clusters A and B. (XLSX 533 kb)

Additional file 10: Table S5. microRNAs and other genes identified in the *P. tepidariorum* Hox clusters. (DOCX 106 kb)

Additional file 11: Table S6. Duplication of miRNAs by possible large-scale and tandem duplication events in *Parasteatoda* and *Centruroides. (XLSX 52 kb)*

Additional file 12: Table S7. Duplicated developmental genes in *P. tepidariorum*. (XLSX 50 kb)

Additional file 13: Figure S6. Synteny of 39 *P. tepidariorum* scaffolds with greatest number of reciprocal hits. Circos plot of the subset of 39 scaffolds presenting the greatest numbers of hits on one another (as detected using SatsumaSynteny). One unit on the perimeter represents one Mbp. (PDF 819 kb)

Additional file 14: Table S8. Genomic and transcriptomic datasets used to build gene families. (DOCX 141 kb)

Additional file 15: Figure S7. Phylogeny of 31 arthropod species used in comparison with *P. tepidariorum*. Species relationships are based on JC Regier, JW Shultz, A Zwick, A Hussey, B Ball, R Wetzer, JW Martin, and CW Cunningham [73], JE Bond, NL Garrison, CA Hamilton, RL Godwin, M Hedin, and I Agnarsson [144], PP Sharma and G Giribet [145]. The number of genes within *P. tepidariorum*-seeded gene families are shown parenthetically (n =), the number of speciation nodes observed in the gene families between *P. tepidariorum* and the other species (N =), and the median HKY distance between each speciation node and *P. tepidariorum* (HKY =) descendants are shown at the node. (DOCX 348 kb)

Additional file 16: Table S9. *P* values of Kolmogorov–Smirnov goodness-of-fit tests for five models of the HKY distance^1^ distribution of duplication nodes with non-*P. tepidariorum* descendants. Distributions unable to be rejected at an alpha level of 0.05 are in bold. Best fitting models are in italics. (DOCX 80 kb)

Additional file 17: Figure S8. HKY distance distributions and Gaussian mixture models of duplication nodes from *P. tepidariorum*-seeded gene families for eight arachnid species. The HKY distances for duplication nodes were calculated as the mean HKY branch length from the duplication node to each of the descendent genes in the species of interest (A–H). In panel (I) the distribution is for all the duplication nodes with at least one *P. tepidariorum* descendant, using the mean HKY distance from the node to *P. tepidariorum* descendants. For each panel, the best match to one of five distributions (Uniform, exponential (G), or a Gaussian mixture model with 1, 2 (H), or 3 (A–F, I) distributions) is shown. The Gaussian mixture models were seeded with Gaussian mean and standard deviations estimated from the *P. tepidariorum* duplication nodes (Fig. 6a). (DOCX 430 kb)

Additional file 18: Table S10. Percent of duplication nodes assigned to three Gaussian distributions of HKY distances. The mean and standard deviation of the *P. tepidariorum* distributions (Fig. 6a) were used to estimate the other species’ Gaussian distributions. (DOCX 65 kb)

Additional file 19: Figure S9. HKY distance distributions of all *P. tepidariorum* speciation nodes in *P. tepidariorum*-seeded gene families. The distributions are for mean HKY branch lengths from the *P. tepidariorum* speciation node to the descendant *P. tepidariorum* genes. (DOCX 244 kb)

Additional file 20: Table S11. Comparison of distributions for *P. tepidariorum* duplication and speciation nodes. Kolmogorov–Smirnov goodness-of-fit tests were used to compare the middle Gaussian distribution of HKY distances of duplication nodes (Fig. 6a, Additional file 13: Figure S6) to the log-normal distributions for speciation nodes. (DOCX 73 kb)

Additional file 21: Table S12. Shared paralog pair retention between *P. tepidariorum* and other arthropod species. Shaded species have complete genomes or deeply sequenced transcriptomes. (DOCX 92 kb)

Additional file 22: Figure S10. Tandem duplications are abundant in young duplication events, but rare in older duplication events. HKY distance distributions and Gaussian mixture models of duplication nodes from *P. tepidariorum*-seeded gene families mirror those in Fig. 6a, but paralog pairs that are found in tandem (within five genes of each other on the same scaffold) are now shown in light grey, and dispersed paralogs in dark grey. (PDF 158 kb)

Additional file 23: Table S13. Analysis of the gene families containing a duplication pair from each of the Gaussian distributions (Fig. 6a), excluding tandem duplicates, show enrichment in several GO terms compared to gene families without duplication pairs. (XLSX 2339 kb)

Additional file 24: Table S14. Gene IDs of duplicated *P. tepidariorum* and *C. sculpturatus* genes compared to other arthropods. (XLSX 86 kb)

Additional file 25: Table S15. Frequency of tree topologies supporting different duplication scenarios. (XLSX 34 kb)

Additional file 26: Table S16. Orthology of *P. tepidariorum* genes giving full or partial support for WGD with respect to *T. castaneum*. (XLSX 19 kb)

Additional file 27: Figure S11. *P. tepidariorum proboscipedia-A* expression. *pb-A* is first expressed in a thin stripe in the anterior of the embryo at stage 7 (*A* and *B*). The stripe broadens and as segmentation commences, it is found in the pedipalpal segment, and additionally, four weaker stripes start to appear in L1–L4 (*C*). The expression in the pedipalpal segment remains strongest, when by the end of stage 8.1, another stripe of weak *pb-A* expression is found in O1 (arrow) (D and E). During stage 9 and 10, *pb-A* expression is found in the mesoderm of the outgrowing pedipalp and walking legs, but also ectodermally in the distal tip of the outgrowing appendages of Pp-L4 (caret) (F–H). In the nervous system, *pb-A* is expressed most strongly in the Pp segment, starting at stage 9 (arrowhead), but it is also present in a smaller lateral domain of L1–O1 (arrow) (F–H). Each panel shows the same embryo, viewed laterally (left) and ventrally (right, or center and right in E–H). Anterior is to the left. Abbreviations: *Pp* pedipalpal segment, *L* walking leg segments, *O* opisthosomal segments. (JPG 6345 kb)

Additional file 28: Figure S12. *P. tepidariorum proboscipedia-B* expression. Weak *pb-B* expression emerges at stage 8 in a domain spanning from Pp to L4 (A). The weak expression is located in the mesoderm, and at stage 8.2 also visible in O1 (arrow) (B). At stages 9 and 10, *pb-B* is expressed in the mesoderm of the outgrowing appendages of Pp–L4, as well as in dorsolateral parts of the neuroectoderm of segments Pp–O1 (arrows) (C and D). Each panel (except A) shows the same embryo, viewed laterally (left) and ventrally (center, right). Anterior is to the left. Abbreviations: see Additional file 27: Figure S11. (JPG 143 kb)

Additional file 29: Figure S13. *P. tepidariorum Hox3-A* expression. *Hox3-A* expression appears first at stage 7 (not shown). At stage 8.1, the expression can be found in segmental stripes in the pedipalpal and the walking leg segments, and weak expression can also be seen in O1. The strongest expression is found in the first walking leg segment (A). At stage 8.2 and 9.1, *Hox3-A* is expressed in the mesoderm of the developing limb buds (B, C) and in the mesoderm of posterior segments. Note that *Hox3-A* expression is very weak and due to the high background in the head segments as well as in the opisthosomal segments, it is difficult to define anterior/posterior boundaries. Expression of *Hox3-A* vanishes after stage 9 (not shown). Panels show flat-mounted embryos, brightfield image above, nuclear stain of the same embryo below. Anterior is to the left. Abbreviations: see Additional file 27: Figure S11. (JPG 214 kb)

Additional file 30: Figure S14. *P. tepidariorum Hox3-B* expression. *Hox3-B* is not expressed before stage 8, when expression starts in broad segmental stripes from Pp to L4, most likely in the mesoderm (A). Expression is strongest in L1 and L4 (A and B). In stage 9 and 10 embryos, *Hox3-B* is expressed in the mesoderm of the outgrowing limb buds, except for the pedipalps, which show a broad ring of expression (white arrow in C) that later refines into rings and additional expression at the tip of the pedipalp (D–F). Additionally, expression extends anterior-ventral to the limb buds in a triangular shape at stage 9 (arrowhead in C). This expression vanishes by stage 10. Instead, *Hox3-B* is now also expressed in the pedipalpal and walking leg segments in segmental groups of cells in the medial neuroectoderm (arrowheads in D–F). In stage 11 embryos, *Hox3-B* is additionally expressed in dots in the ventral neuroectoderm of every opisthosomal segment (carets in F). Furthermore, a dot of expression can be found in the first opisthosomal limb bud (arrow in F). Embryos in A and B are shown laterally, embryos C–E are shown laterally on the left and ventrally on the right. F shows ventral views of the head region (left) and the opisthosoma (right) of an embryo at a similar stage as the embryo in E. Anterior is to the left. Abbreviations: see Additional file 27: Figure S11. (JPG 4203 kb)

Additional file 31: Figure S15. *P. tepidariorum Deformed-A* expression. *Dfd-A* is first expressed at stage 4, in almost all cells but the rim (arrowhead) of the germ disc (A). During stage 6, cells not expressing *Dfd-A* at the outer rim, the future anterior, have multiplied (arrowheads) (B). Later during stage 6, the expression clears from the posterior (former center of the germ disc, arrow) and the expression starts to form a broad stripe (bracket) (C). This uniform stripe of expression first subdivides into two stripes with lower expression between these stripes during stage 7. Then, the posterior domain splits into two more stripes and, in between the anterior and posterior domains, a new stripe gets inserted such that there are now four stripes of *Dfd-A* expression (asterisks) (D). These four stripes are later located in the four walking leg segments at stage 8 (E). In stage 9–11 embryos, *Dfd-A* is strongly expressed in the outgrowing walking legs with strongest expression in the tips of the legs (white carets) (F–H) and in the neuroectoderm (arrowheads in G and H). Each panel (except for A, B, C) shows the same embryo, viewed laterally (left) and ventrally (right). Anterior is to the left in all panels. Abbreviations: See Additional file 27: Figure S11. (JPG 6387 kb)

Additional file 32: Figure S16. *P. tepidariorum Deformed-B* expression. *Dfd-B* expression is first detected in a weak, broad stripe in the developing germ band (bracket) (A). This stripe can be allocated to the first two walking leg segments at stage 8 (B). The anterior of the segments is stained stronger than the posterior. Additional, but much weaker expression can be seen in the L3 and L4 segments. At stage 9, *Dfd-B* is predominantly expressed in the ventral neuroectoderm (C). The anterior and posterior expression domain is marked by arrowheads in B and C, respectively. Each panel shows the same embryo viewed laterally (left) and ventrally (right). Anterior is to the left. Abbreviations: See Additional file 27: Figure S11. (JPG 2272 kb)

Additional file 33: Figure S17. *P. tepidariorum Sex combs reduced-A* expression. A stripe of *Scr-A* expression first appears at stage 6 (A). The stripe broadens during stage 7 (B), at the end of which it starts to split into two stripes, and a third new stripe appears posterior to the initial stripes (borders of expression marked by arrowheads) (C). At stage 8.1, there are four stripes located in the posterior parts of L2, L3, and L4 and the newest stripe appears in O1 (white arrow) (D and E). In limb bud stages, only the L3 and L4 limb buds carry *Scr-A* expression, mostly in their distal tips. Expression in L2 is restricted to the neuroectoderm (not shown), and expression in O1 is restricted to the posterior part of each hemisegment as well (F). At later stages, *Scr-A* expression in L3 and L4 refines to several rings in the distal part of the legs. Expression in L2 becomes undetectable. (G–I). At stage 10.1, *Scr-A* is visible in a neuroectodermal patch in L4 and a dot in O1 (arrow) (H). Each panel shows the same embryo viewed laterally (left) and ventrally (center and right). Anterior is to the left. Abbreviations: See Additional file 27: Figure S11. (JPG 266 kb)

Additional file 34: Figure S18. *P. tepidariorum Sex combs reduced-B* expression. Scr*-B* is first expressed in a cap-like domain (arrowhead) in the center of the germ disc at stage 5. The position of the cumulus is marked by a ‘c’ (A). The expression widens during stage 6. *Scr-B* now forms an open ring, localized roughly halfway between anterior rim of the opening germ disc and the future posterior center of the germ disc. The cells posterior to the ring also express *Scr-B*, but at a much lower level (B). At stage 7, the ring splits up into two stripes (arrowheads) (C) and, later, two new stripes appear (carets) (D). The most anterior stripe of expression lies between L1 and L2 at stage 8, the second anterior stripe lies between L2 and L3. The two posterior stripes cover L3 and L4. The weak expression of *Scr-B* continues posterior to these stripes (E and F). *Scr-B* is predominantly expressed in the limb buds and the ventral neuroectoderm of stage 9.1 embryos (G). The anterior expression border is directly posterior to the L1 limb bud. Expression of *Scr-B* continues in the opisthosoma, but is much weaker, except for a domain in the SAZ (arrow) (G). This expression in the SAZ continues to the end of segmentation (H and I). No more expression is visible at the posterior end of the embryo at stage 11 (white caret) (J). *Scr-B* expression forms multiple rings in the legs (H–J), but it is much more strongly expressed in L3 compared to L2 and L4. At late stage 9, the expression of *Scr-B* in the neuroectoderm can also be found in every segment of the opisthosoma (arrowhead in H). Each panel shows the same embryo viewed laterally (left) and ventrally (center and right). Anterior is to the left. Abbreviations: See Additional file 27: Figure S11. (JPG 393 kb)

Additional file 35: Figure S19. *P. tepidariorum fushi tarazu* expression. *ftz* is first expressed at stage 6 in a semicircle at the posterior end where the SAZ is forming (arrow) (A). The bracket marks an anterior broad stripe of *ftz* expression. This domain of expression gets weaker during stages 7 and 8, where mostly only the anterior border of the domain anterior to L2 (arrowhead in D) is visible. The expression is almost invisible when it is viewed ventrally. The expression in the SAZ persists, and does not clear from the posterior end (A–G). It emanates stripes at the anterior end of the SAZ (arrow in C–G). Stripes stay visible at the anterior border of the last formed segment (white arrow in D and G) until the next stripe of *ftz* expression forms. Only after segmentation finishes does the expression at the posterior disappear (arrow in H). Meanwhile, the anterior border of the Hox expression domain stretches from L2 to L4 and is predominantly found ventrally (black arrowheads in F and H). The legs on one side of a stage 11 embryo were removed to show the ventral Hox domain, which has concentrated to one spot per hemisegment (black arrowheads) within the L2–L4 domain (H). At stage 10, *ftz* expression appears in a ring near the distal tip of L3 (carets in G–H). Each panel shows the same embryo, viewed laterally (left) and ventrally (right, or center and right in E*–*H). Anterior is to the left. Abbreviations: See Additional file 27: Figure S11. (JPG 7681 kb)

Additional file 36: Figure S20. *P. tepidariorum Antennapedia-A* expression. *Antp-A* expression first develops in the SAZ at stage 7 (A). This expression transforms into a stripe and is followed by cells that show only weak expression of *Antp-A* (B), before new expression appears at the posterior end of the SAZ (C). The first two stripes can be allocated to the first two opisthosomal segments at stage 8 (D). New stripes keep appearing from the SAZ (arrows, E–H) until the end of segmentation (G). At stage 9, the anterior border of *Antp-A* expression reaches into the posterior half of L4 (arrowhead, E). Expression is now also seen in the ventral neuroectoderm, where it forms longitudinal rows in each hemisegment (white arrowheads, E–G), and rings in the L4 appendage (caret, F and G). Throughout development, *Antp-A* expression is always strongest in O1 and the anterior half of O2. Each panel shows the same embryo, viewed laterally (left) and ventrally (right, or center and right in E*–*G). Anterior is to the left. Abbreviations: See Additional file 27: Figure S11. (JPG 6657 kb)

Additional file 37: Figure S21. *P. tepidariorum Antennapedia-B* expression. *Antp-B* first emerges in the first two opisthosomal segments at stage 8.2 (arrows) (A). The *Antp-B* domain expands anteriorly into the posterior part of L4 during stage 9 (white arrowheads) (B–D). Additionally, *Antp-B* forms rings of expression in the L4 appendage (carets in C and D). While initially the very posterior part of O2 is free of *Antp-B* expression (the O2/O3 border is demarcated by a black vertical line in D–F), during stage 10, expression can also be found at the O2/O3 border (arrows) (F). Furthermore, during stage 10, the expression pattern refines, in that the L4 and O1 *Antp-B* expression is mostly in the ventral neuroectoderm and not found dorsally, while the O2 expression is excluded from the most ventral region (E–G). Starting at stage 10, *Antp-B* is also expressed in one dot each in the opisthosomal limb buds on O4 and O5 (arrowheads) (E–G). Each panel shows the same embryo, viewed laterally (left) and ventrally (right, or center and right in E–G). Anterior is to the left. Abbreviations: See Additional file 27: Figure S11. (JPG 5223 kb)

Additional file 38: Figure S22. *P. tepidariorum Ultrabithorax-A* expression. *Ubx-A* is first expressed at stage 8.1 in the SAZ and in a weak stripe anterior to that (arrow) (A). This stripe gets stronger and the posterior domain enlarges (B and C) so that all new tissue added posteriorly expresses *Ubx-A* at equal levels (D–F). The anterior border is located in O2, while the very anterior part of O2 initially does not express *Ubx-A* (segmental boundaries in C–F indicated by black vertical lines) until the end of stage 9.2, when it is expressed dorsally and in the neuroectoderm also in the anterior part of O2 (white arrow in E). Each panel shows the same embryo, viewed laterally (left) and ventrally (right). Anterior is to the left. Abbreviations: See Additional file 27: Figure S11. (JPG 4678 kb)

Additional file 39: Figure S23. *P. tepidariorum Ultrabithorax-B* expression. *Ubx-B* is first expressed at stage 8.2, in the SAZ, as well as in two weaker stripes in the O3 and O4 segments (arrows) (A). Expression is initially restricted to the posterior part of O3 (B), but eventually the anterior expression border extends ventrally into the posterior part of O2 (C–F), and is also found in the posterior part of the O2 limb bud (caret in E and F). Expression is strongest in O3, but otherwise is fairly uniform in all segments posterior to this one (D–F). Each panel shows the same embryo, viewed laterally (left) and ventrally (right). Anterior is to the left. Abbreviations: See Additional file 27: Figure S11. (JPG 4129 kb)

Additional file 40: Figure S24. *P. tepidariorum abdominalA-A* expression. *abdA-A* expression starts in the SAZ during stage 9.1, posterior to O5 (A and B). By stage 10.1, the anterior border of *abdA-A* expression has extended into the posterior part of O3 (arrowhead, C). While the dorsal border of *abdA-A* expression remains there, in the ventral neuroectoderm, the expression expands anteriorly into the posterior half of O2 (arrowhead, D). These borders of expression persist during later development (E to G). Each panel shows the same embryo, viewed laterally (left) and ventrally (right). Anterior is to the left. Abbreviations: See Additional file 27: Figure S11. (JPG 5068 kb)

Additional file 41: Figure S25. *P. tepidariorum abdominalA-B* expression. *abdA-B* expression first appears at stage 9.1 in the SAZ (A). Slightly later, shortly before the opisthosomal limb buds appear, *abdA-B* additionally emerges in the posterior part of O4 (B). From stage 9.2 onwards, *abdA-B* is expressed in the entire O4 segment and all posterior segments with strong expression in the opisthosomal limb buds (C–F). Each panel shows the same embryo, viewed laterally (left) and ventrally (right). Anterior is to the left. Abbreviations: See Additional file 27: Figure S11. (JPG 4092 kb)

Additional file 42: Figure S26. *P. tepidariorum AbdominalB-A* expression. *AbdB-A* is first expressed at stage 9.1 in O6 (arrowhead) and in a stripe in the anterior-most portion of the SAZ (A). The anterior expression border subsequently shifts anteriorly, first into the posterior part of O5 (arrowhead) (B and C), and the dorsal part of the *AbdB-A* domain then shifts into the posterior portion of O4 (arrowhead) (D and E). From the beginning of opisthosomal limb bud development, it is strongly expressed in the O5 limb buds. At stage 9.2, the ventral part of the *AbdB-A* domain expands into the posterior half of O2 (arrow). These anterior borders remain the same throughout the rest of embryonic development (E–G). Each panel shows the same embryo, viewed laterally (left) and ventrally (right, or center and right in D–G). Anterior is to the left. Abbreviations: See Additional file 27: Figure S11. (JPG 245 kb)

Additional file 43: Figure S27. *P. tepidariorum AbdominalB-B* expression. *AbdB-B* expression emerges with the appearance of the SAZ at stage 6 (A). It is continuously expressed in the SAZ until the end of segmentation (caret) (B–H). Additionally, from stage 9.1 on, weak *AbdB-B* expression is found posterior to the O3/O4 border (arrowhead) (F). Slightly later, additional expression appears in the O2 limb buds, in the prospective genital opening (arrow) (G and H). Dorsally, the *AbdB-B* expression domain extends from O5 to the posterior end (H–J), but in the ventral neuroectoderm, the *AbdB-B* expression border is located in the posterior half of O3 (arrowhead in I). The vertical line delineates the O2/O3 border, which the *AbB-B* expression does not reach in the neuroectoderm (J). Each panel shows the same embryo, viewed laterally (left) and ventrally (right, or center and right in G–J). Anterior is to the left. Abbreviations: See Additional file 27: Figure S11. (JPG 332 kb)

Additional file 44: Supplementary File 1. Hox gene expression in *P. tepidariorum*. Detailed description and comparison of the expression patterns of the Hox gene paralogs. (DOCX 143 kb)

Additional file 45: Table S17. Trinotate report of the best Blast hits, protein domain predictions and GO categories extracted from the Blast result, and the Pfam domain prediction for each of the aug3 predictions. (XLSX 7616 kb)
