# Supplementary material for: The house spider genome reveals an ancient whole-genome duplication during arachnid evolution
Source: BMC Biol. 2017 Jul 31;15:62. doi: 10.1186/s12915-017-0399-x (PMC5535294; doi:10.1186/s12915-017-0399-x)

Intragenomic comparisons:

	PtPt	CsCs	LpLp
Singletons	6323	13073	4236
Dispersed	14871	14510	14577
Proximal	2233	1114	97
Tandem	3726	1716	2066
Segmental	50	52	194
Total	27203	30465	21170
N50 scaffolds(kb)	4055	342	254

ftp://ftp.hgsc.bcm.edu/I5K-pilot/Bark_scorpion/
ftp://ftp.ncbi.nih.gov/genomes/Limulus_polyphemus/

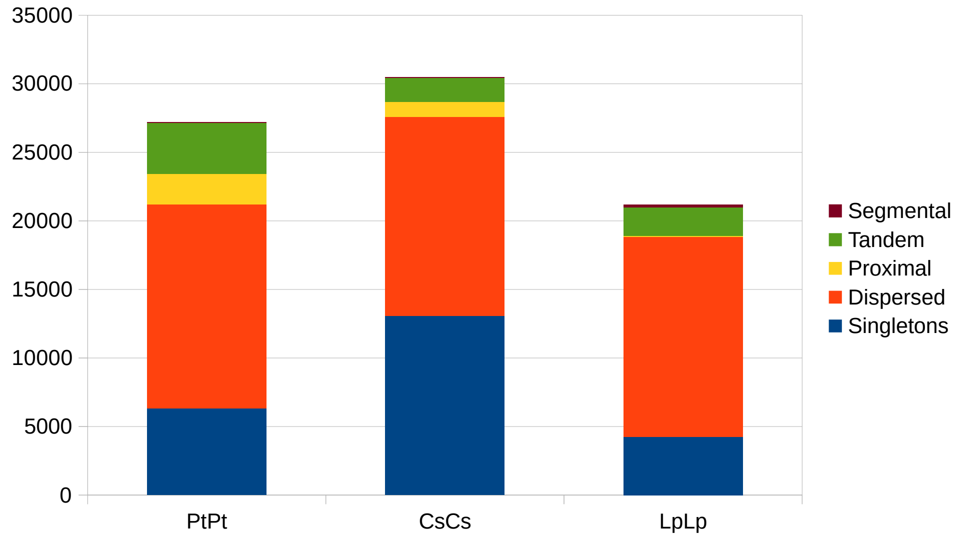

Intergenomic comparisons:

	LpCs	LpPt	CsPt
Singletons	17367	17139	23445
Dispersed	34236	31214	34077
Proximal	0	0	0
Tandem	0	0	0
Segmental	32	20	146
Total	51635	48373	57668

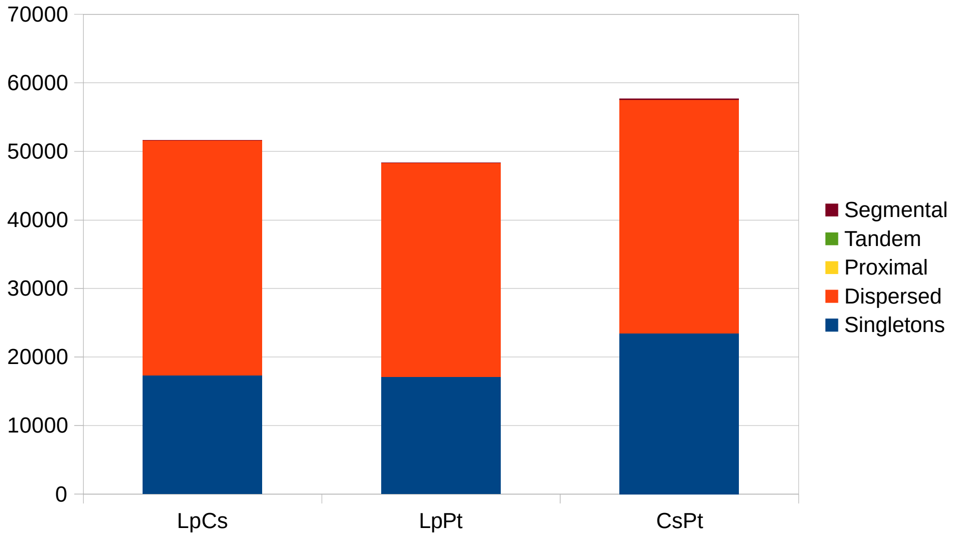

Supplement: Supplementary file 2 — Duplicated genes: three gene blocks. (PDF 36 kb) [file 12915_2017_399_MOESM2_ESM.pdf]
