# Supplementary material for: The house spider genome reveals an ancient whole-genome duplication during arachnid evolution
Source: BMC Biol. 2017 Jul 31;15:62. doi: 10.1186/s12915-017-0399-x (PMC5535294; doi:10.1186/s12915-017-0399-x)

5 genes

Intragenomic comparisons:

	PtPt	CsCs	LpLp
Singletons	6323	13073	4236
Dispersed	14908	14561	14654
Proximal	2236	1114	97
Tandem	3736	1717	2077
Segmental	0	0	106
Total	27203	30465	21170
N50 scaffolds(kb)	4055	342	254

ftp://ftp.hgsc.bcm.edu/I5K-pilot/Bark_scorpion/
ftp://ftp.ncbi.nih.gov/genomes/Limulus_polyphemus/

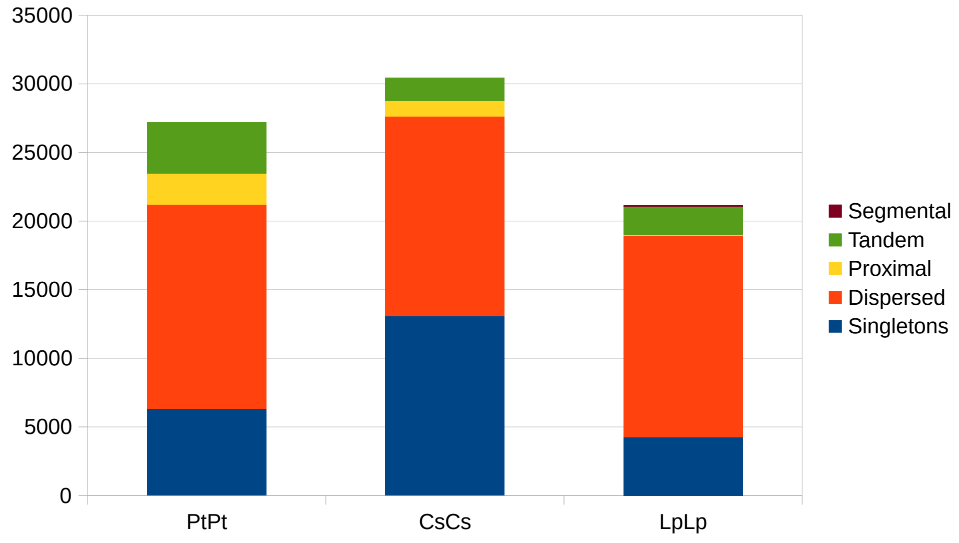

Intergenomic comparisons:

	LpCs	LpPt	CsPt
Singletons	17367	17139	23445
Dispersed	34256	31234	34193
Proximal	0	0	0
Tandem	0	0	0
Segmental	12	0	30
Total	51635	48373	57668

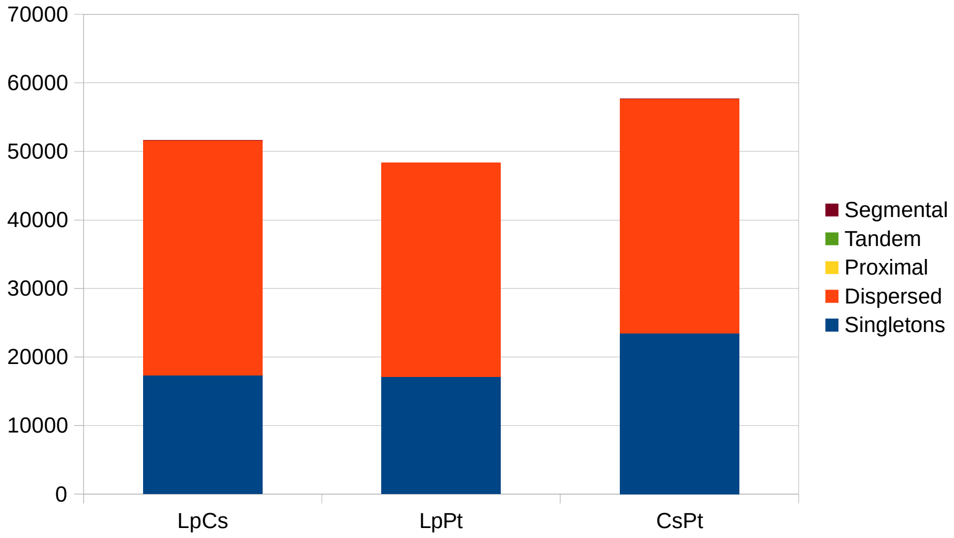

Supplement: Supplementary file 4 — Duplicated genes: five gene blocks. (PDF 36 kb) [file 12915_2017_399_MOESM4_ESM.pdf]
