# Supplementary figures and images for: The house spider genome reveals an ancient whole-genome duplication during arachnid evolution
Source: BMC Biol. 2017 Jul 31;15:62. doi: 10.1186/s12915-017-0399-x (PMC5535294; doi:10.1186/s12915-017-0399-x)

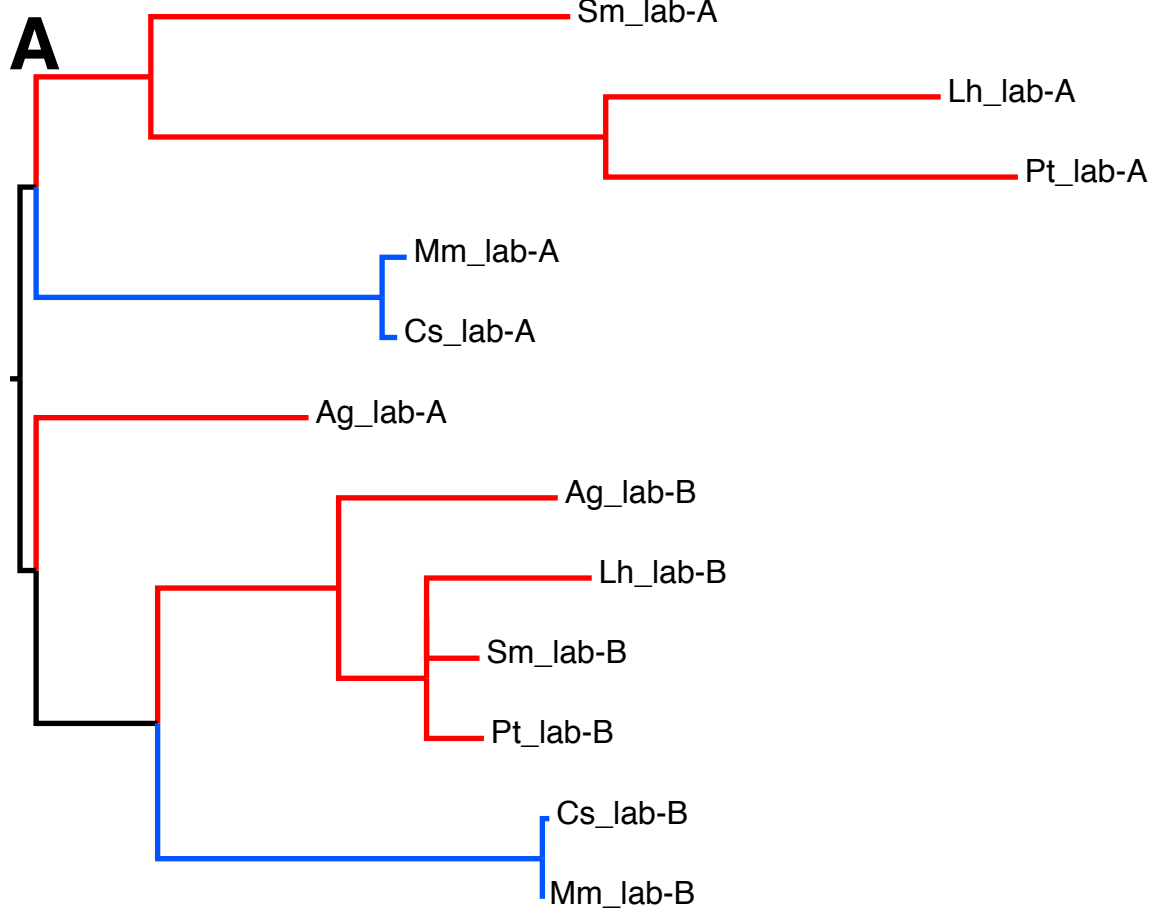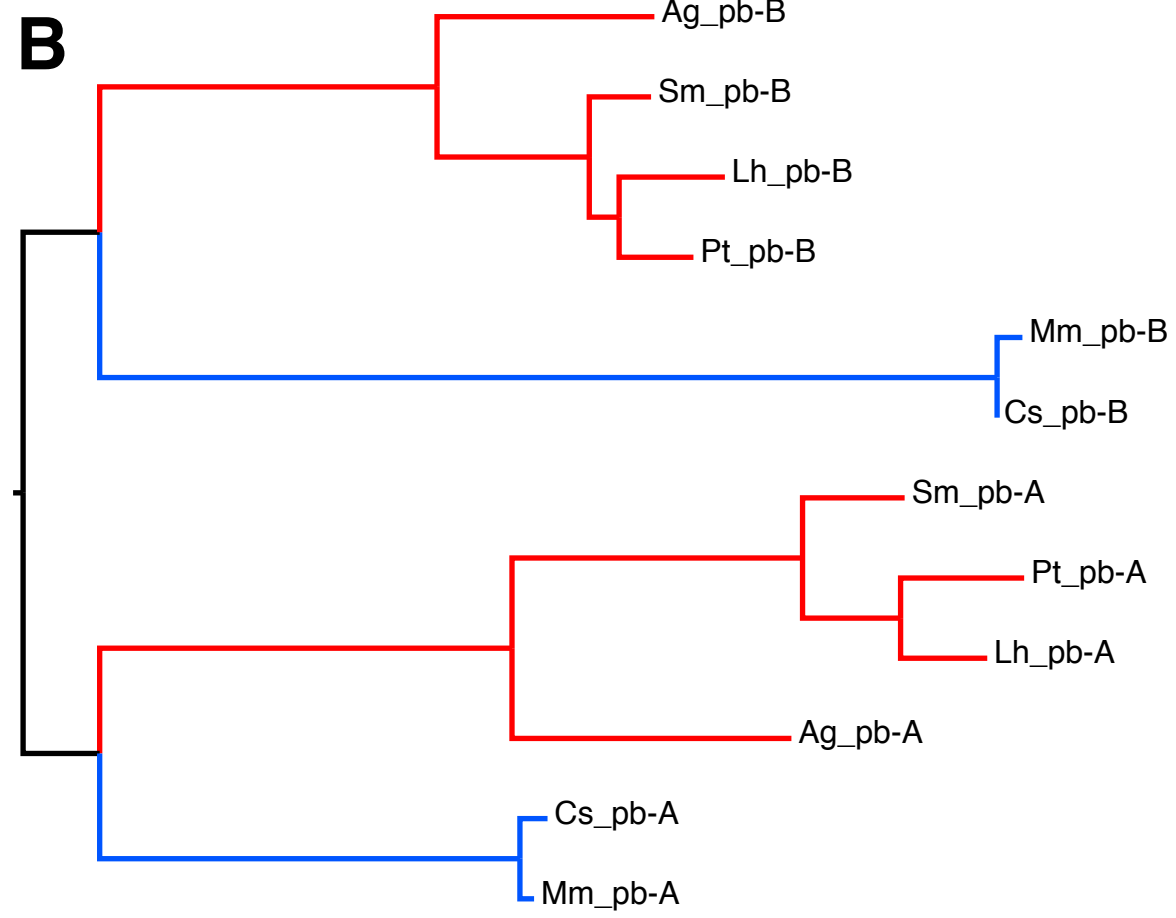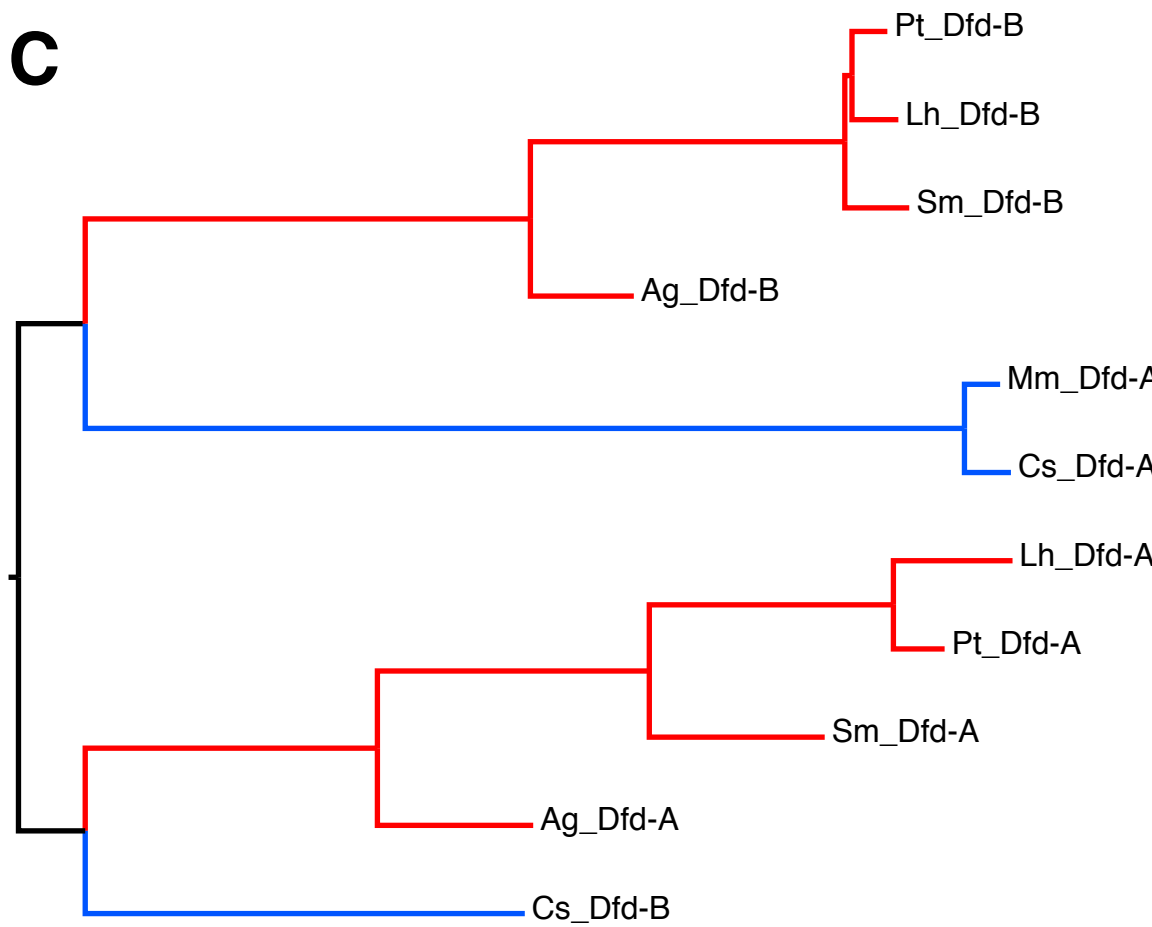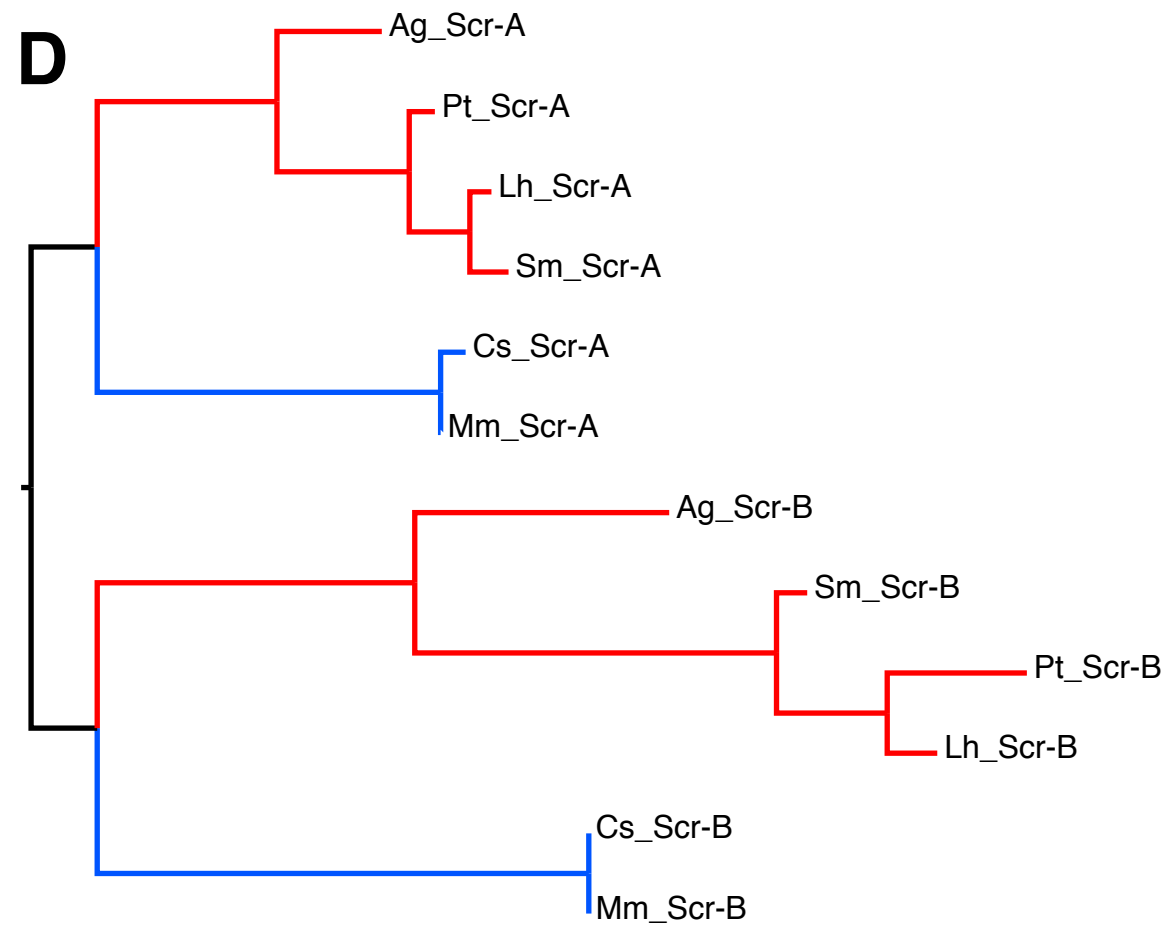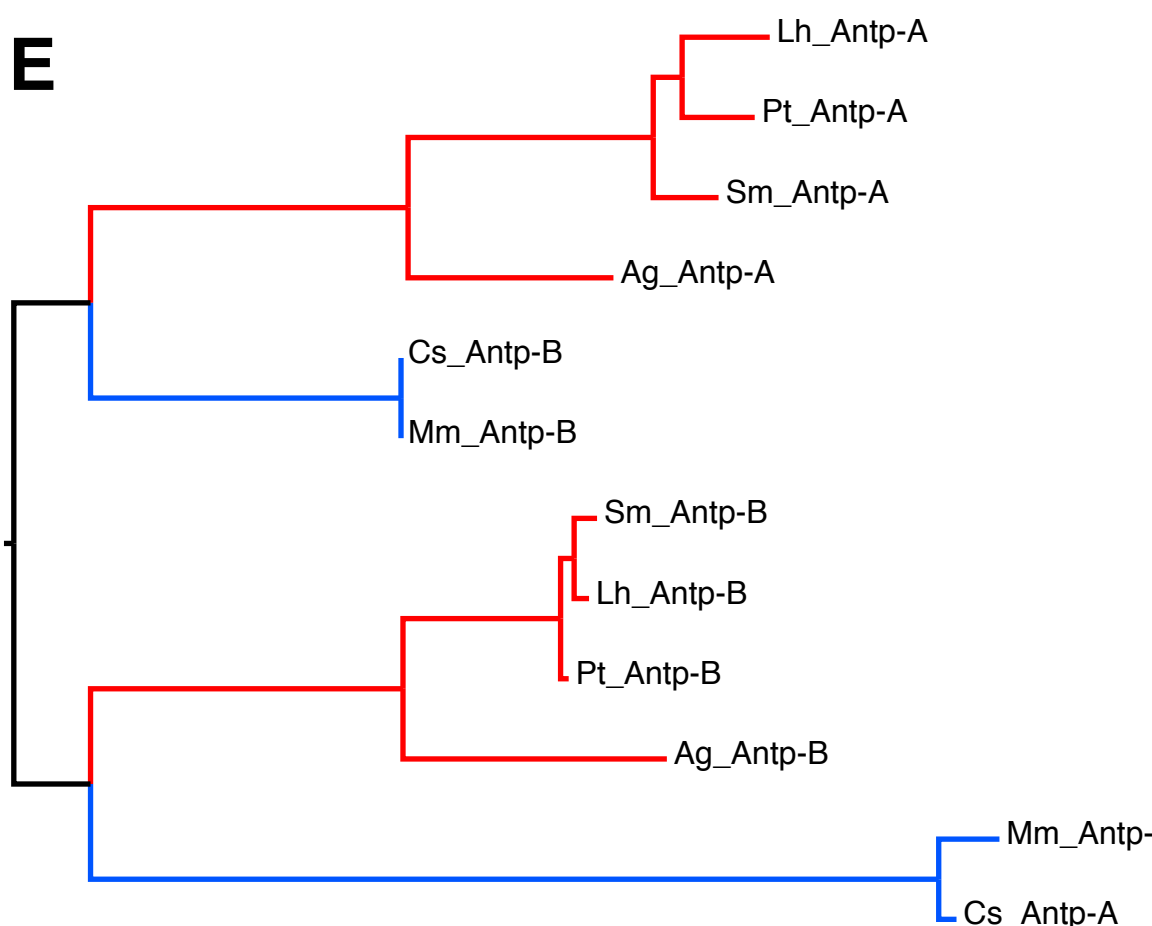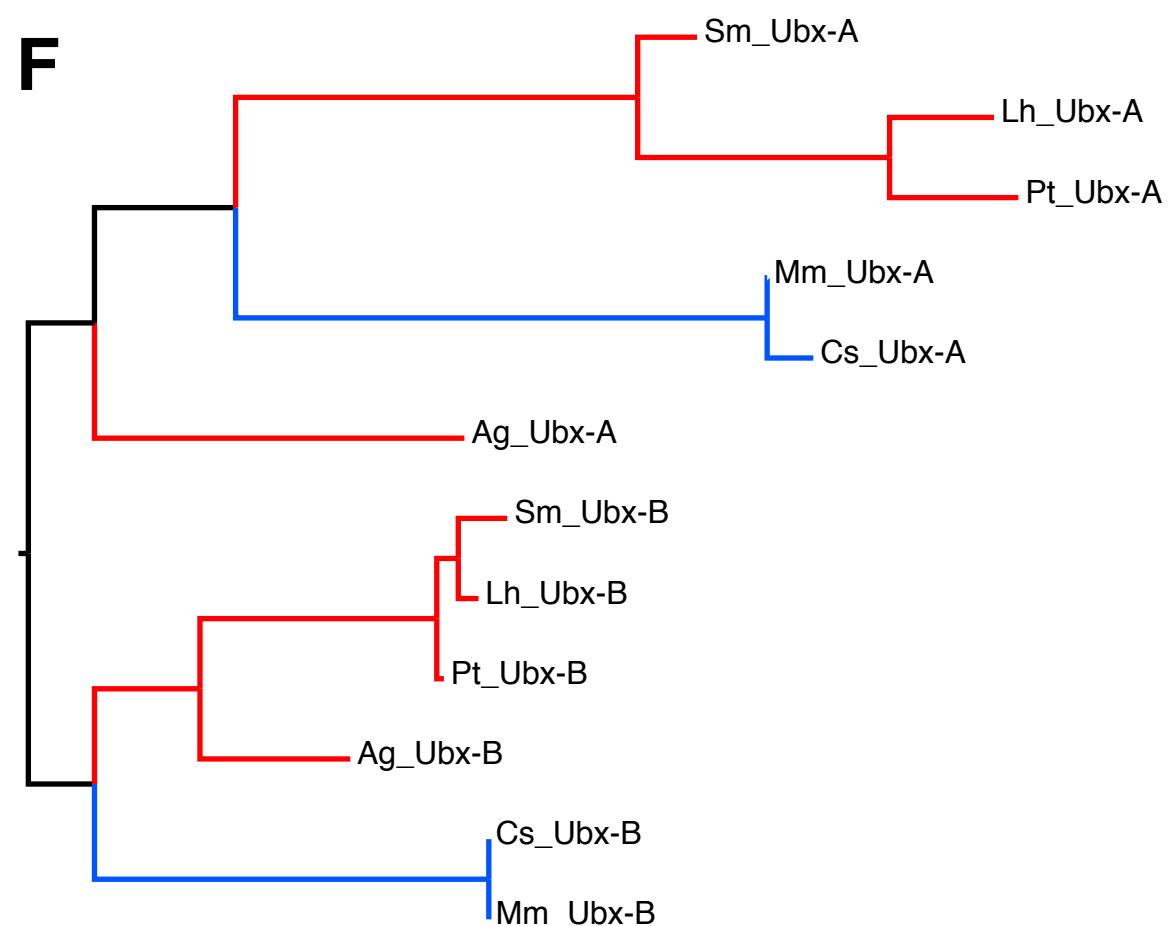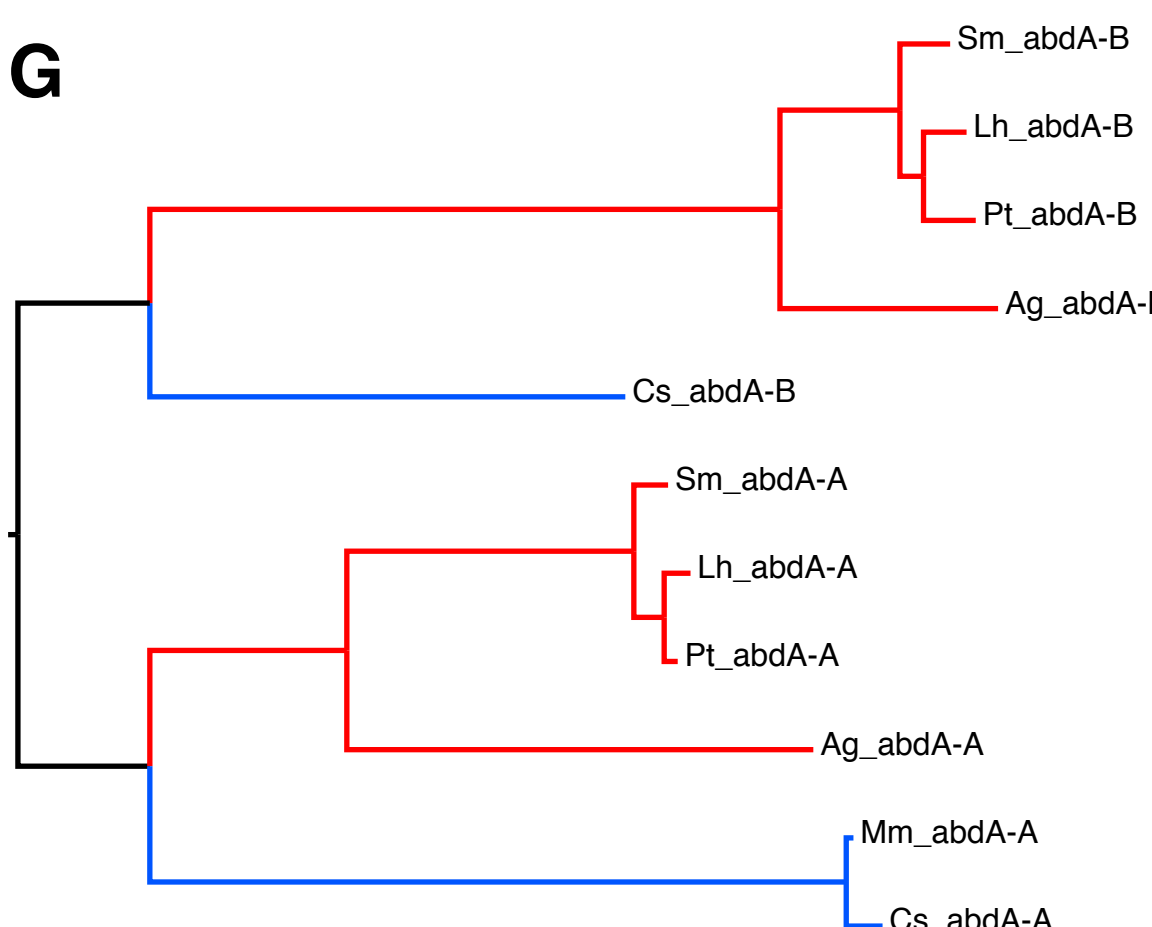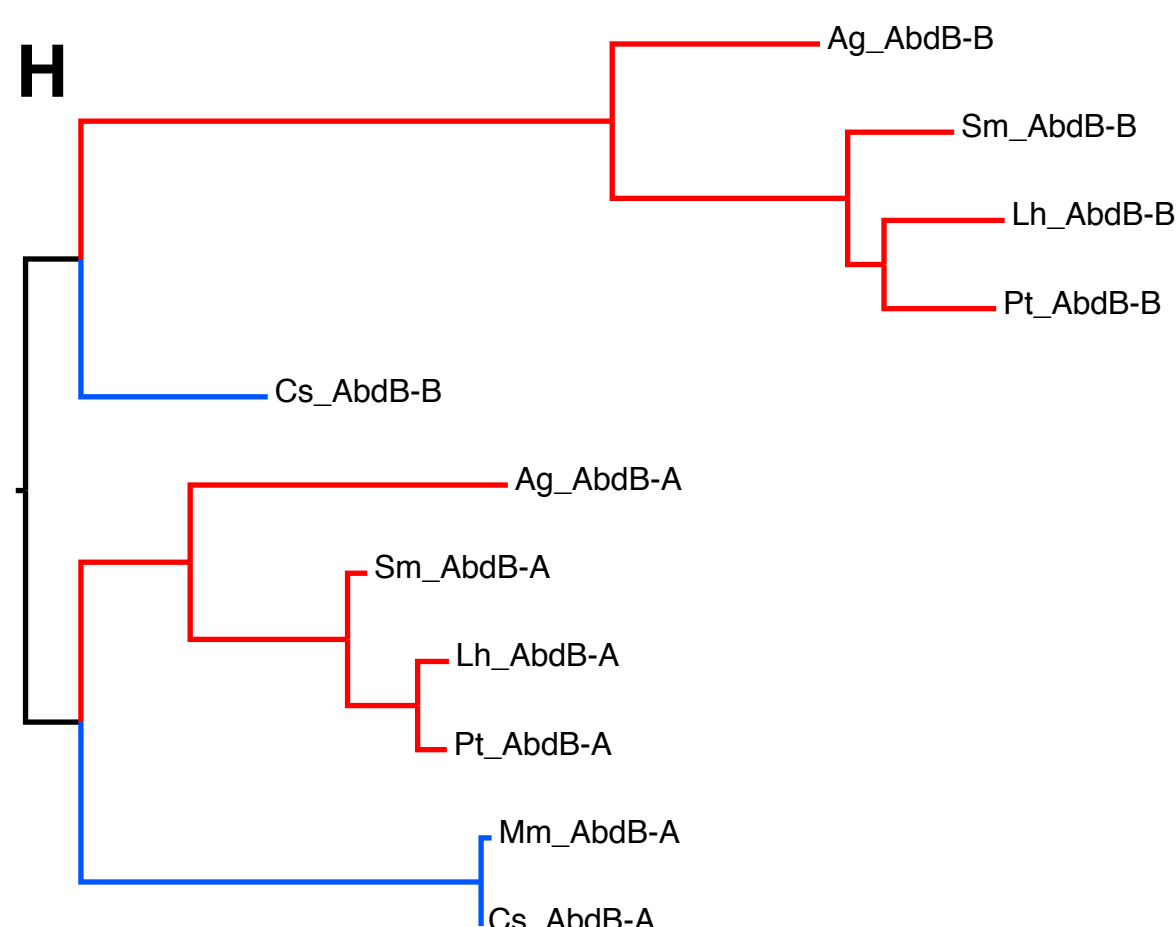

Supplement: Supplementary file 7 — Gene tree analysis of individual Hox genes. Blue branches indicate scorpion sequences; red branches indicate spider sequences. Gene trees are shown for labial (A), proboscipedia (B), Deformed (C), Sex combs reduced (D), Antennapedia (E), Ultrabithorax (F), abdominal-A, (G), and Abdominal-B (H). Log likelihood values are provided in Fig. 4. Due to insufficient sequences for hypothesis testing, gene trees were not inferred for Hox3-B and fushi tarazu. Abbreviations: Pt Parasteatoda tepidariorum, Lh Latrodectus hesperus, Sm Stegodyphus mimosarum, Ag Acanthoscurria geniculata, Cs Centruroides sculpturatus, Mm Mesobuthus martensii. (PDF 156 kb) [file 12915_2017_399_MOESM7_ESM.pdf]

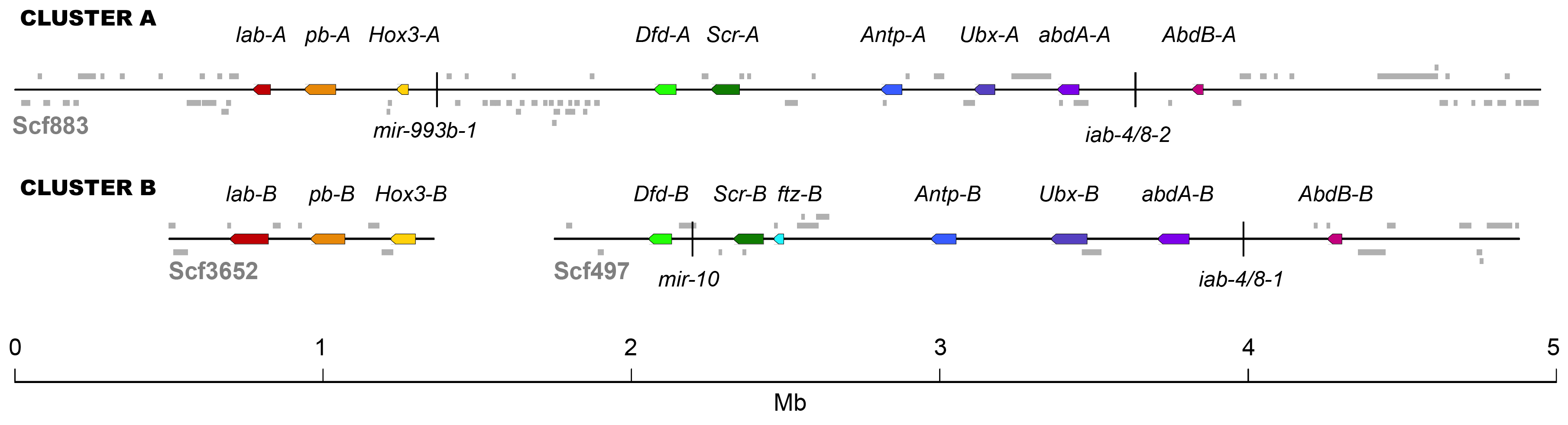

Supplement: Supplementary file 8 — P. tepidariorum Hox clusters to scale. The distances of Hox genes in clusters A and B are very similar, except for the break in cluster B. Hox genes are colored so that paralogs match, non-Hox genes are shown in grey and microRNAs are represented as lines. Genes on the positive strand and negative strand are indicated above and below the black lines, respectively. (JPG 503 kb) [file 12915_2017_399_MOESM8_ESM.jpg]

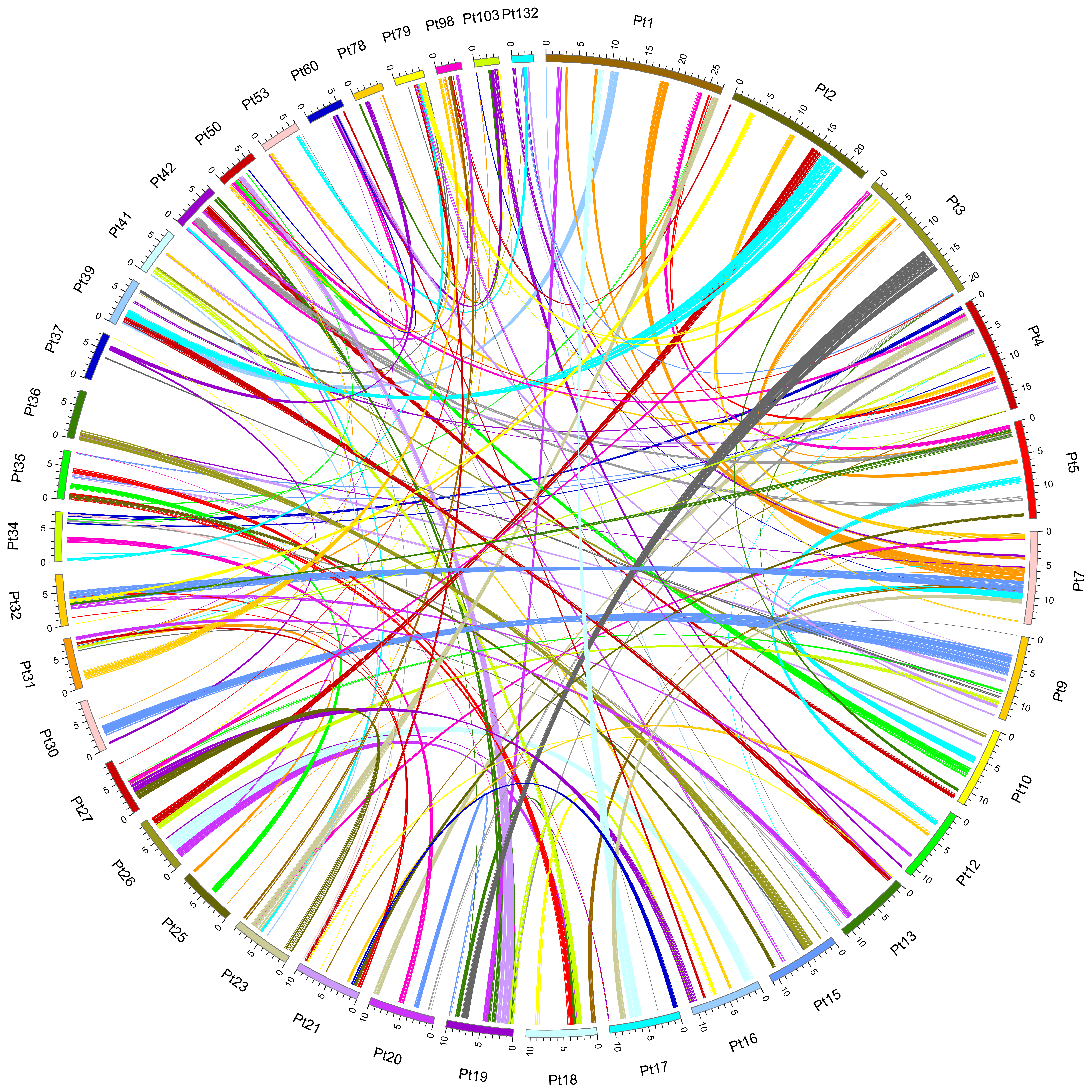

Supplement: Supplementary file 13 — Synteny of 39 P. tepidariorum scaffolds with greatest number of reciprocal hits. Circos plot of the subset of 39 scaffolds presenting the greatest numbers of hits on one another (as detected using SatsumaSynteny). One unit on the perimeter represents one Mbp. (PDF 819 kb) [file 12915_2017_399_MOESM13_ESM.pdf]

**
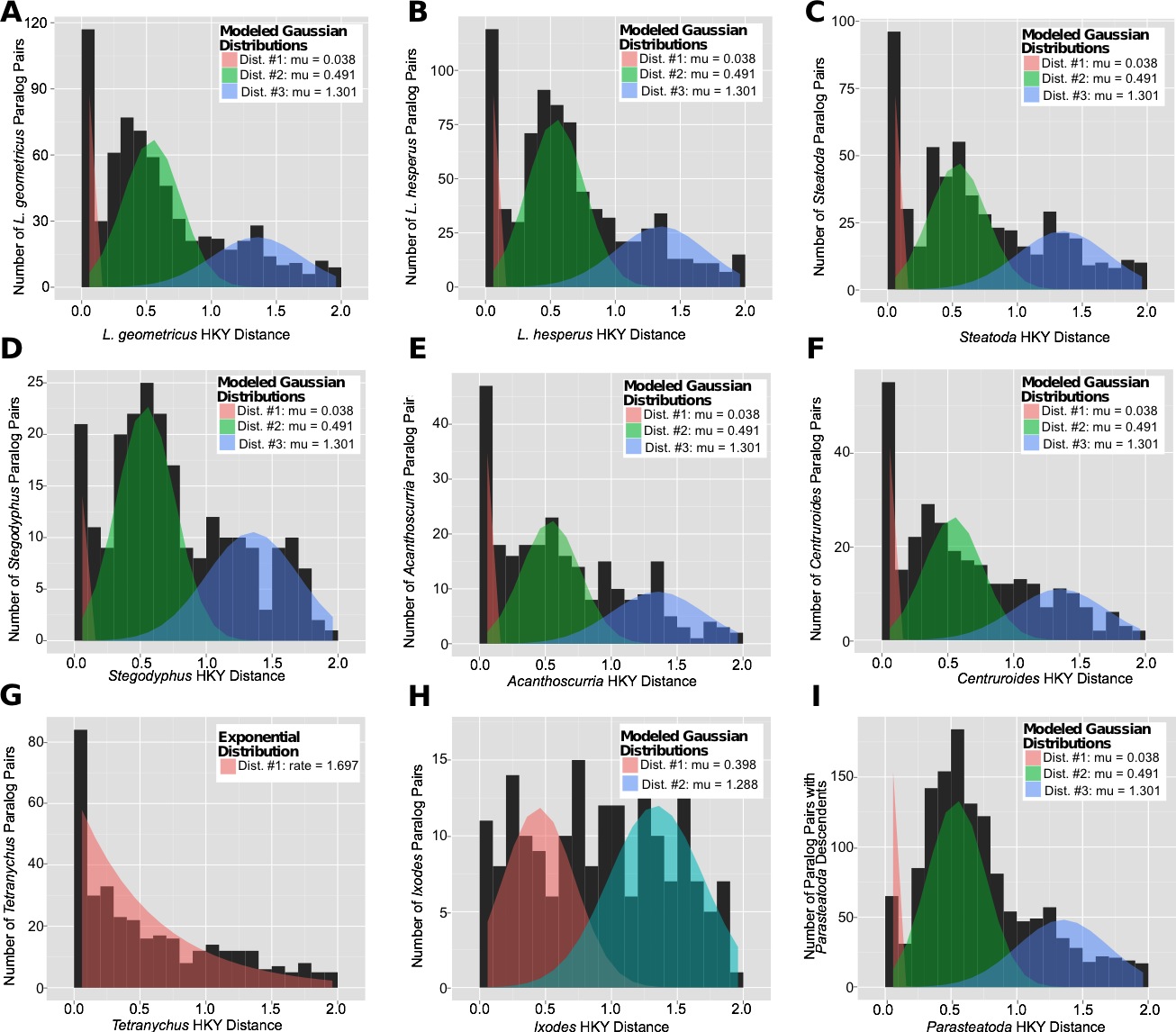
**

Supplement: Supplementary file 17 — HKY distance distributions and Gaussian mixture models of duplication nodes from P. tepidariorum-seeded gene families for eight arachnid species. The HKY distances for duplication nodes were calculated as the mean HKY branch length from the duplication node to each of the descendent genes in the species of interest (A–H). In panel (I) the distribution is for all the duplication nodes with at least one P. tepidariorum descendant, using the mean HKY distance from the node to P. tepidariorum descendants. For each panel, the best match to one of five distributions (Uniform, exponential (G), or a Gaussian mixture model with 1, 2 (H), or 3 (A–F, I) distributions) is shown. The Gaussian mixture models were seeded with Gaussian mean and standard deviations estimated from the P. tepidariorum duplication nodes (Fig. 6a). (DOCX 430 kb) [file 12915_2017_399_MOESM17_ESM.docx]

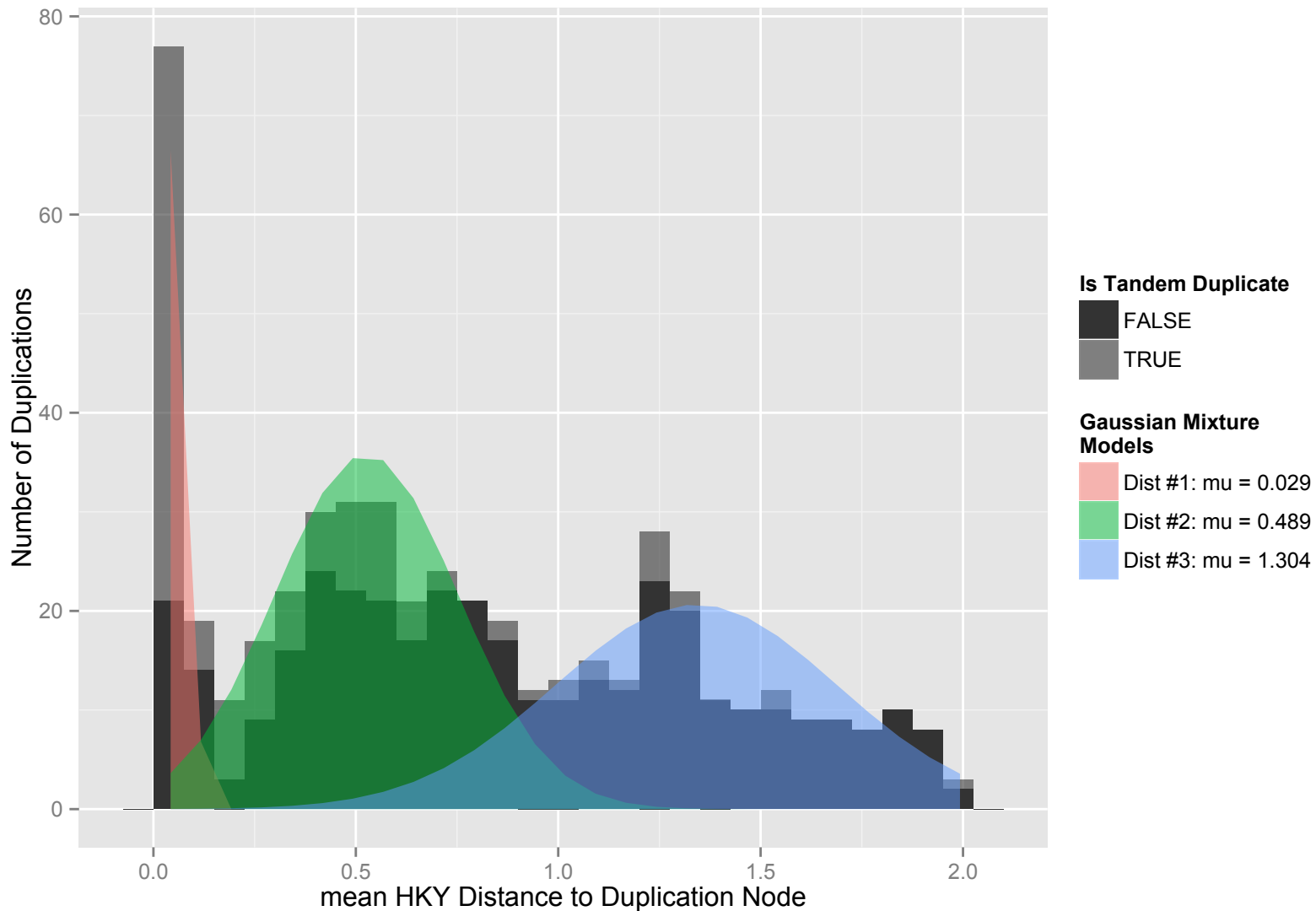

Supplement: Supplementary file 22 — Tandem duplications are abundant in young duplication events, but rare in older duplication events. HKY distance distributions and Gaussian mixture models of duplication nodes from P. tepidariorum-seeded gene families mirror those in Fig. 6a, but paralog pairs that are found in tandem (within five genes of each other on the same scaffold) are now shown in light grey, and dispersed paralogs in dark grey. (PDF 158 kb) [file 12915_2017_399_MOESM22_ESM.pdf]

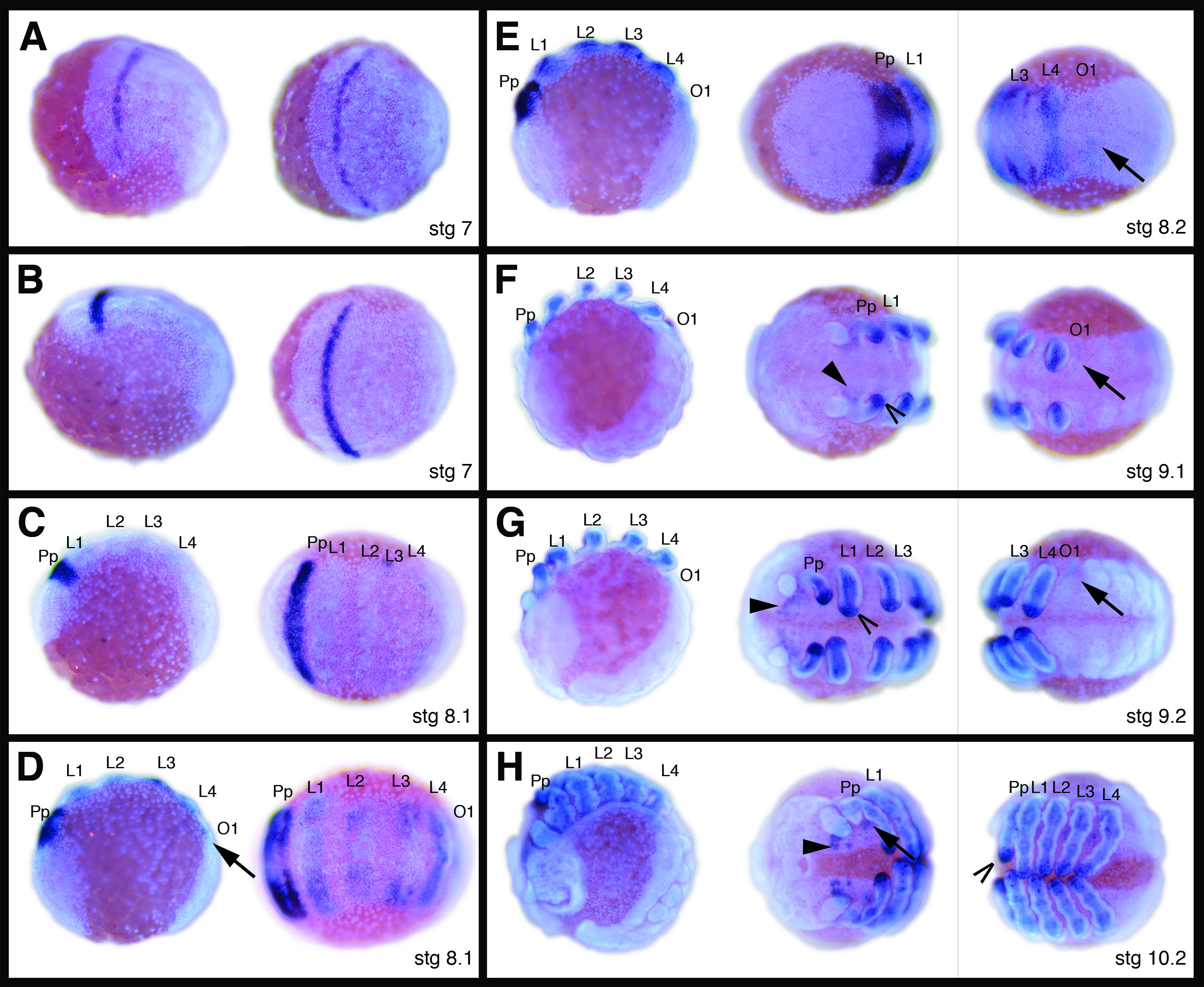

Supplement: Supplementary file 27 — P. tepidariorum proboscipedia-A expression. pb-A is first expressed in a thin stripe in the anterior of the embryo at stage 7 (A and B). The stripe broadens and as segmentation commences, it is found in the pedipalpal segment, and additionally, four weaker stripes start to appear in L1–L4 (C). The expression in the pedipalpal segment remains strongest, when by the end of stage 8.1, another stripe of weak pb-A expression is found in O1 (arrow) (D and E). During stage 9 and 10, pb-A expression is found in the mesoderm of the outgrowing pedipalp and walking legs, but also ectodermally in the distal tip of the outgrowing appendages of Pp-L4 (caret) (F–H). In the nervous system, pb-A is expressed most strongly in the Pp segment, starting at stage 9 (arrowhead), but it is also present in a smaller lateral domain of L1–O1 (arrow) (F–H). Each panel shows the same embryo, viewed laterally (left) and ventrally (right, or center and right in E–H). Anterior is to the left. Abbreviations: Pp pedipalpal segment, L walking leg segments, O opisthosomal segments. (JPG 6345 kb) [file 12915_2017_399_MOESM27_ESM.jpg]

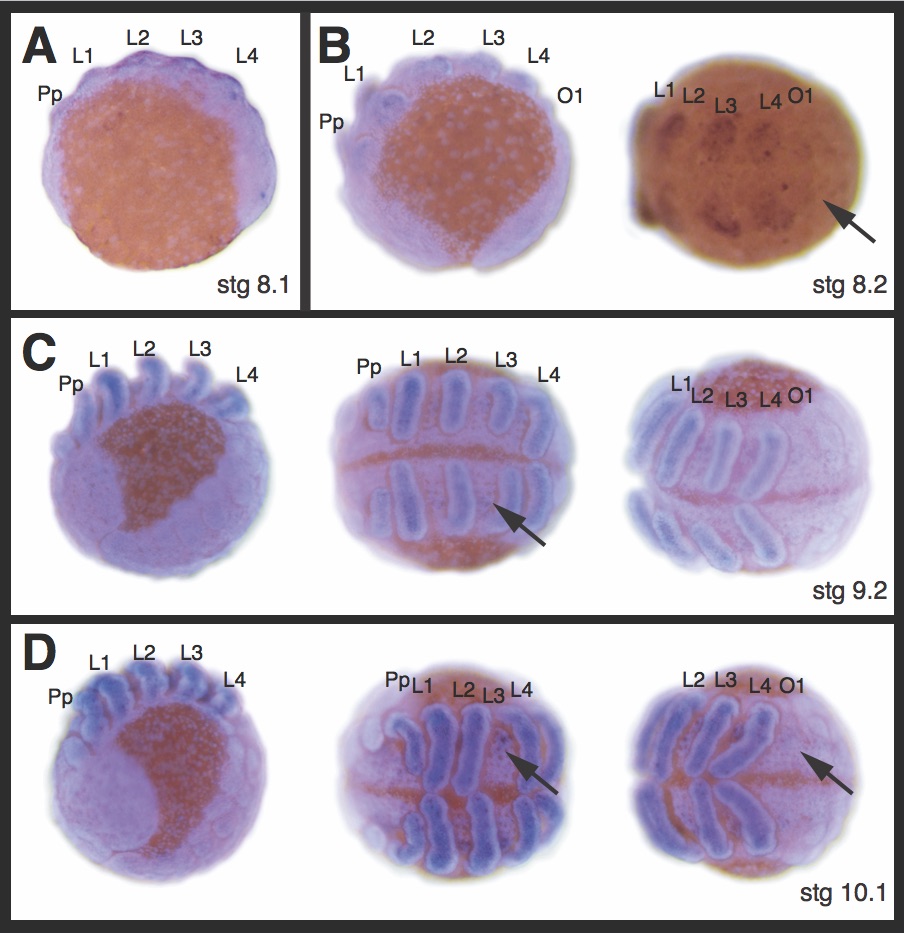

Supplement: Supplementary file 28 — P. tepidariorum proboscipedia-B expression. Weak pb-B expression emerges at stage 8 in a domain spanning from Pp to L4 (A). The weak expression is located in the mesoderm, and at stage 8.2 also visible in O1 (arrow) (B). At stages 9 and 10, pb-B is expressed in the mesoderm of the outgrowing appendages of Pp–L4, as well as in dorsolateral parts of the neuroectoderm of segments Pp–O1 (arrows) (C and D). Each panel (except A) shows the same embryo, viewed laterally (left) and ventrally (center, right). Anterior is to the left. Abbreviations: see Additional file 27: Figure S11. (JPG 143 kb) [file 12915_2017_399_MOESM28_ESM.jpg]

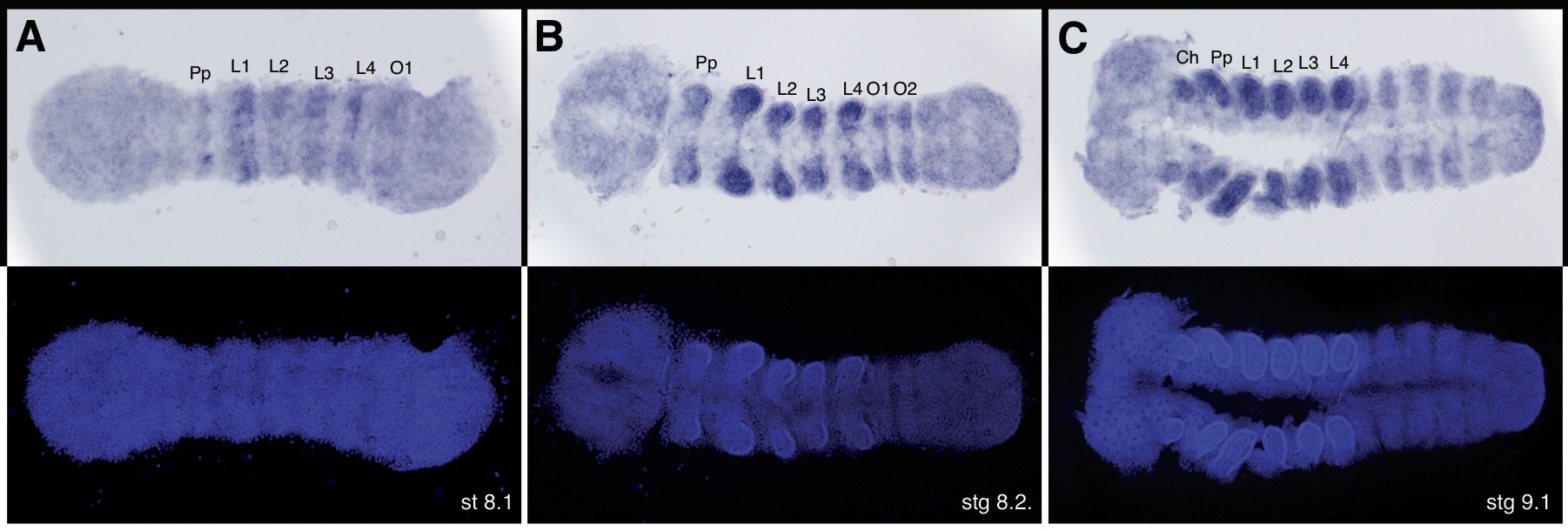

Supplement: Supplementary file 29 — P. tepidariorum Hox3-A expression. Hox3-A expression appears first at stage 7 (not shown). At stage 8.1, the expression can be found in segmental stripes in the pedipalpal and the walking leg segments, and weak expression can also be seen in O1. The strongest expression is found in the first walking leg segment (A). At stage 8.2 and 9.1, Hox3-A is expressed in the mesoderm of the developing limb buds (B, C) and in the mesoderm of posterior segments. Note that Hox3-A expression is very weak and due to the high background in the head segments as well as in the opisthosomal segments, it is difficult to define anterior/posterior boundaries. Expression of Hox3-A vanishes after stage 9 (not shown). Panels show flat-mounted embryos, brightfield image above, nuclear stain of the same embryo below. Anterior is to the left. Abbreviations: see Additional file 27: Figure S11. (JPG 214 kb) [file 12915_2017_399_MOESM29_ESM.jpg]

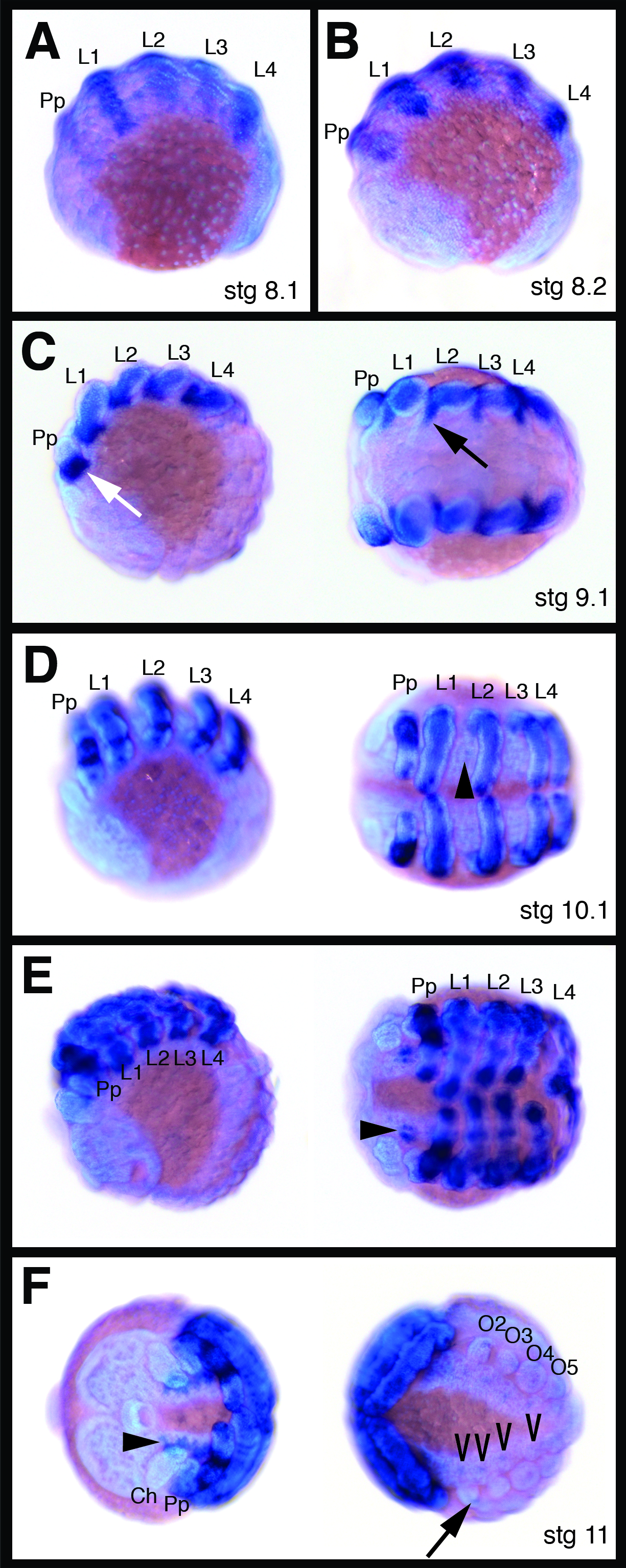

Supplement: Supplementary file 30 — P. tepidariorum Hox3-B expression. Hox3-B is not expressed before stage 8, when expression starts in broad segmental stripes from Pp to L4, most likely in the mesoderm (A). Expression is strongest in L1 and L4 (A and B). In stage 9 and 10 embryos, Hox3-B is expressed in the mesoderm of the outgrowing limb buds, except for the pedipalps, which show a broad ring of expression (white arrow in C) that later refines into rings and additional expression at the tip of the pedipalp (D–F). Additionally, expression extends anterior-ventral to the limb buds in a triangular shape at stage 9 (arrowhead in C). This expression vanishes by stage 10. Instead, Hox3-B is now also expressed in the pedipalpal and walking leg segments in segmental groups of cells in the medial neuroectoderm (arrowheads in D–F). In stage 11 embryos, Hox3-B is additionally expressed in dots in the ventral neuroectoderm of every opisthosomal segment (carets in F). Furthermore, a dot of expression can be found in the first opisthosomal limb bud (arrow in F). Embryos in A and B are shown laterally, embryos C–E are shown laterally on the left and ventrally on the right. F shows ventral views of the head region (left) and the opisthosoma (right) of an embryo at a similar stage as the embryo in E. Anterior is to the left. Abbreviations: see Additional file 27: Figure S11. (JPG 4203 kb) [file 12915_2017_399_MOESM30_ESM.jpg]

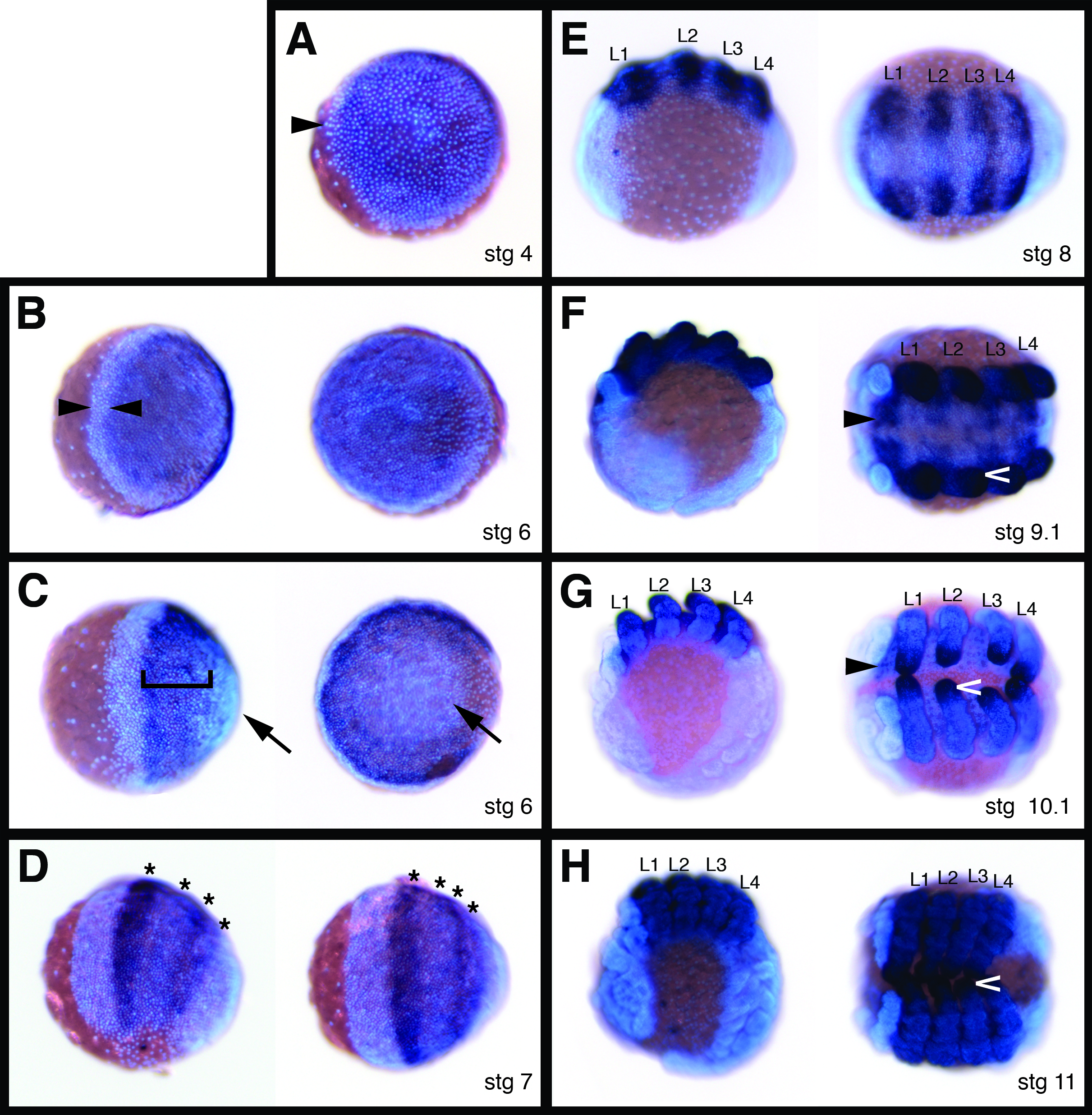

Supplement: Supplementary file 31 — P. tepidariorum Deformed-A expression. Dfd-A is first expressed at stage 4, in almost all cells but the rim (arrowhead) of the germ disc (A). During stage 6, cells not expressing Dfd-A at the outer rim, the future anterior, have multiplied (arrowheads) (B). Later during stage 6, the expression clears from the posterior (former center of the germ disc, arrow) and the expression starts to form a broad stripe (bracket) (C). This uniform stripe of expression first subdivides into two stripes with lower expression between these stripes during stage 7. Then, the posterior domain splits into two more stripes and, in between the anterior and posterior domains, a new stripe gets inserted such that there are now four stripes of Dfd-A expression (asterisks) (D). These four stripes are later located in the four walking leg segments at stage 8 (E). In stage 9–11 embryos, Dfd-A is strongly expressed in the outgrowing walking legs with strongest expression in the tips of the legs (white carets) (F–H) and in the neuroectoderm (arrowheads in G and H). Each panel (except for A, B, C) shows the same embryo, viewed laterally (left) and ventrally (right). Anterior is to the left in all panels. Abbreviations: See Additional file 27: Figure S11. (JPG 6387 kb) [file 12915_2017_399_MOESM31_ESM.jpg]

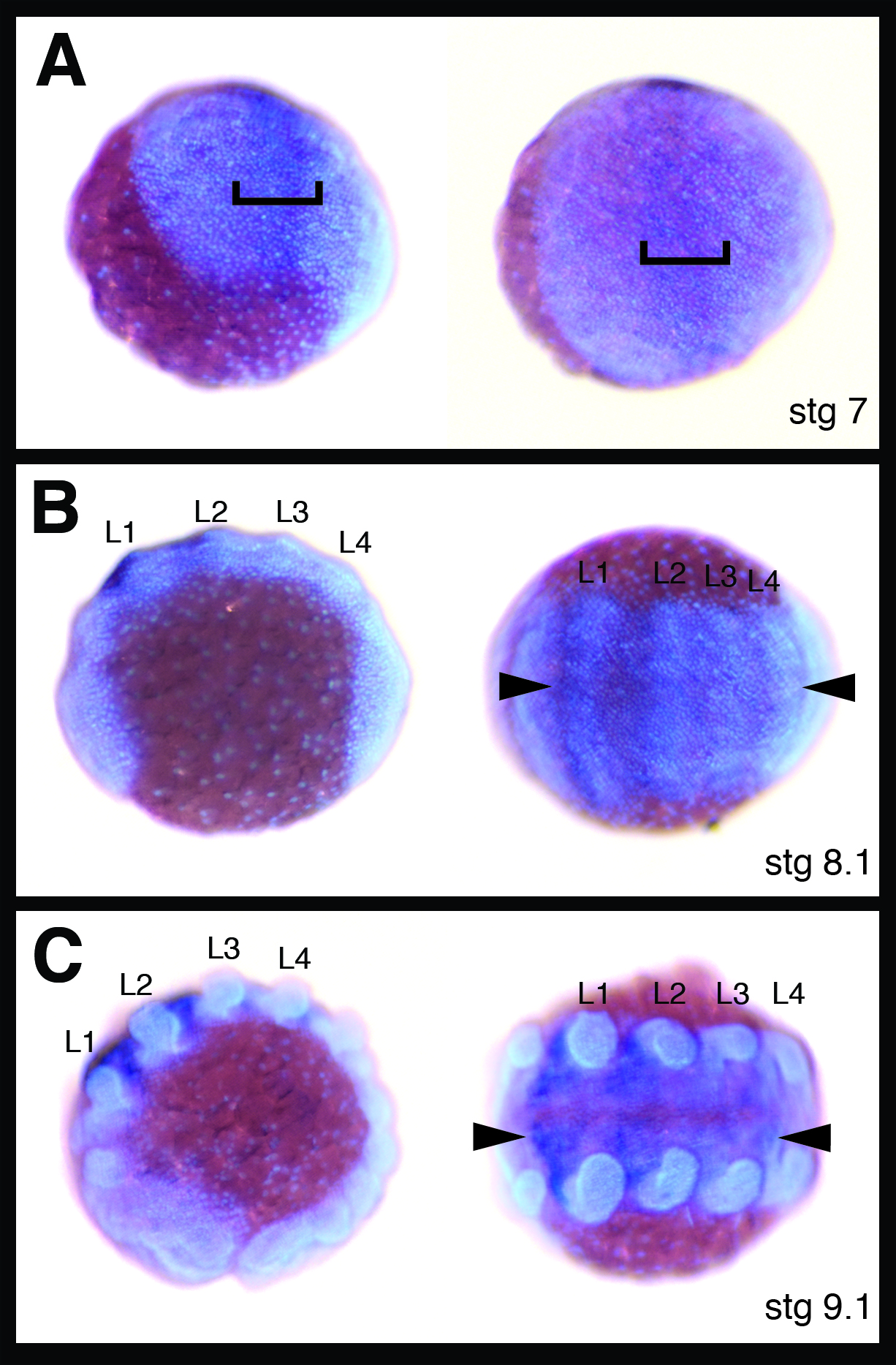

Supplement: Supplementary file 32 — P. tepidariorum Deformed-B expression. Dfd-B expression is first detected in a weak, broad stripe in the developing germ band (bracket) (A). This stripe can be allocated to the first two walking leg segments at stage 8 (B). The anterior of the segments is stained stronger than the posterior. Additional, but much weaker expression can be seen in the L3 and L4 segments. At stage 9, Dfd-B is predominantly expressed in the ventral neuroectoderm (C). The anterior and posterior expression domain is marked by arrowheads in B and C, respectively. Each panel shows the same embryo viewed laterally (left) and ventrally (right). Anterior is to the left. Abbreviations: See Additional file 27: Figure S11. (JPG 2272 kb) [file 12915_2017_399_MOESM32_ESM.jpg]

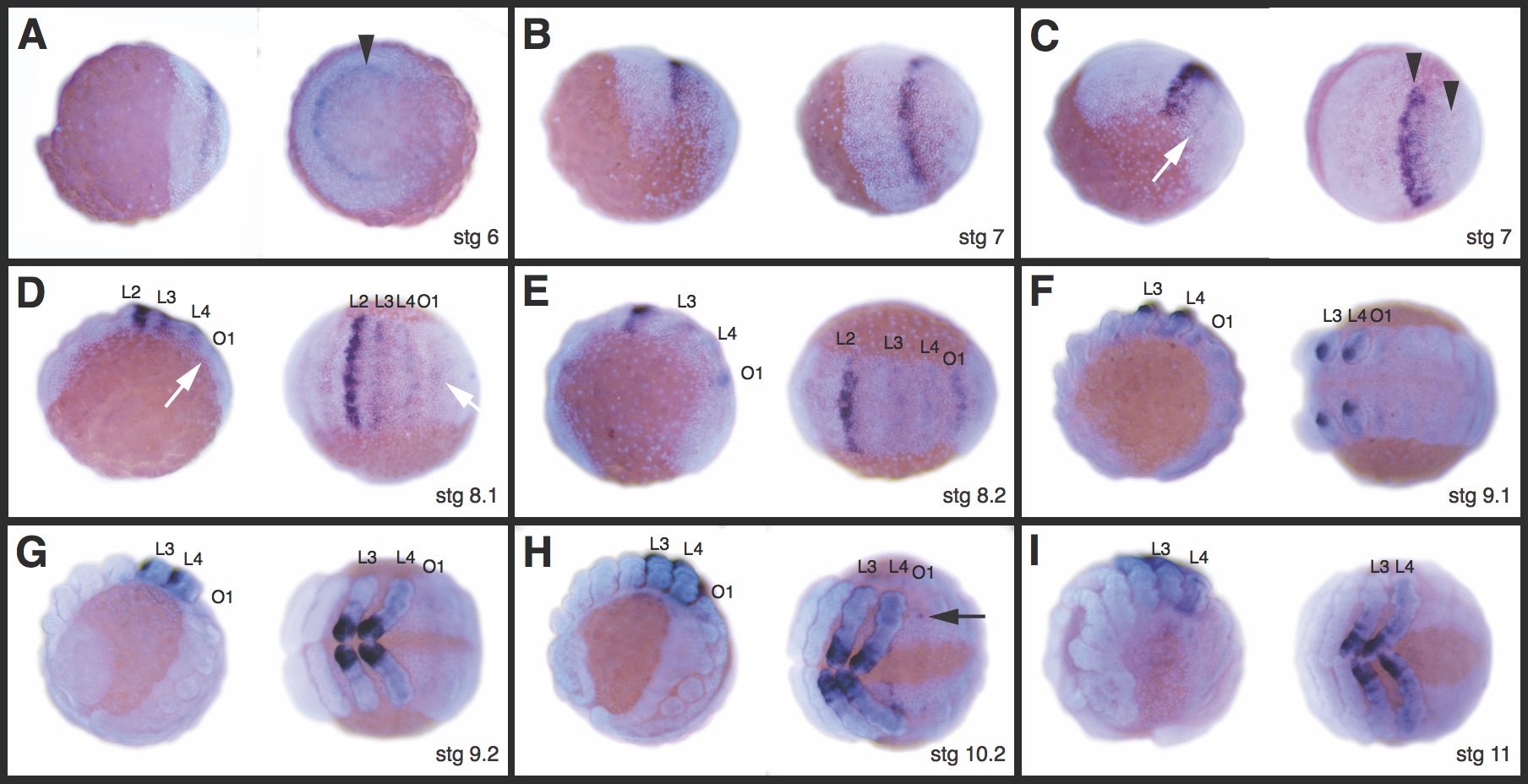

Supplement: Supplementary file 33 — P. tepidariorum Sex combs reduced-A expression. A stripe of Scr-A expression first appears at stage 6 (A). The stripe broadens during stage 7 (B), at the end of which it starts to split into two stripes, and a third new stripe appears posterior to the initial stripes (borders of expression marked by arrowheads) (C). At stage 8.1, there are four stripes located in the posterior parts of L2, L3, and L4 and the newest stripe appears in O1 (white arrow) (D and E). In limb bud stages, only the L3 and L4 limb buds carry Scr-A expression, mostly in their distal tips. Expression in L2 is restricted to the neuroectoderm (not shown), and expression in O1 is restricted to the posterior part of each hemisegment as well (F). At later stages, Scr-A expression in L3 and L4 refines to several rings in the distal part of the legs. Expression in L2 becomes undetectable. (G–I). At stage 10.1, Scr-A is visible in a neuroectodermal patch in L4 and a dot in O1 (arrow) (H). Each panel shows the same embryo viewed laterally (left) and ventrally (center and right). Anterior is to the left. Abbreviations: See Additional file 27: Figure S11. (JPG 266 kb) [file 12915_2017_399_MOESM33_ESM.jpg]

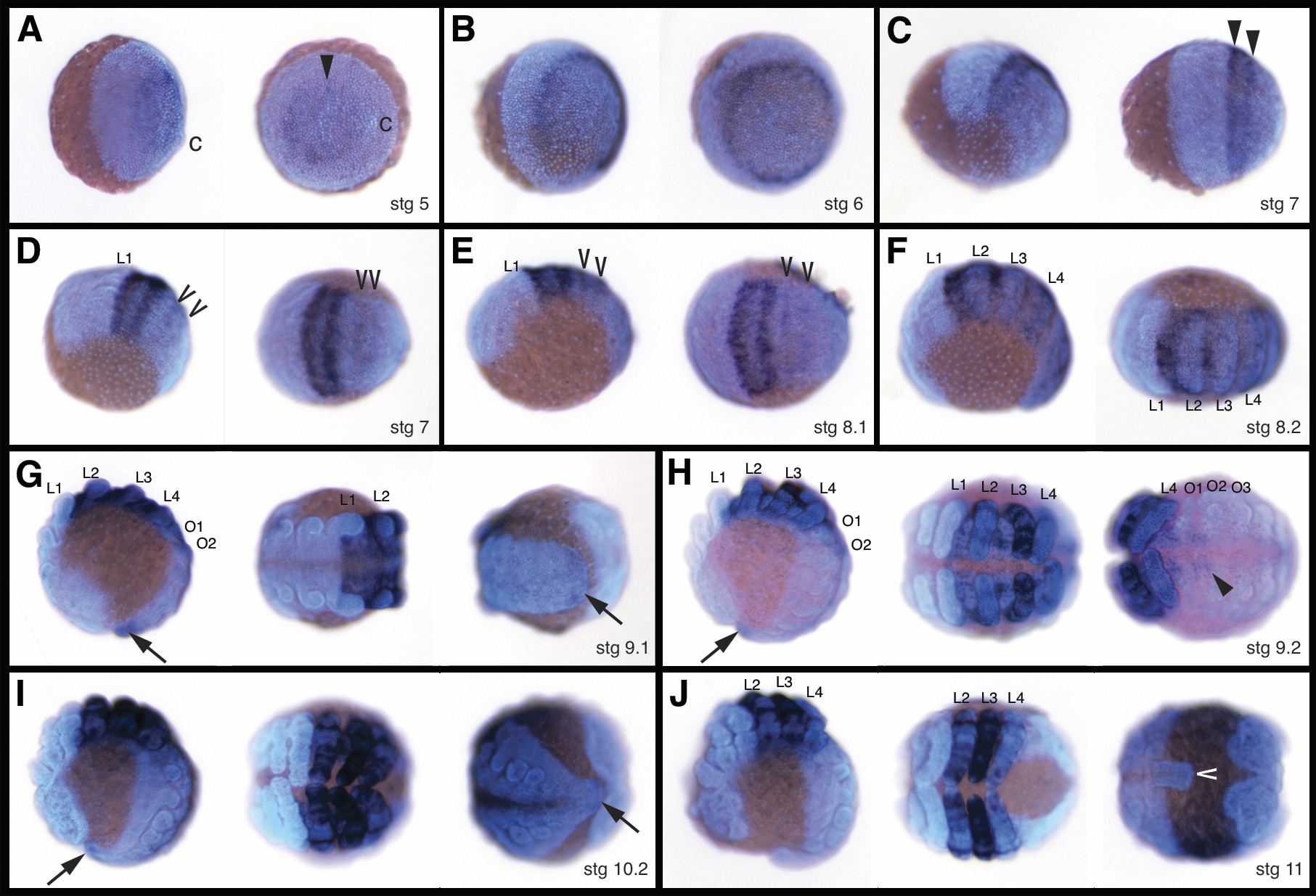

Supplement: Supplementary file 34 — P. tepidariorum Sex combs reduced-B expression. Scr-B is first expressed in a cap-like domain (arrowhead) in the center of the germ disc at stage 5. The position of the cumulus is marked by a ‘c’ (A). The expression widens during stage 6. Scr-B now forms an open ring, localized roughly halfway between anterior rim of the opening germ disc and the future posterior center of the germ disc. The cells posterior to the ring also express Scr-B, but at a much lower level (B). At stage 7, the ring splits up into two stripes (arrowheads) (C) and, later, two new stripes appear (carets) (D). The most anterior stripe of expression lies between L1 and L2 at stage 8, the second anterior stripe lies between L2 and L3. The two posterior stripes cover L3 and L4. The weak expression of Scr-B continues posterior to these stripes (E and F). Scr-B is predominantly expressed in the limb buds and the ventral neuroectoderm of stage 9.1 embryos (G). The anterior expression border is directly posterior to the L1 limb bud. Expression of Scr-B continues in the opisthosoma, but is much weaker, except for a domain in the SAZ (arrow) (G). This expression in the SAZ continues to the end of segmentation (H and I). No more expression is visible at the posterior end of the embryo at stage 11 (white caret) (J). Scr-B expression forms multiple rings in the legs (H–J), but it is much more strongly expressed in L3 compared to L2 and L4. At late stage 9, the expression of Scr-B in the neuroectoderm can also be found in every segment of the opisthosoma (arrowhead in H). Each panel shows the same embryo viewed laterally (left) and ventrally (center and right). Anterior is to the left. Abbreviations: See Additional file 27: Figure S11. (JPG 393 kb) [file 12915_2017_399_MOESM34_ESM.jpg]

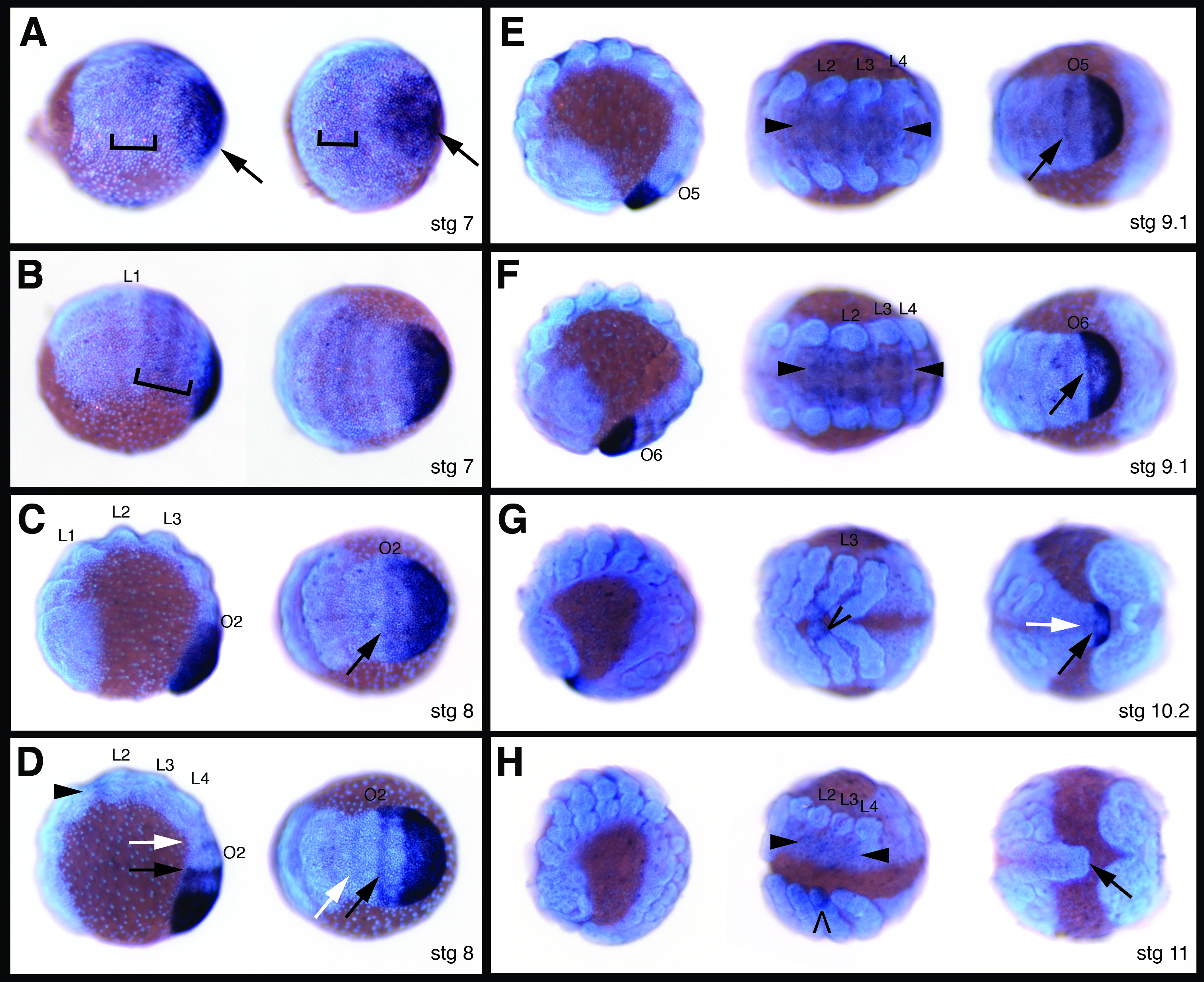

Supplement: Supplementary file 35 — P. tepidariorum fushi tarazu expression. ftz is first expressed at stage 6 in a semicircle at the posterior end where the SAZ is forming (arrow) (A). The bracket marks an anterior broad stripe of ftz expression. This domain of expression gets weaker during stages 7 and 8, where mostly only the anterior border of the domain anterior to L2 (arrowhead in D) is visible. The expression is almost invisible when it is viewed ventrally. The expression in the SAZ persists, and does not clear from the posterior end (A–G). It emanates stripes at the anterior end of the SAZ (arrow in C–G). Stripes stay visible at the anterior border of the last formed segment (white arrow in D and G) until the next stripe of ftz expression forms. Only after segmentation finishes does the expression at the posterior disappear (arrow in H). Meanwhile, the anterior border of the Hox expression domain stretches from L2 to L4 and is predominantly found ventrally (black arrowheads in F and H). The legs on one side of a stage 11 embryo were removed to show the ventral Hox domain, which has concentrated to one spot per hemisegment (black arrowheads) within the L2–L4 domain (H). At stage 10, ftz expression appears in a ring near the distal tip of L3 (carets in G–H). Each panel shows the same embryo, viewed laterally (left) and ventrally (right, or center and right in E–H). Anterior is to the left. Abbreviations: See Additional file 27: Figure S11. (JPG 7681 kb) [file 12915_2017_399_MOESM35_ESM.jpg]

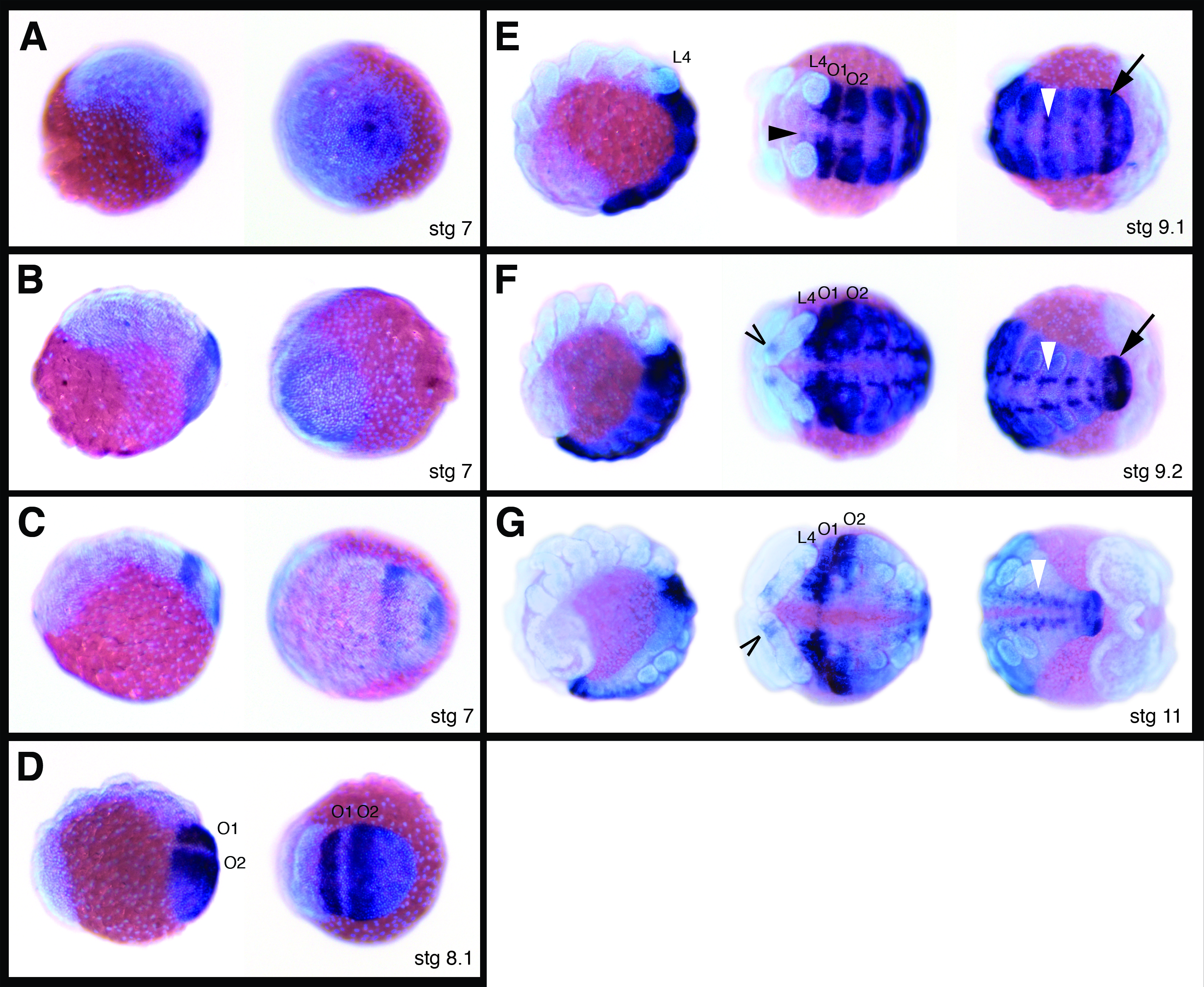

Supplement: Supplementary file 36 — P. tepidariorum Antennapedia-A expression. Antp-A expression first develops in the SAZ at stage 7 (A). This expression transforms into a stripe and is followed by cells that show only weak expression of Antp-A (B), before new expression appears at the posterior end of the SAZ (C). The first two stripes can be allocated to the first two opisthosomal segments at stage 8 (D). New stripes keep appearing from the SAZ (arrows, E–H) until the end of segmentation (G). At stage 9, the anterior border of Antp-A expression reaches into the posterior half of L4 (arrowhead, E). Expression is now also seen in the ventral neuroectoderm, where it forms longitudinal rows in each hemisegment (white arrowheads, E–G), and rings in the L4 appendage (caret, F and G). Throughout development, Antp-A expression is always strongest in O1 and the anterior half of O2. Each panel shows the same embryo, viewed laterally (left) and ventrally (right, or center and right in E–G). Anterior is to the left. Abbreviations: See Additional file 27: Figure S11. (JPG 6657 kb) [file 12915_2017_399_MOESM36_ESM.jpg]

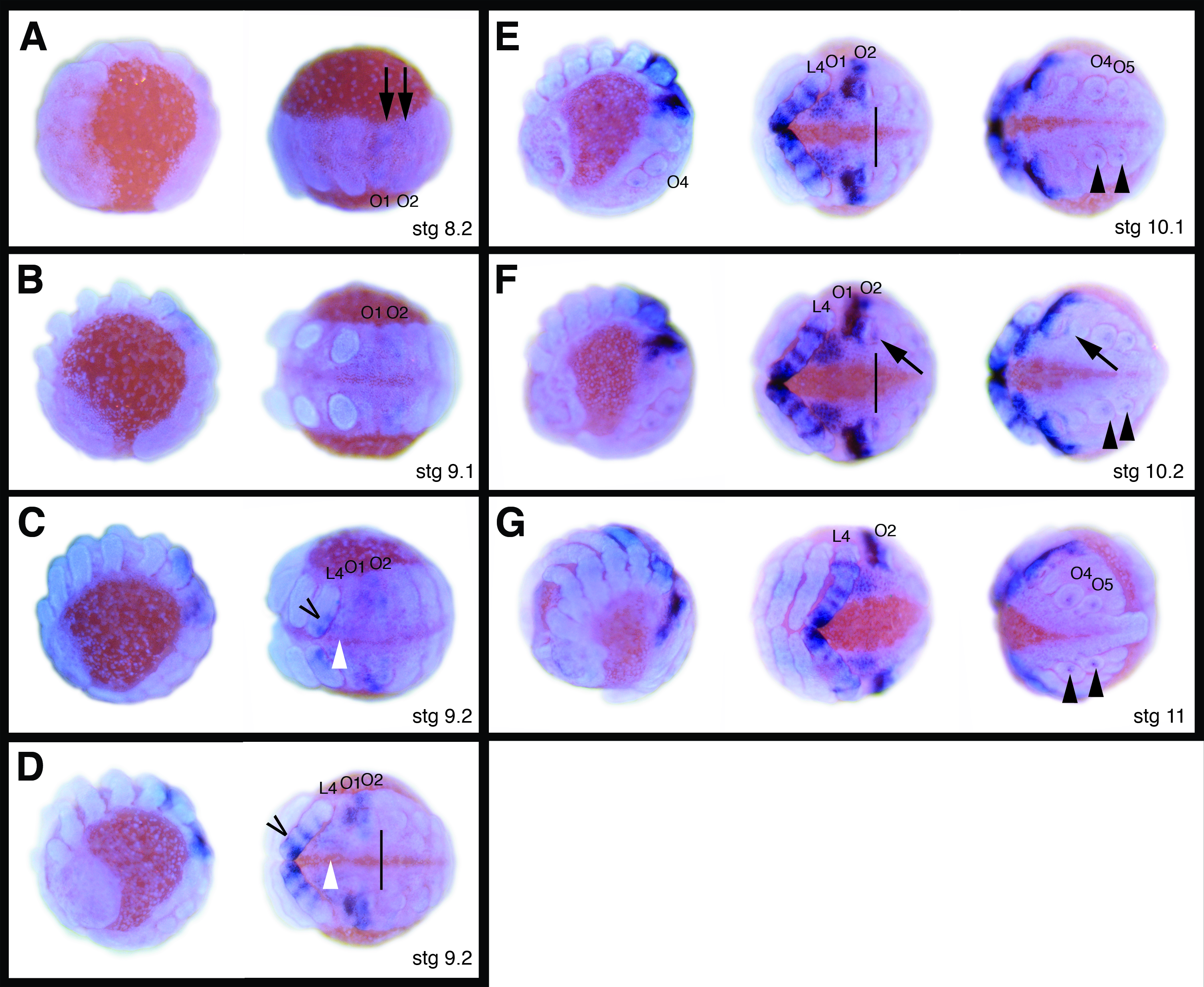

Supplement: Supplementary file 37 — P. tepidariorum Antennapedia-B expression. Antp-B first emerges in the first two opisthosomal segments at stage 8.2 (arrows) (A). The Antp-B domain expands anteriorly into the posterior part of L4 during stage 9 (white arrowheads) (B–D). Additionally, Antp-B forms rings of expression in the L4 appendage (carets in C and D). While initially the very posterior part of O2 is free of Antp-B expression (the O2/O3 border is demarcated by a black vertical line in D–F), during stage 10, expression can also be found at the O2/O3 border (arrows) (F). Furthermore, during stage 10, the expression pattern refines, in that the L4 and O1 Antp-B expression is mostly in the ventral neuroectoderm and not found dorsally, while the O2 expression is excluded from the most ventral region (E–G). Starting at stage 10, Antp-B is also expressed in one dot each in the opisthosomal limb buds on O4 and O5 (arrowheads) (E–G). Each panel shows the same embryo, viewed laterally (left) and ventrally (right, or center and right in E–G). Anterior is to the left. Abbreviations: See Additional file 27: Figure S11. (JPG 5223 kb) [file 12915_2017_399_MOESM37_ESM.jpg]

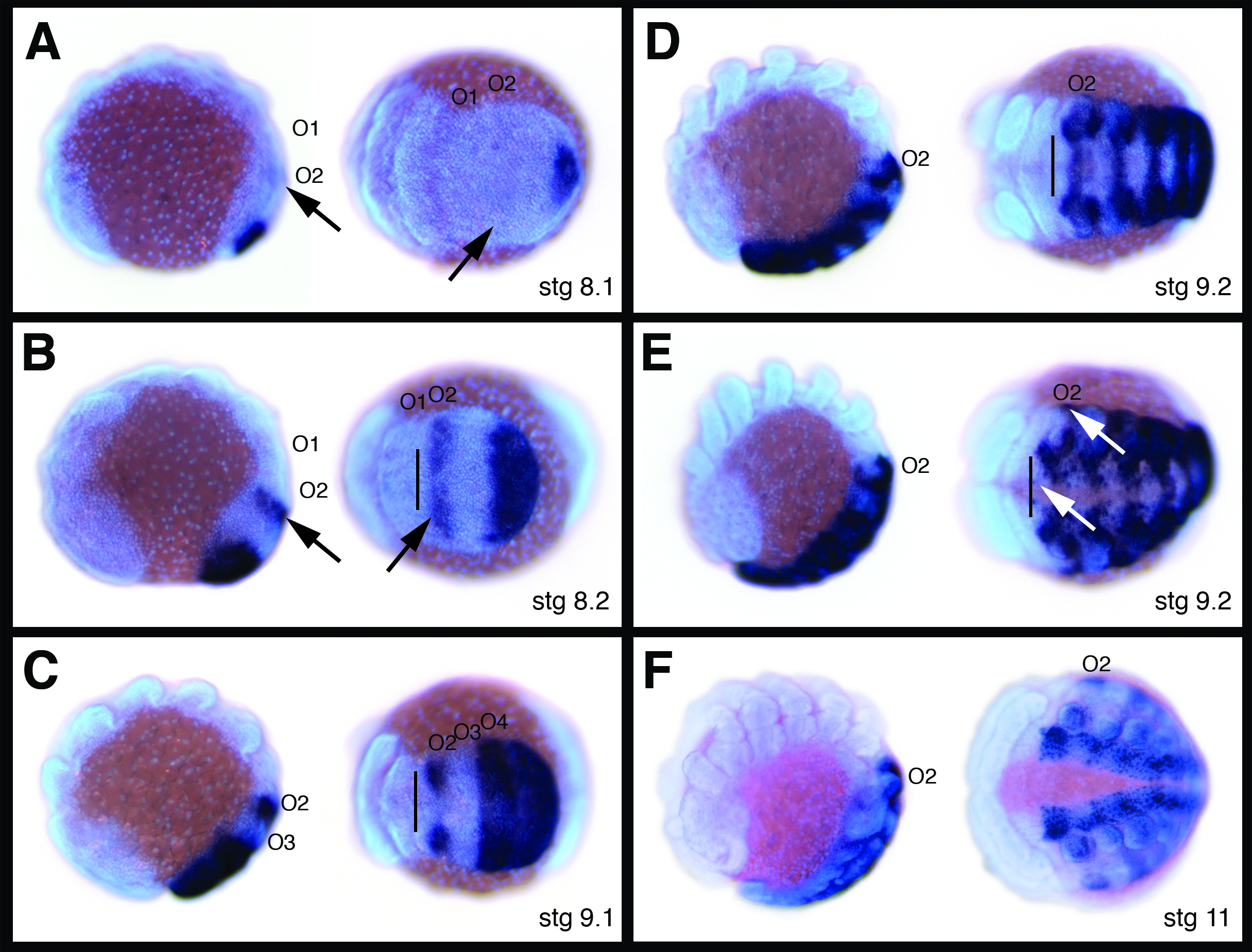

Supplement: Supplementary file 38 — P. tepidariorum Ultrabithorax-A expression. Ubx-A is first expressed at stage 8.1 in the SAZ and in a weak stripe anterior to that (arrow) (A). This stripe gets stronger and the posterior domain enlarges (B and C) so that all new tissue added posteriorly expresses Ubx-A at equal levels (D–F). The anterior border is located in O2, while the very anterior part of O2 initially does not express Ubx-A (segmental boundaries in C–F indicated by black vertical lines) until the end of stage 9.2, when it is expressed dorsally and in the neuroectoderm also in the anterior part of O2 (white arrow in E). Each panel shows the same embryo, viewed laterally (left) and ventrally (right). Anterior is to the left. Abbreviations: See Additional file 27: Figure S11. (JPG 4678 kb) [file 12915_2017_399_MOESM38_ESM.jpg]

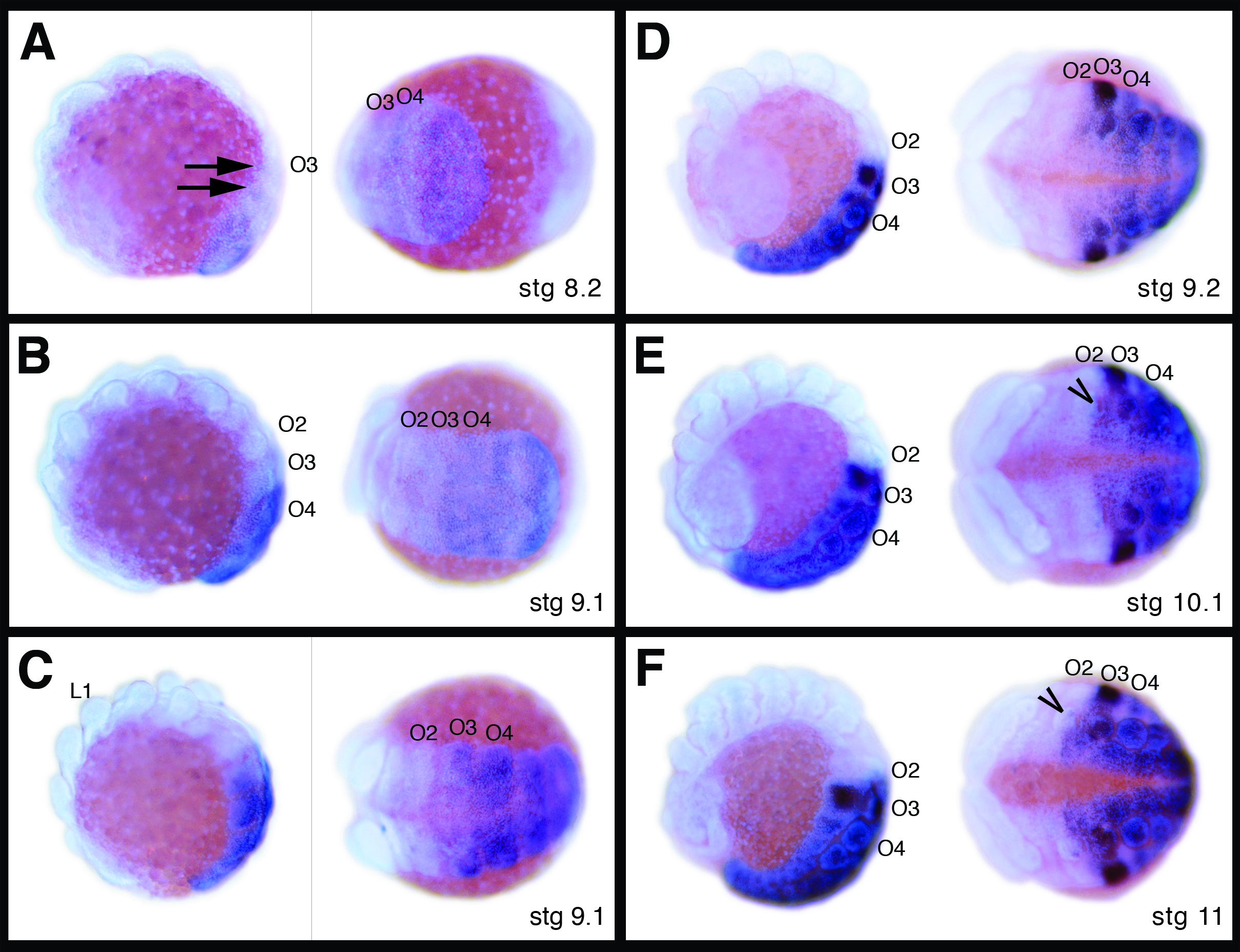

Supplement: Supplementary file 39 — P. tepidariorum Ultrabithorax-B expression. Ubx-B is first expressed at stage 8.2, in the SAZ, as well as in two weaker stripes in the O3 and O4 segments (arrows) (A). Expression is initially restricted to the posterior part of O3 (B), but eventually the anterior expression border extends ventrally into the posterior part of O2 (C–F), and is also found in the posterior part of the O2 limb bud (caret in E and F). Expression is strongest in O3, but otherwise is fairly uniform in all segments posterior to this one (D–F). Each panel shows the same embryo, viewed laterally (left) and ventrally (right). Anterior is to the left. Abbreviations: See Additional file 27: Figure S11. (JPG 4129 kb) [file 12915_2017_399_MOESM39_ESM.jpg]

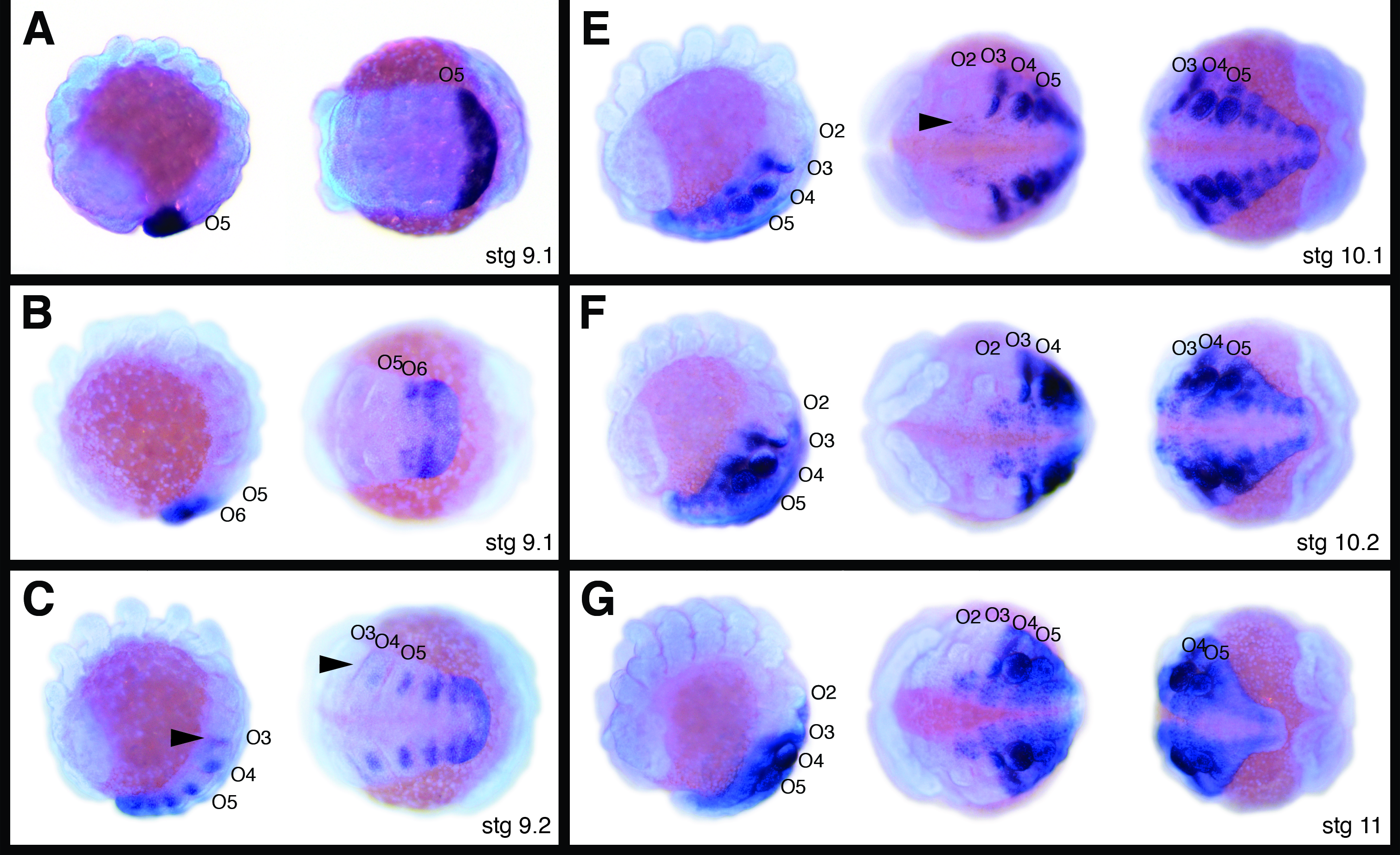

Supplement: Supplementary file 40 — P. tepidariorum abdominalA-A expression. abdA-A expression starts in the SAZ during stage 9.1, posterior to O5 (A and B). By stage 10.1, the anterior border of abdA-A expression has extended into the posterior part of O3 (arrowhead, C). While the dorsal border of abdA-A expression remains there, in the ventral neuroectoderm, the expression expands anteriorly into the posterior half of O2 (arrowhead, D). These borders of expression persist during later development (E to G). Each panel shows the same embryo, viewed laterally (left) and ventrally (right). Anterior is to the left. Abbreviations: See Additional file 27: Figure S11. (JPG 5068 kb) [file 12915_2017_399_MOESM40_ESM.jpg]

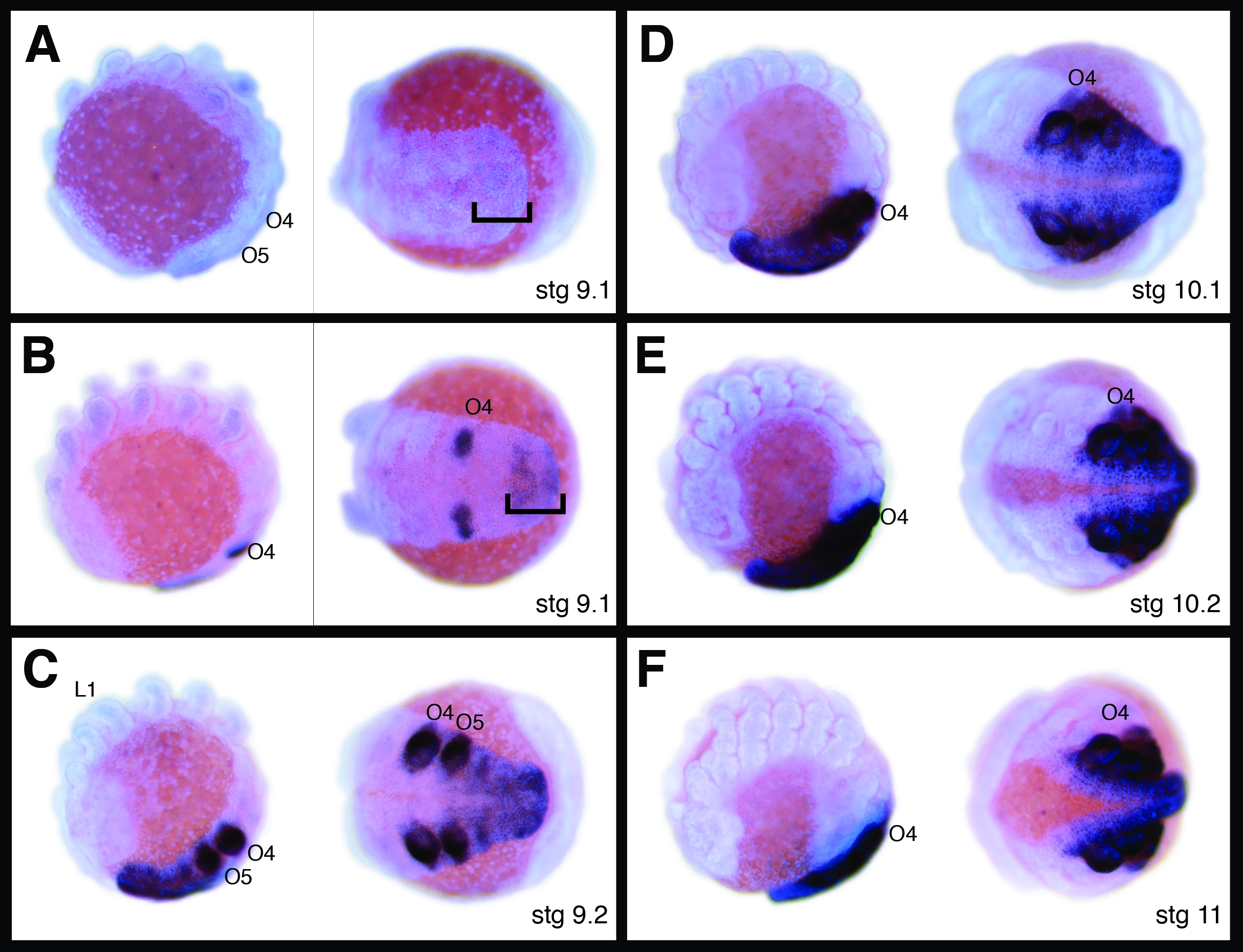

Supplement: Supplementary file 41 — P. tepidariorum abdominalA-B expression. abdA-B expression first appears at stage 9.1 in the SAZ (A). Slightly later, shortly before the opisthosomal limb buds appear, abdA-B additionally emerges in the posterior part of O4 (B). From stage 9.2 onwards, abdA-B is expressed in the entire O4 segment and all posterior segments with strong expression in the opisthosomal limb buds (C–F). Each panel shows the same embryo, viewed laterally (left) and ventrally (right). Anterior is to the left. Abbreviations: See Additional file 27: Figure S11. (JPG 4092 kb) [file 12915_2017_399_MOESM41_ESM.jpg]

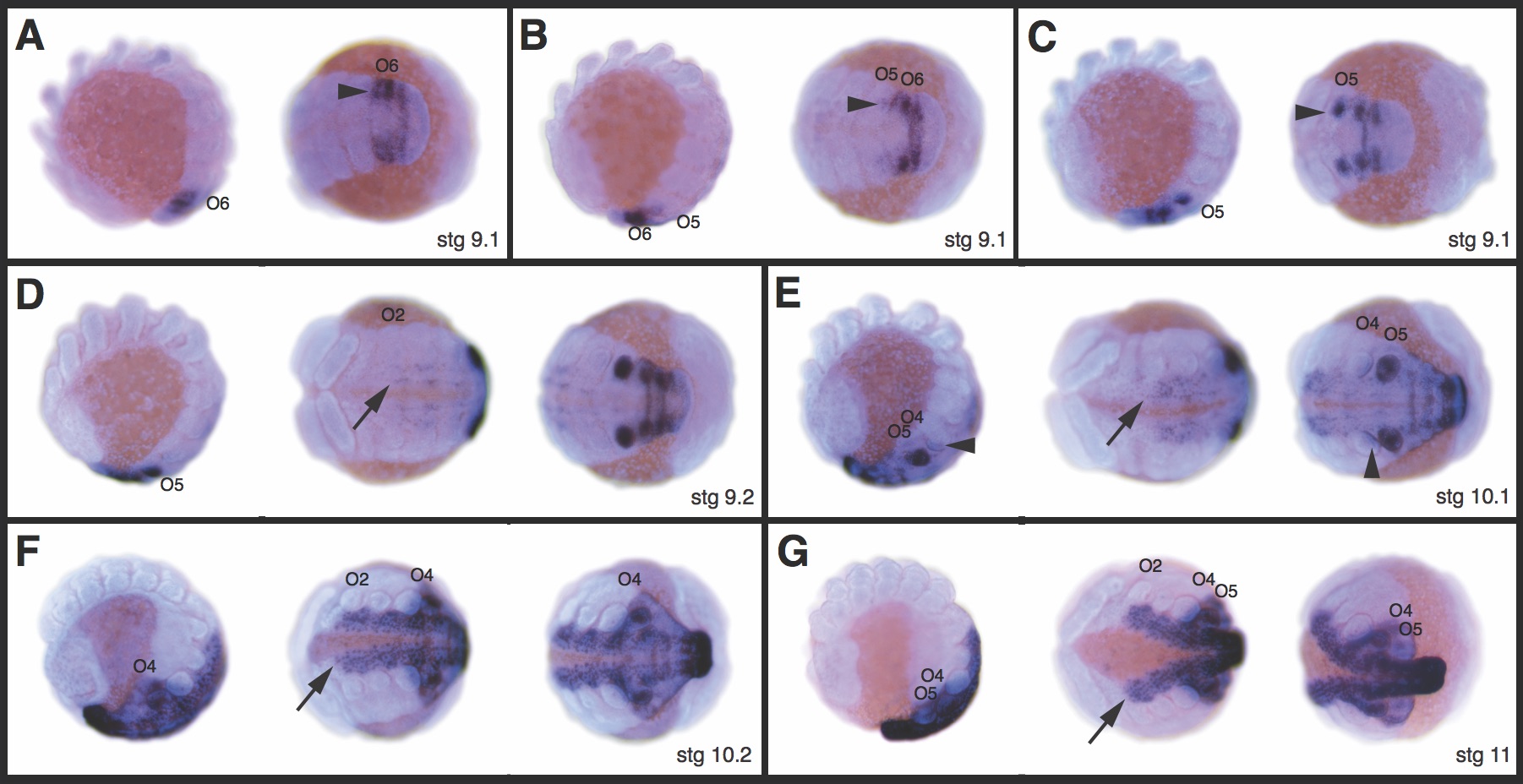

Supplement: Supplementary file 42 — P. tepidariorum AbdominalB-A expression. AbdB-A is first expressed at stage 9.1 in O6 (arrowhead) and in a stripe in the anterior-most portion of the SAZ (A). The anterior expression border subsequently shifts anteriorly, first into the posterior part of O5 (arrowhead) (B and C), and the dorsal part of the AbdB-A domain then shifts into the posterior portion of O4 (arrowhead) (D and E). From the beginning of opisthosomal limb bud development, it is strongly expressed in the O5 limb buds. At stage 9.2, the ventral part of the AbdB-A domain expands into the posterior half of O2 (arrow). These anterior borders remain the same throughout the rest of embryonic development (E–G). Each panel shows the same embryo, viewed laterally (left) and ventrally (right, or center and right in D–G). Anterior is to the left. Abbreviations: See Additional file 27: Figure S11. (JPG 245 kb) [file 12915_2017_399_MOESM42_ESM.jpg]

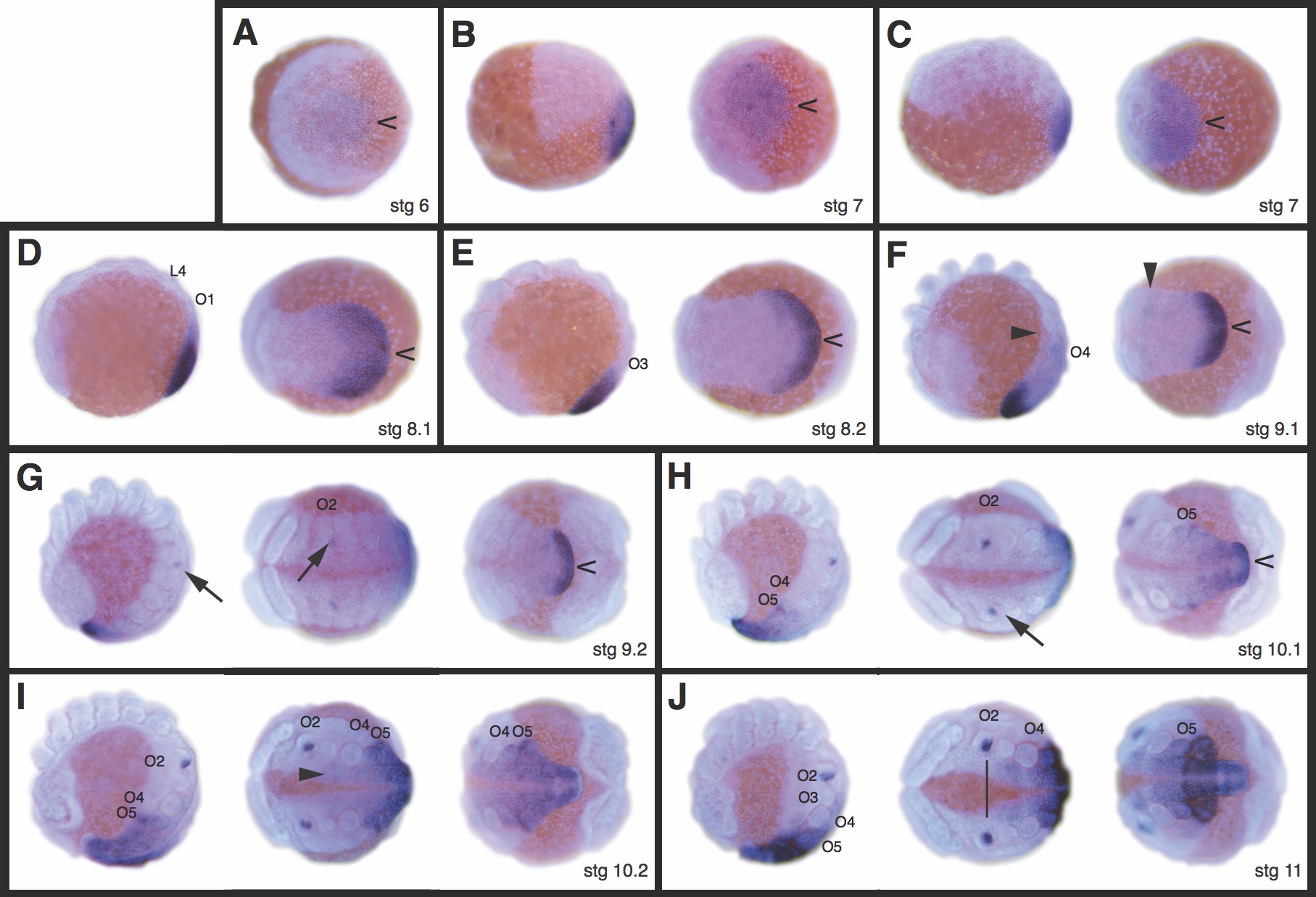

Supplement: Supplementary file 43 — P. tepidariorum AbdominalB-B expression. AbdB-B expression emerges with the appearance of the SAZ at stage 6 (A). It is continuously expressed in the SAZ until the end of segmentation (caret) (B–H). Additionally, from stage 9.1 on, weak AbdB-B expression is found posterior to the O3/O4 border (arrowhead) (F). Slightly later, additional expression appears in the O2 limb buds, in the prospective genital opening (arrow) (G and H). Dorsally, the AbdB-B expression domain extends from O5 to the posterior end (H–J), but in the ventral neuroectoderm, the AbdB-B expression border is located in the posterior half of O3 (arrowhead in I). The vertical line delineates the O2/O3 border, which the AbB-B expression does not reach in the neuroectoderm (J). Each panel shows the same embryo, viewed laterally (left) and ventrally (right, or center and right in G–J). Anterior is to the left. Abbreviations: See Additional file 27: Figure S11. (JPG 332 kb) [file 12915_2017_399_MOESM43_ESM.jpg]
