# Supplementary material for: The house spider genome reveals an ancient whole-genome duplication during arachnid evolution
Source: BMC Biol. 2017 Jul 31;15:62. doi: 10.1186/s12915-017-0399-x (PMC5535294; doi:10.1186/s12915-017-0399-x)
Supplement: Supplementary file 10 — microRNAs and other genes identified in the P. tepidariorum Hox clusters. (DOCX 106 kb) [file 12915_2017_399_MOESM10_ESM.docx]

| **Table S5: MicroRNAs and putative non-Hox genes in *Parasteatoda* Hox clusters** | | | | | | | | | | | |
| --- | --- | --- | --- | --- | --- | --- | --- | --- | --- | --- | --- |
| **CLUSTER A** | | | | | | **CLUSTER B** | | | | | |
| **Scaffold** | **AUG3 ID** | **Type** | **Coordinates** | | **Strand** | **Scaffold** | **AUG3 ID** | **Type** | **Coordinates** | | **Strand** |
| Scf5bK3_833 | aug3.g17092 | Protein | 12404 | 50863 | + | Scf5bK3_3652 | aug3.g27132 | Protein | 4364 | 14419 | - |
| Scf5bK3_833 | aug3.g23293 | Protein | 69343 | 75684 | + | Scf5bK3_3652 | aug3.g27131 | Protein | 18899 | 54219 | + |
| Scf5bK3_833 | aug3.g17627 | Protein | 106347 | 110612 | - | Scf5bK3_3652 | aug3.g24186 | Protein | 194154 | 194351 | - |
| Scf5bK3_833 | aug3.g17626 | Protein | 118772 | 133977 | + | Scf5bK3_3652 | aug3.g27129 | Protein | 342794 | 356164 | - |
| Scf5bK3_833 | aug3.g17625 | Protein | 162806 | 178288 | + | Scf5bK3_3652 | aug3.g27128 | Protein | 425500 | 425817 | - |
| Scf5bK3_833 | aug3.g6147 | Protein | 218413 | 218847 | + | Scf5bK3_3652 | aug3.g27126 | Protein | 652138 | 677132 | - |
| Scf5bK3_833 | aug3.g6146 | Protein | 275778 | 276122 | + | Scf5bK3_3652 | aug3.g27125 | Protein | 695489 | 721552 | + |
| Scf5bK3_833 | aug3.g6145 | Protein | 301707 | 302738 | - | Scf5bK3_497 | aug3.g17307 | Protein | 11 | 122833 | + |
| Scf5bK3_833 | aug3.g6144 | Protein | 307126 | 322226 | + | Scf5bK3_497 | aug3.g23141 | Protein | 6902 | 7108 | - |
| Scf5bK3_833 | aug3.g6143 | Protein | 337603 | 337836 | - | Scf5bK3_497 | aug3.g17308 | Protein | 29298 | 99590 | - |
| Scf5bK3_833 | aug3.g6142 | Protein | 338753 | 523542 | - | Scf5bK3_497 | aug3.g17306 | Protein | 126667 | 132933 | + |
| Scf5bK3_833 | aug3.g15160 | Protein | 806677 | 810378 | - | Scf5bK3_497 | aug3.g17305 | Protein | 162001 | 189110 | - |
| Scf5bK3_833 | aug3.g15159 | Protein | 859738 | 860286 | - | Scf5bK3_497 | aug3.g25353 | Protein | 407286 | 422740 | - |
| Scf5bK3_833 | aug3.g15158 | Protein | 895962 | 905313 | - | Scf5bK3_497 | aug3.g25354 | Protein | 439278 | 517702 | + |
| Scf5bK3_833 | aug3.g9050 | Protein | 946875 | 971066 | - | Scf5bK3_497 | aug3.g25356 | Protein | 619530 | 620693 | - |
| Scf5bK3_833 | aug3.g9049 | Protein | 978297 | 994389 | + | Scf5bK3_497 | aug3.g25357 | Protein | 661526 | 661888 | - |
| Scf5bK3_833 | aug3.g9047 | Protein | 1203192 | 1203605 | + | Scf5bK3_497 | iab-4-1 | miRNA | 896391 | 896451 | - |
| Scf5bK3_833 | iab-4-2 | miRNA | 1315270 | 1315330 | - | Scf5bK3_497 | aug3.g25359 | Protein | 1362095 | 1413941 | + |
| Scf5bK3_833 | aug3.g15592 | Protein | 1473721 | 1510079 | + | Scf5bK3_497 | aug3.g1790 | Protein | 2245197 | 2280004 | - |
| Scf5bK3_833 | aug3.g15593 | Protein | 1556746 | 1556958 | + | Scf5bK3_497 | aug3.g1788 | Protein | 2279886 | 2337770 | - |
| Scf5bK3_833 | aug3.g15594 | Protein | 1594525 | 1711382 | - | Scf5bK3_497 | aug3.g1789 | Protein | 2323755 | 2324231 | - |
| Scf5bK3_833 | aug3.g15596 | Protein | 1841263 | 1866959 | + | Scf5bK3_497 | aug3.g1786 | Protein | 2412067 | 2412285 | - |
| Scf5bK3_833 | aug3.g15597 | Protein | 1941513 | 1941968 | - | Scf5bK3_497 | aug3.g24519 | Protein | 2514302 | 2514370 | + |
| Scf5bK3_833 | aug3.g15598 | Protein | 1951980 | 1981315 | - | Scf5bK3_497 | aug3.g17639 | Protein | 2592144 | 2592368 | + |
| Scf5bK3_833 | aug3.g15599 | Protein | 2053402 | 2054890 | - | Scf5bK3_497 | mir-10 | miRNA | 2682407 | 2682467 | - |
| Scf5bK3_833 | aug3.g15601 | Protein | 2127888 | 2128124 | + | Scf5bK3_497 | aug3.g17638 | Protein | 2676136 | 2720228 | - |
| Scf5bK3_833 | aug3.g15602 | Protein | 2358645 | 2359130 | - | Scf5bK3_497 | aug3.g14843 | Protein | 2975718 | 2983495 | + |
| Scf5bK3_833 | aug3.g15603 | Protein | 2416290 | 2446242 | + | Scf5bK3_497 | aug3.g14844 | Protein | 3078534 | 3086147 | - |
| Scf5bK3_833 | aug3.g15604 | Protein | 2567754 | 2568197 | - |  |  |  |  |  |  |
| Scf5bK3_833 | aug3.g15605 | Protein | 2589922 | 2593240 | - |  |  |  |  |  |  |
| Scf5bK3_833 | aug3.g15607 | Protein | 2705969 | 2715250 | - |  |  |  |  |  |  |
| Scf5bK3_833 | aug3.g15609 | Protein | 3058522 | 3064693 | + |  |  |  |  |  |  |
| Scf5bK3_833 | aug3.g15610 | Protein | 3075444 | 3078817 | - |  |  |  |  |  |  |
| Scf5bK3_833 | aug3.g15611 | Protein | 3088456 | 3095404 | + |  |  |  |  |  |  |
| Scf5bK3_833 | aug3.g15612 | Protein | 3098432 | 3100932 | + |  |  |  |  |  |  |
| Scf5bK3_833 | aug3.g15613 | Protein | 3123444 | 3129408 | + |  |  |  |  |  |  |
| Scf5bK3_833 | aug3.g15614 | Protein | 3138062 | 3142524 | + |  |  |  |  |  |  |
| Scf5bK3_833 | aug3.g15615 | Protein | 3147681 | 3148019 | + |  |  |  |  |  |  |
| Scf5bK3_833 | aug3.g15616 | Protein | 3152276 | 3158397 | + |  |  |  |  |  |  |
| Scf5bK3_833 | aug3.g15617 | Protein | 3177377 | 3182828 | + |  |  |  |  |  |  |
| Scf5bK3_833 | aug3.g15618 | Protein | 3186877 | 3195781 | + |  |  |  |  |  |  |
| Scf5bK3_833 | aug3.g15619 | Protein | 3198540 | 3200902 | + |  |  |  |  |  |  |
| Scf5bK3_833 | aug3.g15620 | Protein | 3207654 | 3211392 | + |  |  |  |  |  |  |
| Scf5bK3_833 | aug3.g15621 | Protein | 3228174 | 3229451 | + |  |  |  |  |  |  |
| Scf5bK3_833 | aug3.g16686 | Protein | 3260059 | 3268113 | + |  |  |  |  |  |  |
| Scf5bK3_833 | aug3.g16685 | Protein | 3301933 | 3305549 | + |  |  |  |  |  |  |
| Scf5bK3_833 | aug3.g16684 | Protein | 3313854 | 3318699 | + |  |  |  |  |  |  |
| Scf5bK3_833 | aug3.g16683 | Protein | 3331226 | 3331591 | - |  |  |  |  |  |  |
| Scf5bK3_833 | aug3.g16682 | Protein | 3342414 | 3348804 | + |  |  |  |  |  |  |
| Scf5bK3_833 | aug3.g16681 | Protein | 3379940 | 3386593 | + |  |  |  |  |  |  |
| Scf5bK3_833 | aug3.g16680 | Protein | 3395972 | 3402247 | + |  |  |  |  |  |  |
| Scf5bK3_833 | aug3.g16679 | Protein | 3422017 | 3426263 | + |  |  |  |  |  |  |
| Scf5bK3_833 | aug3.g16490 | Protein | 3483712 | 3483771 | - |  |  |  |  |  |  |
| Scf5bK3_833 | aug3.g16491 | Protein | 3510303 | 3515677 | + |  |  |  |  |  |  |
| Scf5bK3_833 | aug3.g16492 | Protein | 3539019 | 3543371 | - |  |  |  |  |  |  |
| Scf5bK3_833 | mir-993b-1 | miRNA | 3580234 | 3580294 | - |  |  |  |  |  |  |
| Scf5bK3_833 | aug3.g17571 | Protein | 3732819 | 3733390 | + |  |  |  |  |  |  |
| Scf5bK3_833 | aug3.g17572 | Protein | 3737301 | 3737669 | + |  |  |  |  |  |  |
| Scf5bK3_833 | aug3.g7955 | Protein | 4228876 | 4248959 | - |  |  |  |  |  |  |
| Scf5bK3_833 | aug3.g7956 | Protein | 4255264 | 4259480 | + |  |  |  |  |  |  |
| Scf5bK3_833 | aug3.g7957 | Protein | 4263037 | 4274104 | + |  |  |  |  |  |  |
| Scf5bK3_833 | aug3.g21962 | Protein | 4284106 | 4287839 | - |  |  |  |  |  |  |
| Scf5bK3_833 | aug3.g26787 | Protein | 4302769 | 4337764 | + |  |  |  |  |  |  |
| Scf5bK3_833 | aug3.g26786 | Protein | 4339721 | 4344763 | - |  |  |  |  |  |  |
| Scf5bK3_833 | aug3.g26785 | Protein | 4353244 | 4386496 | + |  |  |  |  |  |  |
| Scf5bK3_833 | aug3.g26784 | Protein | 4476118 | 4478640 | - |  |  |  |  |  |  |
| Scf5bK3_833 | aug3.g26783 | Protein | 4600082 | 4603716 | - |  |  |  |  |  |  |
| Scf5bK3_833 | aug3.g26782 | Protein | 4665319 | 4666197 | - |  |  |  |  |  |  |
| Scf5bK3_833 | aug3.g26781 | Protein | 4693318 | 4739518 | - |  |  |  |  |  |  |
| Scf5bK3_833 | aug3.g26780 | Protein | 4748530 | 4754495 | + |  |  |  |  |  |  |
| Scf5bK3_833 | aug3.g26779 | Protein | 4777972 | 4789197 | + |  |  |  |  |  |  |
| Scf5bK3_833 | aug3.g26778 | Protein | 4842073 | 4851719 | + |  |  |  |  |  |  |
| Scf5bK3_833 | aug3.g26777 | Protein | 4868645 | 4870330 | - |  |  |  |  |  |  |
| Scf5bK3_833 | aug3.g21104 | Protein | 4905707 | 4923417 | + |  |  |  |  |  |  |
