# Supplementary material for: The house spider genome reveals an ancient whole-genome duplication during arachnid evolution
Source: BMC Biol. 2017 Jul 31;15:62. doi: 10.1186/s12915-017-0399-x (PMC5535294; doi:10.1186/s12915-017-0399-x)
Supplement: Supplementary file 14 — Genomic and transcriptomic datasets used to build gene families. (DOCX 141 kb) [file 12915_2017_399_MOESM14_ESM.docx]

**Table S8. Genomic and transcriptomic datasets used to build gene families**

| **Organism** | **Number of Unique Proteins** | **Type of Sequencing** | **Obtained From** | **Citation** |
| --- | --- | --- | --- | --- |
| *Strigamia maritima* | 15681^1^ | Genomic | Metazoa Ensembl | https://www.hgsc.bcm.edu/arthropods/geophilimorph-centipede-genome-project |
| *Limulus polyphemus* | 17824^2^ | Transcriptome | Author | Sharma et al, 2014 |
| *Ixodes scapularis* | 20486^1^ | Genomic | VectorBase | Van Zee et al, 2007 |
| *Tetranychus urticae* | 18224^1^ | Genomic | Metazoa Ensembl | Gbric et al, 2011 |
| *Synsyphronus apimelus* | 17820^2^ | Transcriptome | Author | Sharma et al, 2014 |
| *Mesobuthus martensii* | 32016^1^ | Genomic | Supplementary Materials | Cao et al, 2013 |
| *Centruroides exilicauda* | 30465^1^ | Genomic | ftp://ftp.hgsc.bcm.edu/I5K-pilot/Bark_scorpion/ | http://www.arthropodgenomes.org/wiki/i5K |
| *Pandinus imperator* | 15898^2^ | Transcriptome | Author | Sharma et al, 2104 |
| *Liphistius malayanus* | 11221^2^ | Transcriptome | Author | Sharma et al, 2104 |
| *Megahexura fulva* | 26457^2^ | Transcriptome | Author | Bond et al, 2014 |
| *Acanthoscurria geniculata* | 73821^2^ | Transcriptome | Supplementary Materials | Sanggaard et al, 2014 |
| *Brachythele longitarsis* | 15175^2^ | Transcriptome | Author | Bond et al, 2014 |
| *Kukulcania hibernalis* | 17219^2^ | Transcriptome | Author | Bond et al, 2014 |
| *Hypochilus pococki* | 13810^2^ | Transcriptome | Author | Bond et al, 2014 |
| *Scytodes thoracica* | 29445^2^ | Transcriptome | Author | Bond et al, 2014 |
| *Stegodyphus mimosarum* | 26869 | Genomic | Supplementary Materials | Sanggaard et al, 2014 |
| *Badumna longinqua* | 36907^2^ | Transcriptome | Author | Bond et al, 2014 |
| *Uloborus sp* | 44216^2^ | Transcriptome | Author | Bond et al, 2014 |
| *Leucauge venusta* | 17591^2^ | Transcriptome | Author | Sharma et al, 2014 |
| *Frontinella communis* | 18978^2^ | Transcriptome | Author | Sharma et al, 2014 |
| *Neoscona arabesca* | 16594^2^ | Transcriptome | Author | Sharma et al, 2014 |
| *Latrodectus geometricus* | 152807^3^ | Transcriptome | Author | Clarke et al, 2015 |
| *Latrodectus hesperus* | 191314^3^ | Transcriptome | Author | Clarke et al 2014 |
| *Steatoda grossa* | 161843^3^ | Transcriptome | Author | Clarket et al, *submitted* |
| *Parasteatoda tepidariorum* | 32186^4^ | Genomic | aug3 | this study |
| *Mastigoproctus giganteus* | 17674^2^ | Transcriptome | Author | Sharma et al, 2014 |
| *Damon variegatus* | 11823^2^ | Transcriptome | Author | Sharma et al, 2014 |
| *Metasiro americanus* | 16556^2^ | Transcriptome | Author | Sharma et al, 2014 |
| *Vonones ornata* | 19208^2^ | Transcriptome | Author | Sharma et al, 2014 |
| *Phalangium opilio* | 15277^2^ | Transcriptome | Author | Sharma et al, 2014 |
| *Trogulus martensi* | 12765^2^ | Transcriptome | Author | Sharma et al, 2014 |
| *Eremobates sp.* | 11765^2^ | Transcriptome | Author | Sharma et al, 2014 |
| *Pseudocellus sp.* | 5922^2^ | Transcriptome | Author | Sharma et al, 2014 |
| *Ricinoides atewa* | 14324^2^ | Transcriptome | Author | Sharma et al, 2014 |

1. Number of unique protein sequence from translated coding sequences obtained from publically available sources
2. Number of unique transdecoder-transcribed sequences after reducing Trinity contigs with multiple contigs to a single sequence and multiple proteins with > 95% identity to a single sequence
3. Number of unique translated sequences after reducing Trinity contigs with multiple contigs to a single sequence, combining similar nucleotide sequences (>98% Identity), and removing proteins with > 99% blastclust identity to another amino acid sequence
4. Number of unique translated sequences from the Augustus gene models
