# Supplementary material for: The house spider genome reveals an ancient whole-genome duplication during arachnid evolution
Source: BMC Biol. 2017 Jul 31;15:62. doi: 10.1186/s12915-017-0399-x (PMC5535294; doi:10.1186/s12915-017-0399-x)
Supplement: Supplementary file 16 — P values of Kolmogorov–Smirnov goodness-of-fit tests for five models of the HKY distance1 distribution of duplication nodes with non-P. tepidariorum descendants. Distributions unable to be rejected at an alpha level of 0.05 are in bold. Best fitting models are in italics. (DOCX 80 kb) [file 12915_2017_399_MOESM16_ESM.docx]

**Table S9. P-values of Kolmogorov-Smirnov goodness of fit tests for five models of the HKY distance^1^ distribution of duplication nodes with non-*P. tepidariorum* descendants.** Distributions unable to be rejected at an alpha level of 0.05 are in bold. Best fitting models are in italics.

|  | **Uniform^2^** | **Exponential^3^** | **Gaussian**  **1 peak^4^** | **Gaussian**  **2 peaks^4^** | **Gaussian**  **3 peaks^4^** |
| --- | --- | --- | --- | --- | --- |
| *P. tepidariorum* | 1.09E-14 | 4.42E-08 | 2.14E-3 | **0.0648** | ***0.8365*** |
| *L. hesperus* | 0 | 5.70E-14 | 0 | 0.0028 | ***0.1755*** |
| *L. geometricus* | 0 | 4.90E-05 | 0 | 0.0003 | *0.0006* |
| *Steatoda* | 0 | 1.29E-05 | 2.40E-13 | 4.62E-05 | ***0.5407*** |
| *Stegodyphus* | 1.27E-11 | 2.00E-06 | 0.0029 | **0.4480** | ***0.4885*** |
| *Acanthascurria* | 2.22E-16 | 0.0307 | 6.33E-09 | 0.0018 | ***0.3174*** |
| *Centruroides* | 0 | 0.0188 | 5.82E-11 | 0.0044 | ***0.0838*** |
| *Tetranychus* | 0 | *0.0101* | 0 | 6.33E-12 | 6.56E-05 |
| *Ixodes* | **0.0515** | 1.15E-05 | 0.0002 | ***0.8267*** | **0.7661** |
|  |  |  |  |  |  |
| All Duplications ^5^ | 0 | 0 | 0 | 5.81E-13 | *0.0006* |

1. HKY distances were calculated by averaging the branch lengths from duplication node to the species specified.
2. Unitorm distribution calculated using the R uniform CDF fit to the HKY distance.
3. Exponential distribution calculated with the R exponential CDF with the rate calculated from the HKY distances.
4. The mean and standard deviations for the Gaussian peaks were derived form Gaussian distributions fit to the *P. tepidariorum* HKY distribution from random starting with the specified number of peaks. The mixture percentages for each species were re-calculated prior to performing K-S tests.
5. All duplications nodes with at least one *P. tepidariorum* descendent.
