# Supplementary material for: The house spider genome reveals an ancient whole-genome duplication during arachnid evolution
Source: BMC Biol. 2017 Jul 31;15:62. doi: 10.1186/s12915-017-0399-x (PMC5535294; doi:10.1186/s12915-017-0399-x)
Supplement: Supplementary file 18 — Percent of duplication nodes assigned to three Gaussian distributions of HKY distances. The mean and standard deviation of the P. tepidariorum distributions (Fig. 6a) were used to estimate the other species’ Gaussian distributions. (DOCX 65 kb) [file 12915_2017_399_MOESM18_ESM.docx]

**Table S10. Percent of duplication nodes assigned to three Gaussian distributions of HKY distances.** The mean and standard deviation of the *Parasteatoda* distributions (Figure 6a) were used to estimate the other species’ Gaussian distributions.

| **Species** | **Number of Paralog pairs^1^** | **Young^2^** | **Middle^3^** | **Old^4^** |
| --- | --- | --- | --- | --- |
| *Parasteatoda* | 486 | 0.1290 | 0.4472 | 0.4239 |
| *L. hesperus* | 792 | 0.1465 | 0.5440 | 0.3094 |
| *L. geometricus* | 686 | 0.1664 | 0.5433 | 0.2903 |
| *S. grossa* | 546 | 0.1722 | 0.4790 | 0.3488 |
| *Stegodyphus* | 237 | 0.0772 | 0.5339 | 0.3889 |
| *Acanthoscurria* | 253 | 0.1786 | 0.4932 | 0.3282 |
| *Bark Scorpion* | 295 | 0.1812 | 0.4951 | 0.3237 |
| *Tetranychus* | 347 | 0.2262 | 0.4659 | 0.3080 |
| *Ixodes* | 189 | 0.0551 | 0.3911 | 0.5538 |
| Total | 1362 | 0.0460 | 0.6170 | 0.337 |
|  |  |  |  |  |

1. Number of duplication nodes with at least two descendants in the species of interest.
2. Proportion of duplication nodes assigned to the youngest distribution (μ=0.04, σ=0.02, λ=0.12).
3. Proportion of duplication nodes assigned to the middle distribution (μ=0.49, σ=0.22, λ=0.46).
4. Proportion of duplication nodes assigned to the oldest distribution (μ=1.3, σ=0.35, λ=0.42).
