# Supplementary material for: The house spider genome reveals an ancient whole-genome duplication during arachnid evolution
Source: BMC Biol. 2017 Jul 31;15:62. doi: 10.1186/s12915-017-0399-x (PMC5535294; doi:10.1186/s12915-017-0399-x)
Supplement: Supplementary file 20 — Comparison of distributions for P. tepidariorum duplication and speciation nodes. Kolmogorov–Smirnov goodness-of-fit tests were used to compare the middle Gaussian distribution of HKY distances of duplication nodes (Fig. 6a, Additional file 13: Figure S6) to the log-normal distributions for speciation nodes. (DOCX 73 kb) [file 12915_2017_399_MOESM20_ESM.docx]

**Table S11. Comparison of distributions for *P. tepidariorum* duplication and speciation nodes.** Kolmogorov-Smirnov goodness of fit tests were used to compare the middle Gaussian distribution of HKY distances of duplication nodes (Figure 6a, Supplementary Figure S6) to the log-normal distributions for speciation nodes.

| **Speciation Node^1^** | ***Parasteatoda* K-S P-Value^2^** | ***Centruroides* K-S P-value^3^** |
| --- | --- | --- |
| P.—*Latrodectus* | 0 | NA |
| P.—*Frontinella* | 0 | NA |
| P.—*Badumna* | 0 | NA |
| P.—*Stegodyphus* | 0 | NA |
| P.—Haplogynae | 7.77E-16 | NA |
| P.—Mygalomorphae | 1.63E-05 | NA |
| P.—*Liphistius* | 0.030283556 | NA |
| P.—Uropygi | 0.009097995 | NA |
| P.—Scorpionida/C.—Araneae | 1.27E-05 | 0.0524 |
| P./C.— *Synsphyronus* | 1.62E-06 | 0.0020 |
| P./C.—Opiliones | 1.54E-07 | 0.0003 |
| P./C.—*Tetranychus* | 7.49E-09 | 7.79E-05 |
| P./C.—*Ixodes* | 1.11E-16 | 4.84E-07 |
| P./C.—*Limulus* | 7.62E-10 | 4.93E-05 |
| P./C.—*Strigamia* | 0 | 2.91E-09 |

1. Speciation node between *Parasteatoda (*P.) or *Centruroides (*C.) and the specified species or set of species used to create the log-normal distribution using *Parasteatoda* and *Centruroides-*seeding family respectively.
2. P-value calculated from log-normal distribution estimated using mean and standard deviation of the natural log of the HKY distances between *Parasteatoda* and the specified speciation node.
3. P-value calculated from log-normal distribution estimated using mean and standard deviation of the natural log of the HKY distances between *Centruroides* and the specified speciation node.
