# Supplementary material for: The house spider genome reveals an ancient whole-genome duplication during arachnid evolution
Source: BMC Biol. 2017 Jul 31;15:62. doi: 10.1186/s12915-017-0399-x (PMC5535294; doi:10.1186/s12915-017-0399-x)
Supplement: Supplementary file 21 — Shared paralog pair retention between P. tepidariorum and other arthropod species. Shaded species have complete genomes or deeply sequenced transcriptomes. (DOCX 92 kb) [file 12915_2017_399_MOESM21_ESM.docx]

**Table S12. Shared paralog pair retention between *P. tepidariorum* and other arthropod species.** Shaded species have complete genomes or deeply sequenced transcriptomes.

| **Species** | **# shared retained paralogs^1^** | **# shared in middle Gaussian distribution^2^** | **# of duplication nodes^3^** | **Percent shared retained paralogs^4^** |
| --- | --- | --- | --- | --- |
| *L. hesperus* | 291 | 129 | 396 | 73% |
| *L. geometricus* | 253 | 105 | 379 | 67% |
| *Steatoda* | 265 | 116 | 392 | 68% |
| *Stegodyphus* | 154 | 68 | 325 | 47% |
| *Acanthoscurria* | 109 | 31 | 265 | 41% |
| *Centruroides* | 86 | 21 | 253 | 34% |
| *Mesobuthus* | 29 | 9 | 161 | 18% |
| *Tetranychus* | 71 | 4 | 199 | 36% |
| *Ixodes* | 72 | 3 | 201 | 36% |
| *Strigamia* | 98 | 9 | 210 | 47% |
| *Neoscona* | 30 | 11 | 158 | 19% |
| *Frontinella* | 42 | 14 | 185 | 23% |
| *Badumna* | 7 | 0 | 85 | 8% |
| *Scytodes* | 10 | 3 | 97 | 10% |
| *Hypochlius* | 2 | 1 | 30 | 7% |
| *Kukucania* | 6 | 2 | 69 | 9% |
| *Brachythele* | 4 | 3 | 41 | 10% |
| *Megahexura* | 14 | 6 | 113 | 12% |
| *Liphustius* | 19 | 5 | 112 | 17% |
| *Damon* | 4 | 1 | 73 | 5% |
| *Mastigoproctus* | 19 | 8 | 142 | 13% |
| *Pandinus* | 14 | 4 | 135 | 10% |
| *Synsphyronus* | 21 | 4 | 100 | 21% |
| *Vonones* | 25 | 4 | 164 | 15% |
| *Phalangium* | 8 | 1 | 81 | 10% |
| *Trogulus* | 37 | 5 | 160 | 23% |
| *Metasiro* | 11 | 1 | 100 | 11% |
| *Pseudocellus* | 1 | 0 | 27 | 4% |
| *Eremobates* | 2 | 0 | 56 | 4% |
| *Ricinoides* | 8 | 1 | 80 | 10% |
| *Limulus* | 20 | 3 | 110 | 18% |

1. Number of duplication nodes that give rise to a paralog pair both in *Parasteatoda* and the species
2. Number of duplication nodes that give rise to a paralog pair both in *Parasteatoda* and the species and were assigned to the middle Gaussian distribution of HKY distances (Figure 6a).
3. Total number of duplication nodes that give rise to a *Parasteatoda* paralog pair and at least one gene from the species.

^4^Percentage of total duplication nodes^3^ that give rise to a paralog pair^1^ in *Parasteatoda* and the species.
