# Supplementary material for: The house spider genome reveals an ancient whole-genome duplication during arachnid evolution
Source: BMC Biol. 2017 Jul 31;15:62. doi: 10.1186/s12915-017-0399-x (PMC5535294; doi:10.1186/s12915-017-0399-x)
Supplement: Supplementary file 44 — Hox gene expression in P. tepidariorum. Detailed description and comparison of the expression patterns of the Hox gene paralogs. (DOCX 143 kb) [file 12915_2017_399_MOESM44_ESM.docx]

**Supplementary File 1: Hox gene expression in *P. tepidariorum.***

*lab-A* expression [[1](#_ENREF_1), [2](#_ENREF_2)] appears much later in development than *lab-B* [[2](#_ENREF_2)], at stage 6 compared to stage 4. While both genes are most highly expressed in the pedipalpal segment (Pp), the *lab* paralogs differ in their posterior borders: *lab-B* expression covers Pp and the first two walking leg segments (L1 and L2), whereas *lab-A* is additionally expressed in the two remaining walking leg segments L3 and L4. In addition, expression in the ventral neuroectoderm and the developing legs differs between the two paralogs.

The expression of *pb-B* (Additional file 28: Fig. S12) also appears much later, at stage 8.2, whereas *pb-A* expression (Additional file 27: Fig. S11) already emerges at stage 7. In addition, *pb-B* is expressed at lower levels, and its expression appears in Pp to L4 at the same time, whereas *pb-A* is initially restricted to Pp and then expands into L1-L4 and O1. Furthermore, while *pb-B* is mostly expressed in the mesoderm of the appendages, *pb-A* is also expressed in an ectodermal domain in the distal tips of the appendages. Finally, the expression in the neuroectoderm also differs, as different cell groups of each hemisegment express *pb-B* and *pb-A* (compare e.g. the expression in the neuroectoderm of the pedipalpal segment in the central panels of Additional files 27, 28: Figs S11H, S12G).

*Hox3-B* expression appears later than *Hox3-A* expression (Additional files 29, 30: Figs S13, S14), at stage 8 in broad segmental stripes in Pp-L4 (Additional file 30: Fig. S14A, B). Its expression is much stronger than that of *Hox3-A*. The expression borders of *Hox3-B* remain unchanged until stage 11, when expression additionally appears in a few segmental dots in the neuroectoderm of the opisthosoma and in the opisthosomal appendages (Additional file 30: Fig. S14F). *Hox3-A* however, is mostly expressed in mesodermal tissues (Additional file 29: Fig. S13), first in segmental stripes, and then in the prosomal limb buds, before the expression disappears after stage 9. Due to the very weak staining of *Hox3-A* we were not able to determine the exact expression domain, but it covers a similar domain as *Hox3-B*, ranging from the pedipalpal segment across all four walking leg segments.

*Dfd-A* expression (Additional file 31: Fig. S15) appears at stage 4 and covers almost all tissue of the germ disc but the most anterior ring [[1](#_ENREF_1)], the future pedipalpal and cheliceral segment [[3](#_ENREF_3)]. *Dfd-B* is first expressed at stage 7 (Additional file 32: Fig. S16), and it is restricted to the presumptive L2/L3 region, but it later expands into L3 and L4. However, in contrast to *Dfd-A*, which is expressed strongly in all walking leg appendages, *Dfd-B* is expressed only in the ventral neuroectoderm.

*Scr-B* is expressed at stage 5 already (Fig. 9B, Additional file 34: Fig. S18), one stage before *Scr-A* expression appears (Fig. 9E, Additional file 33: Fig. S17). The two paralogs also differ in their expression domains; the anterior border of *Scr-A* is shifted by half a segment towards the posterior, but therefore its expression later expands all throughout the opisthosoma, whereas *Scr-B* only reaches into O1 and O2. Besides these differences, the ring-like pattern in the legs differs tremendously between the two paralogs (Fig. 9D).

*ftz* (Additional file 35: Fig. S19) is first expressed at stage 6. It is expressed in two domains, (1) a comparatively weak expression domain that can later be mapped onto segments L2-L4 (Additional file 35: Fig. S19B-F) that is most prominent in the neuroectoderm of L2-L4 (Additional file 35: Fig. S19E, F and H) as well as in the tips of the L3 appendage at later stages (Additional file 35: Fig. S19G and H); (2) a strong expression domain in the forming segment addition zone (SAZ) (Additional file 35: Fig. S19A). For every new opisthosomal segment that is formed, one new stripe of *At-ftz* expression buds off from the anterior SAZ. This stripe then fades, so that maximally two stripes are visible; one that is just forming in the anterior SAZ and one that is fading at the anterior border of the afore generated segment. This expression in the SAZ persists until no more segments are formed at stage 11 (Additional file 35: Fig. S19H). Even though the anterior and posterior expression border of this Hox-like expression domain closely resembles *Cs-ftz* in *Cupiennius salei* [[4](#_ENREF_4)]*,* the expression pattern in the neuroectoderm is different from *C. salei*. In *P. tepidariorum*, *ftz* is expressed more uniformly compared to *Cs-ftz*, which is only expressed in a few specific cells per hemisegment in the *Cupiennius* neuroectoderm. *Pt-ftz* is also expressed in the legs, but only in L3, and the expression pattern in the legs differs from *Cs-ftz* as well. These differences between the expression of the *ftz* orthologs in *C. salei* and *P. tepidariorum* might be interpreted as further evidence for the existence of a second copy of *ftz* in these spiders.

*Antp-A* (Additional file 36: Fig. S20) is already expressed during germ band formation at stage 7. It is initially only expressed in the presumptive O1 segment, but then broad expression is found in every segment added from the SAZ. The expression later extends also into the posterior half of L4, and fades in most parts of the opisthosoma, while still remaining strongly expressed in O1 and the anterior half of O2. *Antp-B* (Additional file 37: Fig. S21) on the other hand is expressed later, at stage 8.2, and it is expressed first only in O1 and O2, and then expands anteriorly into the posterior half of L4, covering a very similar domain as *Antp-A*, yet differing in the exact pattern in the developing legs and the neuroectoderm. Interestingly, during inversion (stage 11), *Antp-B* expression can be found in the neuroectoderm of all appendage-bearing segments and the opisthosoma.

*Ubx-A* expression (Additional file 38: Fig. S22) appears slightly later than *Ubx-B*, at stage 8.2, with an anterior expression border in the posterior half of O2. It continues to be expressed in all segments posterior to this border and at later stages expands also into the anterior half of O2. *Ubx-B* first shows low expression in the presumptive O3 region at stage 8.1 (Additional file 39: Fig. S23). It then forms a stripe in this segment and subsequently appears in all segments posterior to O3. During later development, *Ubx-B* also extends anteriorly into the posterior half of O2, while being most strongly expressed in O3 at these stages.

*abdA-A* (Additional file 40: Fig. S24) is expressed at stage 8.2 in O6, whereas *abdA-B* expression (Additional file 41: Fig. S25) starts slightly later, in two separate domains in the posterior part of O4 and in O6. Both genes then extend their expression into all segments posterior and also slightly anterior to their initial appearance, *abdA-A* into O3 and *abdA-B* into O4.

*AbdB-A* expression (Additional file 42: Fig. S26) appears at stage 8.2 in segment O6. From there the expression slowly shifts more anteriorly, in the ventral neuroectoderm it eventually reaches into the posterior half of O2, whereas dorsally the expression border finally ends up in the posterior part of O4. In contrast to *AbdB-A*, *AbdB-B* (Additional file 43: Fig. S27) shows a very dynamic expression pattern. Moreover, if one takes Hox gene collinearity rules into account, whereby genes located more posteriorly in the Hox gene cluster are expressed later during development, *AbdB-B* does not follow this rule. Instead, it is expressed in the SAZ during its formation at stage 6. *AbdB-B* expression stays in the SAZ until the end of segmentation, and is switched off in the segments already formed. Only later, at stage 9.1, weak *AbdB-B* expression starts to appear in O4 and segments posterior to it. While the O4 border shifts only slightly into the posterior part of O3 in the neuroectoderm, with the appearance of the opisthosomal limb buds at stage 9.2, *AbdB-B* is additionally expressed in the O2 limb bud, the future genital opening.

1. Akiyama-Oda Y, Oda H: **Cell migration that orients the dorsoventral axis is coordinated with anteroposterior patterning mediated by Hedgehog signaling in the early spider embryo.** *Development* 2010, **137:**1263-1273.

2. Pechmann M, Schwager EE, Turetzek N, Prpic NM: **Regressive evolution of the arthropod tritocerebral segment linked to functional divergence of the Hox gene labial.** *Proc Biol Sci* 2015, **282**.

3. Kanayama M, Akiyama-Oda Y, Nishimura O, Tarui H, Agata K, Oda H: **Travelling and splitting of a wave of hedgehog expression involved in spider-head segmentation.** *Nat Commun* 2011, **2:**500.

4. Damen WG, Janssen R, Prpic NM: **Pair rule gene orthologs in spider segmentation.** *Evol Dev* 2005, **7:**618-628.
