# Supplementary material for: The house spider genome reveals an ancient whole-genome duplication during arachnid evolution
Source: BMC Biol. 2017 Jul 31;15:62. doi: 10.1186/s12915-017-0399-x (PMC5535294; doi:10.1186/s12915-017-0399-x)
Supplement: Supplementary file 22 [file 12915_2017_399_MOESM15_ESM.docx]

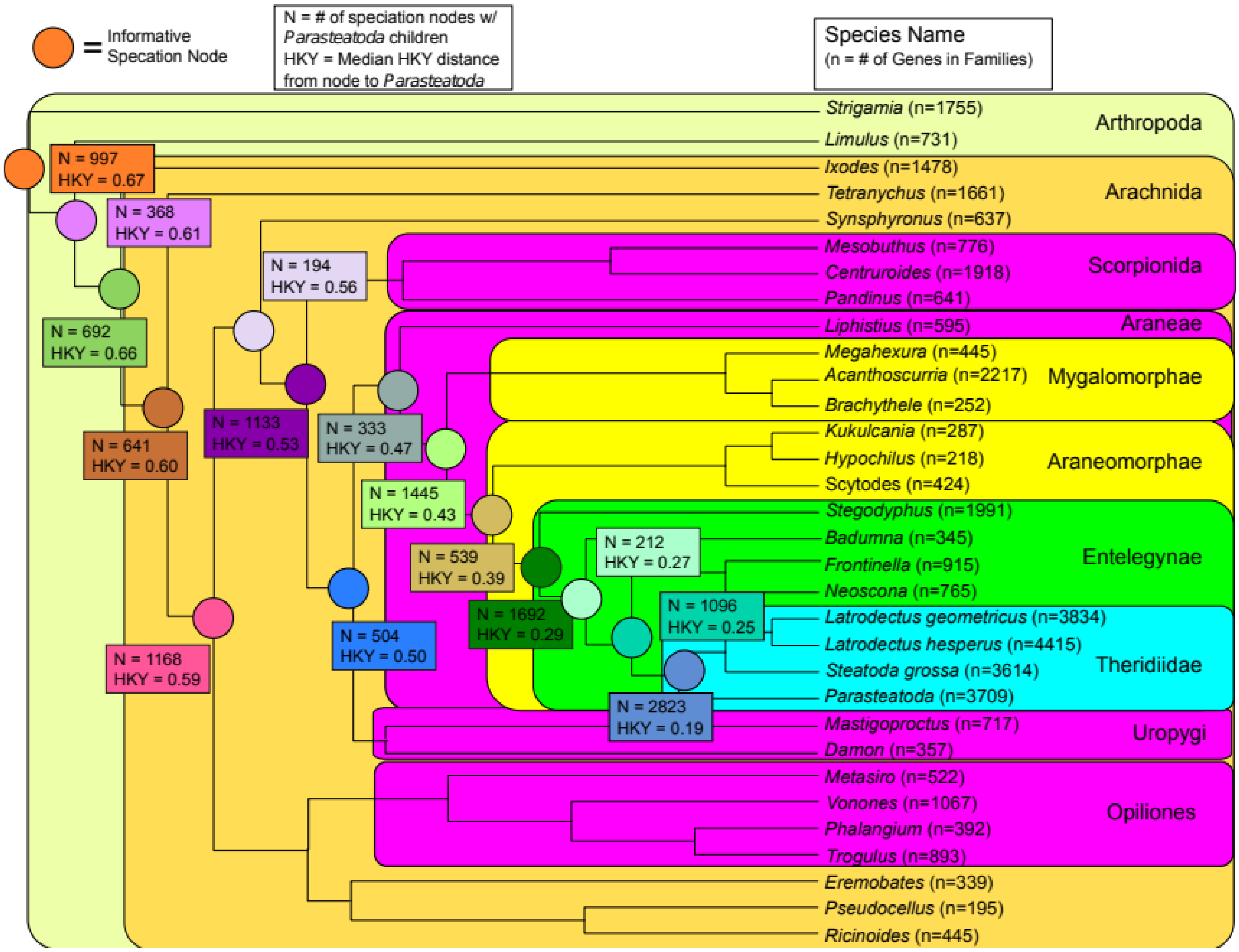

= Informative Specation Node
N = # of speciation nodes w/ Parasteatoda children
HKY = Median HKY distance from node to Parasteatoda
Species Name
(n = # of Genes in Families)
Strigamia (n=1755)
Limulus (n=731)
Ixodes (n=1478)
Tetranychus (n=1661)
Synsphyronus (n=637)
Mesobuthus (n=776)
Centruroides (n=1918)
Pandinus (n=641)
Liphistius (n=595)
Megahexura (n=445)
Acanthoscurria (n=2217)
Brachythele (n=252)
Kukulcania (n=287)
Hypochilus (n=218)
Scytodes (n=424)
Stegodyphus (n=1991)
Badumna (n=345)
Frontinella (n=915)
Neoscona (n=765)
Latrodectus geometricus (n=3834)
Latrodectus hesperus (n=4415)
Steatoda grossa (n=3614)
Parasteatoda (n=3709)
Mastigoproctus (n=717)
Damon (n=357)
Metasiro (n=522)
Vonones (n=1067)
Phalangium (n=392)
Trogulus (n=893)
Eremobates (n=339)
Pseudocellus (n=195)
Ricinoides (n=445)
Arthropoda
Arachnida
Scorpionida
Araneae
Mygalomorphae
Araneomorphae
Entelegynae
Theridiidae
Uropygi
Opiliones
N = 997 HKY = 0.67
N = 368 HKY = 0.61
N = 692 HKY = 0.66
N = 641 HKY = 0.60
N = 1168 HKY = 0.59
N = 194 HKY = 0.56
N = 1133 HKY = 0.53
N = 504 HKY = 0.50
N = 333 HKY = 0.47
N = 1445 HKY = 0.43
N = 539 HKY = 0.39
N = 1692 HKY = 0.29
N = 212 HKY = 0.27
N = 1096 HKY = 0.25
N = 2823 HKY = 0.19
