# Supplementary material for: The house spider genome reveals an ancient whole-genome duplication during arachnid evolution
Source: BMC Biol. 2017 Jul 31;15:62. doi: 10.1186/s12915-017-0399-x (PMC5535294; doi:10.1186/s12915-017-0399-x)
Supplement: Supplementary file 23 [file 12915_2017_399_MOESM19_ESM.docx]

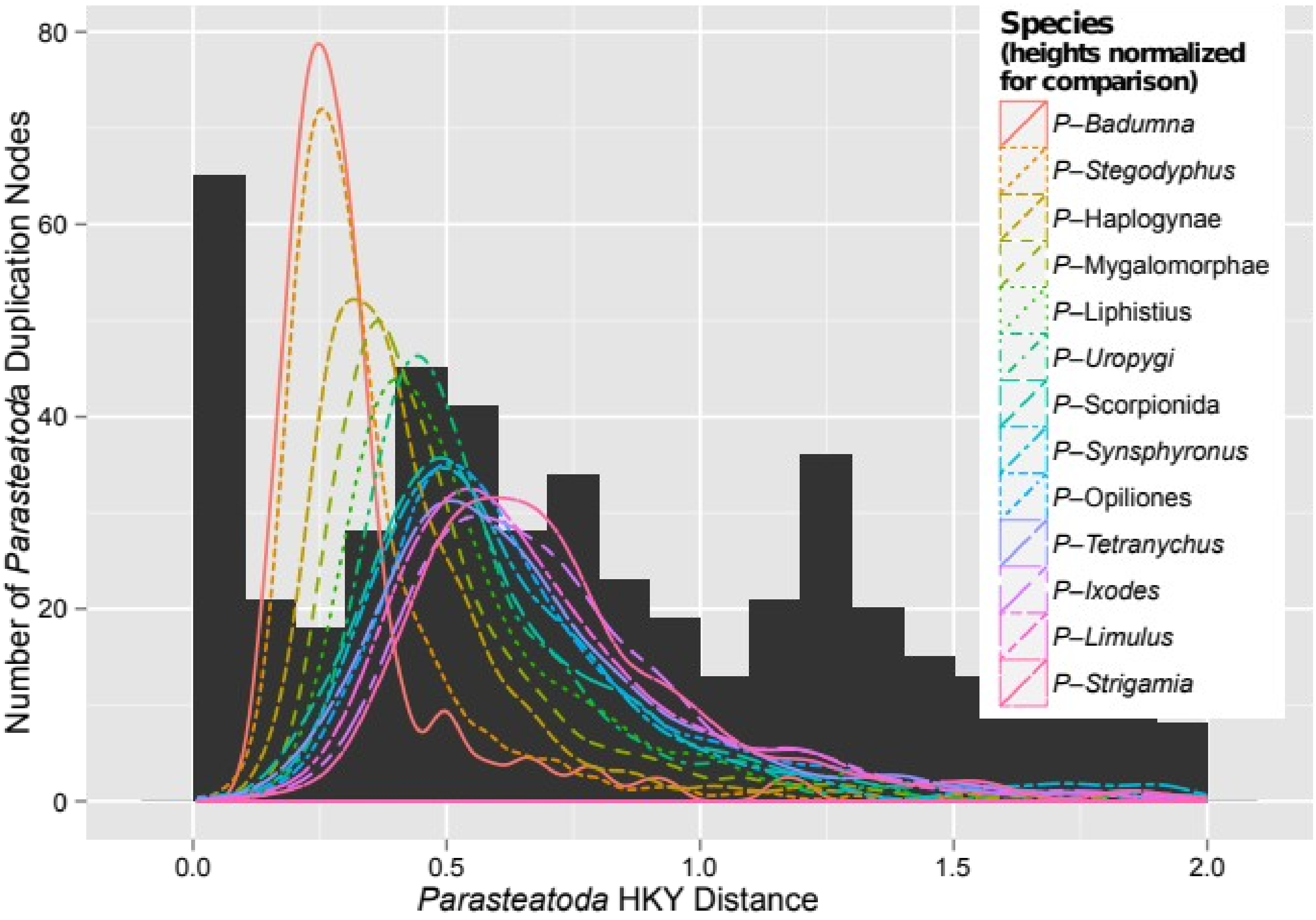
Species
(heights normalized for comparison)
P–Badumna
P–Stegodyphus
P–Haplogynae
P–Mygalomorphae
P–Liphistius
P–Uropygi
P–Scorpionida
P–Synsphyronus
P–Opiliones
P–Tetranychus
P–Ixodes
P–Limulus
P–Strigamia
Number of Parasteatoda Duplication Nodes
Parasteatoda HKY Distance
0
20
40
60
80
0.0
0.5
1.0
1.5
2.0
